# Mycorrhiza governs plant-plant interactions through preferential allocation of shared nutritional resources: A triple (^13^C, ^15^N and ^33^P) labeling study

**DOI:** 10.3389/fpls.2022.1047270

**Published:** 2022-12-15

**Authors:** Maede Faghihinia, Jan Jansa

**Affiliations:** ^1^ Laboratory of Fungal Biology, Institute of Microbiology, Czech Academy of Sciences, Praha, Czechia; ^2^ Department of Plant Pathology and Microbiology, Iowa State University, Ames, IA, United States

**Keywords:** arbuscular mycorrhizal symbiosis, plant competition and co-existence, isotopic labeling, preferential resource allocation, mineral nutrients, carbon

## Abstract

Plant-plant interactions and coexistence can be directly mediated by symbiotic arbuscular mycorrhizal (AM) fungi through asymmetric resource exchange between the plant and fungal partners. However, little is known about the effects of AM fungal presence on resource allocation in mixed plant stands. Here, we examined how phosphorus (P), nitrogen (N) and carbon (C) resources were distributed between coexisting con- and heterospecific plant individuals in the presence or absence of AM fungus, using radio- and stable isotopes. Congeneric plant species, *Panicum bisulcatum* and *P. maximum*, inoculated or not with *Rhizophagus irregularis*, were grown in two different culture systems, mono- and mixed-species stands. Pots were subjected to different shading regimes to manipulate C sink-source strengths. In monocultures, *P. maximum* gained more mycorrhizal phosphorus uptake benefits than *P.bisulcatum*. However, in the mixed culture, the AM fungus appeared to preferentially transfer nutrients (^33^P and ^15^N) to *P.bisulcatum* compared to *P. maximum*. Further, we observed higher ^13^C allocation to mycorrhiza by *P.bisulcatum* in mixed- compared to the mono-systems, which likely contributed to improved competitiveness in the mixed cultures of *P.bisulcatum* vs. *P. maximum* regardless of the shading regime. Our results suggest that the presence of mycorrhiza influenced competitiveness of the two *Panicum* species in mixed stands in favor of those with high quality partner, *P. bisulcatum*, which provided more C to the mycorrhizal networks. However, in mono-species systems where the AM fungus had no partner choice, even the lower quality partner (i.e., *P.maximum*) could also have benefitted from the symbiosis. Future research should separate the various contributors (roots vs. common mycorrhizal network) and mechanisms of resource exchange in such a multifaceted interaction.

## 1 Introduction

Understanding the factors that influence the coexistence of plant species in natural ecosystems is a central concept in plant community ecology (Bengtsson et al., 1994; Bever et al., 2010; Wilson, 2011). According to the most widely accepted theory of plant coexistence in ecological communities, only species with sufficiently different resource requirements (e.g., nutrients, light, water) and traits (e.g., rooting depth, phenology) can coexist in the long run (Gause, 1934; Connell, 1983; Aarssen, 1989; Dybzinski and Tilman, 2007). Because only a limited number of distinct niches are available in the natural environment, niche overlap can lead to negative intraspecific interactions, competition for available resources, and eventually a limited number of coexisting species (MacArthur and Levins, 1967; Tilman, 1982; Connell, 1983; Schoener, 1983). In addition to abiotic factors, biotic factors can also influence plant coexistence, such as the presence of organisms that interact positively or negatively with plants. Among those, soil microorganisms are particularly important, in spite of being little visible but having a major impact on plant coexistence, interactions, and community composition (van der Heijden et al., 2006; Vogelsang et al., 2006; Bever et al., 2010; Moora and Zobel, 2010).

Plant-plant interactions and coexistence can be directly mediated by arbuscular mycorrhizal (AM) fungi (Leake et al., 2004; Scheublin et al., 2007; Simard et al., 2012), which are ubiquitous plant root symbiotic partners in a variety of terrestrial ecosystems (Spatafora et al., 2016; Brundrett and Tedersoo, 2018). The fungal symbiont relies fully on photosynthetic carbon (C) obtained from the plant roots; in return, it provides mineral nutrients to plants, particularly phosphorus (P) and nitrogen (N), taken outside of the rhizosphere and thus out of reach for the roots themselves (Smith and Read, 2008; van der Heijden et al., 2008). The fungal partner also provides a number of non-nutritional benefits to their host, such as improving plant-water relations (Augé et al., 2015), resistance to abiotic (e.g., salinity, heavy metals, drought) and biotic (e.g., pathogens, herbivores) stresses (Kikuchi et al., 2016; Faghihinia et al., 2020; Faghihinia et al., 2021; Zai et al., 2021). Remarkably, AM fungi can colonize the roots of different plant species simultaneously and interconnect neighbouring or co-cultivated plants by forming so called common mycorrhizal networks (CMNs) in the soil (Klironomos, 2000; Selosse et al., 2006; Jakobsen and Hammer, 2015). There is compelling evidence that CMNs distribute both nutritional (e.g., transfer of nutrients) and non-nutritional benefits (transfer of defense signals or allelochemicals) between coexisting plants (Bever et al., 2010; Babikova et al., 2013; Song et al., 2014), which may eventually lead to over-yielding of plant communities (Li et al., 2022).

Interestingly, AM fungi could disproportionately affect the fitness of coexisting plants through asymmetric resource partitioning among plants (Bever et al., 2010; Weremijewicz and Janos, 2013), which can be attributed to some extent to host preference in resource exchange with a certain partner (Vandenkoornhuyse et al., 2003; Montesinos-Navarro et al., 2019). If this is fully reciprocated (which is predicted by a market theory), coexisting plants benefit from mycorrhizal symbiosis based on their corresponding C investments into their (shared) fungal partner. In other words, mycorrhiza may preferentially transfer more nutrients to the plants that provide more C and less nutrients to the plants that allocate less C to the mycobiont (Bever et al., 2009; van der Heijden and Horton, 2009; Lekberg et al., 2010; Kiers et al., 2011). However, it seems that the cost-benefit relationships between AM fungi and plants are not always interlinked. The benefits of each individual plant species from AM fungi may vary with environmental contexts, e.g., due to differences in soil type and nutrient availability (Konvalinková et al., 2015; Wang et al., 2016; Voříšková et al., 2019), plant and fungal identity (Argüello et al., 2016; Wang et al., 2016), plant size and growth stage (van der Heijden and Horton, 2009), and/or existing competition among plants belonging to the same or different species for other resources (Scheublin et al., 2007). In such cases, some plant species may invest more C in mycorrhiza while the other plants derive most benefits from the shared mycorrhizal networks (Walder et al., 2012). Nevertheless, the terms of resource exchange between plants and their shared CMNs and the influence of mycorrhizal fungi on the outcome of plant-plant interactions are not yet fully understood (van der Heijden and Horton, 2009; Montesinos-Navarro et al., 2019; Davison et al., 2020). In general, the questions of which plant species benefits most from mycorrhiza and how this is physiologically organized are still difficult to answer or predict.

The redistribution of symbiotic benefits and costs can also be attributed to the different ecological strategies of plants included in a community. Indeed, the exchanged resources such as C or P could be controlled by both plant and fungal partners (Kiers and van der Heijden, 2006; Bever et al., 2009). On this basis, coexisting plants of different species or functional groups are expected to adopt different strategies under different environmental conditions when plants are incorporated into an existing mycorrhizal network (Bever et al., 2010; Jakobsen and Hammer, 2015). There have been few empirical attempts to experimentally assess the investment in CMNs by individual plants of the same or different species or functional groups (e.g., mycorrhizal status, growth forms, photosynthetic pathways, etc.) (Walder et al., 2012; Sepp et al., 2019; Davison et al., 2020). For example, Walder et al. (2012) assessed carbon investment and nutrient gains of C_3_ *Linum usitatissimum* and C_4_ *Sorghum bicolor* into and from their interconnected CMNs, respectively, formed by *Rhizophagus intraradices* or *Funneliformis mosseae* using ^13^C, ^15^N, and ^33^P as tracers. They found that the C_4_ plant invested more C in the shared CMNs and received (proportionally) less N and P in return than the C_3_ plant, which in turn benefited more from the symbiosis by investing less photosynthate and receiving the greatest share of nutrients (Walder et al., 2012).

It should be noted that disproportionate cost-benefit ratios of different plant species in the same community can be confounded by plant size, with larger individuals often receiving a larger share of limited resources and even suppressing the growth of other individuals (van der Heijden and Horton, 2009; Merrild et al., 2013; Jakobsen and Hammer, 2015). To avoid misinterpretation of the results of interactions between plants associated with AM fungi, phylogenetically close plant species with approximately the same size and growth rates are an ideal model for studying interactions between plants and the fungi as affected by the AM symbiosis in general and CMN formation in particular (Řezáčová et al., 2018b).

To date, few ecophysiological studies using up to four individuals of plants or fungi have been conducted to decipher the underlying mechanisms of C-for-P exchange between plants and the mycorrhizal networks and the interactions between coexisting plants (Nakano-Hylander and Olsson, 2007; Bever et al., 2009; Lekberg et al., 2010; Kiers et al., 2011; Walder et al., 2012; Merrild et al., 2013; Weremijewicz and Janos, 2013; Fellbaum et al., 2014; Řezáčová et al., 2018b; Ingraffia et al., 2021). One experimental approach to study the influence of mycorrhizal fungi on coexisting plant interactions and resource exchange is to impose experimental shading in order to manipulate the strength of C source sink (Kaschuk et al., 2009; Olsson et al., 2010; Konvalinková and Jansa, 2016; Lang et al., 2021). Shading duration and intensity could significantly regulate the exchange of nutrients for C and thus, the cost-benefit ratio of the symbiosis (Konvalinková et al., 2015; Zheng et al., 2015). This is of particular concern because light limitation can occur to varying degrees and at different temporal scales in different ecosystems, e.g., at the regional level due to sudden changes in weather (e.g., cloudy weather, monsoons, and thunderstorms), at the local level due to canopy cover by neighboring plants, or even at the microscopic level due to the formation of microbial biofilms on plant leaves (Konvalinková and Jansa, 2016). Experimental evidence showed that reduced investment in symbiosis by both the plant and the fungal partner in response to light limitation may result in reduced plant biomass, AM fungal root colonization rate, C allocation from the plant to the fungal partner, and P transfer from the AM fungi to the plant (Olsson et al., 2010; Fellbaum et al., 2014; Füzy et al., 2014; Shi et al., 2014; Konvalinková et al., 2015). However, it is not yet entirely clear how the reciprocal resource exchange (C in return for N and/or P) between the plant and the fungus is modulated by the reduction of assimilate supply caused by light deficiency to one or all plant partners (Weremijewicz and Janos, 2013; Konvalinková and Jansa, 2016).

Here, we aimed to understand how the presence of a single AM fungus affected plant individuals in con- and heterospecific mixtures competing for shared soil resources in their mycorrhizosphere in response to experimental shading. We tested how congeneric model grasses *Panicum bisulcatum* (C_3_) and *Panicum maximum* (C_4_) inoculated or not with the mycorrhizal fungus *Rhizophagus irregularis*, and growing in separation or in a mixture, responded to different shading treatments in terms of their biomass production, mycorrhizal colonization, and P, N, and C exchanges. For this purpose, plants were grown side by side either in a “mono system” as a pair of individuals belonging to the same plant species (C_3_-C_3_ and C_4_-C_4_) or in a “mixed system” as a pair of individuals each belonging to a different plant species (C_3_-C_4_), inoculated or not with the AM fungus. We assessed the carbon and nutrient investments of the plant and fungal partners by tracking stable and radio isotopes using ^13^C, ^15^N, and ^33^P as tracers, and by manipulating light interception by the different plants individually. The roots were allowed to intermingle freely and labeling of soil nutrients was not confined to a root-free compartment to achieve a greater relevancy to the field setting.

## 2 Methods

### 2.1 Experimental design

#### 2.1.1 Mono system

**Figure 1:**
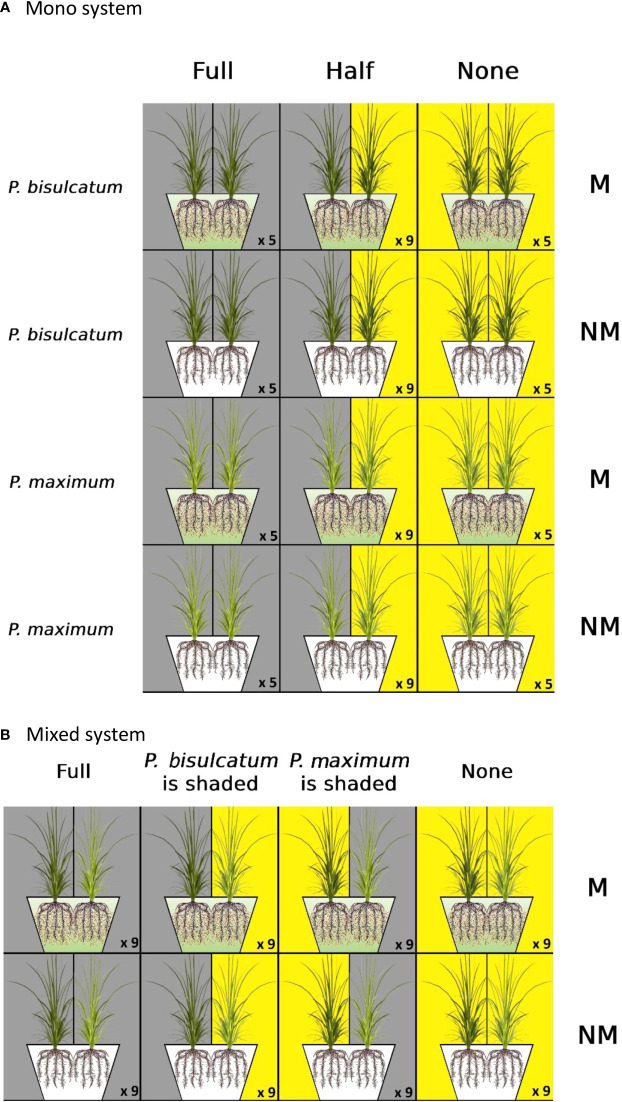
**(A)** Experimental setup in the mono system; two individuals of either plant species (*Panicum bisulcatum* or *P.maximum*) were grown in the same pot and exposed to three light regimes (“full,” in which both plant individuals in each pot were shaded, “half,” in which only one of the plant individual was exposed to shade, and “none,” in which both plants were exposed to full light). Plant individuals in each pot were either inoculated with arbuscular mycorrhizal (AM) fungus – constituting the mycorrhizal (M) treatment, whereas the AM fungus was absent in the non-mycorrhizal (NM) treatment. **(B)** Experimental setup for the mixed system; plant species (one individual of *P.bisulcatum* and one of *P.maximum*) with two mycorrhizal statuses (M/NM) were grown side by side as pairs of different plant species in the same pot, and exposed to four light regimes (“full”, in which both plant individuals in each pot were shaded, “*P.bisulcatum* is shaded”, in which only *P.bisulcatum* was exposed to shade, “*P.maximum* is shaded”, in which only *P.maximum* was shaded, and “none”, in which no plant was shaded). The number of replicates is indicated for each treatment combination.

The mono system was laid out in a fully factorial design with two *Panicum* species, C_3_ *P.bisulcatum* and C_4_ *P.maximum*, and with two mycorrhizal statuses (mycorrhizal “M” or non-mycorrhizal “NM”). Two plant individuals per pot were grown side by side inoculated with the AM fungus (or not) and exposed to three light regimes: “full,” in which both plants in each pot were shaded, “half,” in which only one of the plant individuals in each pot was shaded, and “none,” in which none of the plant individuals in each pot was shaded (**Figure 1A**). Five pots for the “full” and “none” treatments and nine pots for the “half” treatment were set up for each combination of mycorrhizal inoculation and plant species, for a total of 76 pots.

#### 2.1.2 Mixed system

The mixed system was laid out in a fully factorial design with two factors: two mycorrhizal statuses (“M” or “NM”) and four light regimes *(*“full” and “none” as in mono system, “*P.bisulcatum* is shaded”, in which only *P.bisulcatum* was exposed to shade, and “*P.maximum* is shaded”, in which only *P.maximum* was shaded). Each pot was planted with a mixture of two plant species (one individual of *P.maximum* and one individual of *P.bisulcatum*) (**Figure 1B**). Nine replicates were set up for each of the mycorrhizal inoculation treatments under each of the four light regimes, resulting in a total of 72 pots.

### 2.2 Biological materials and plant cultivation

The grass species utilized in this study were congeneric *P. bisulcatum* Thunb. and *P. maximum* Jacq., both belonging to the genus *Panicum*. The photosynthetic types of these two species are well defined, with *P. bisulcatum* being a typical C_3_ plant and *P. maximum* having a C_4_ (PCK subtype) photosynthetic metabolism (Pinto et al., 2014). The seeds were kindly provided by dr. Oula Ghannoum from Western Sydney University, Australia.

Experimental pots (11 x 11 x 20 cm, w x d x h) were filled as follows: First, 1.2 liters of sterile potting mix was added to the bottom of each pot, containing previously sterilized (gamma irradiated, >25 kGy) 10% soil from Litoměřice, Czech Republic [more details on this soil have been published previously, (Řezáčová et al., 2017)], 45% granular zeolite, and 45% sand. This substrate and its physicochemical properties have been described previously (Püschel et al., 2017). Thereafter, the pots were filled with 500 ml of either mycorrhizal (M) or non-mycorrhizal (NM) potting mix. The NM potting mix was prepared by adding 1% (v:v) of the NM mock inoculum composed of the substrate and finely chopped (<1 cm) roots and microorganisms from a previous open pot culture with leek (*Allium porrum*), cultured in the glasshouse for more than 2 years, into the sterile potting mix (see above). This procedure, aiming at introduction of the same suite of microorganisms as in the mycorrhizal treatment, but without AM fungus, into the NM treatment has been described previously (Bukovská et al., 2018; Gryndler et al., 2018). The mycorrhizal potting mix was prepared by adding 10% (v:v) of open-pot produced mycorrhizal inoculum of *Rhizophagus irregularis* BEG 158 to the NM potting mix (already containing the NM mock inoculum), as described previously (Bukovská et al., 2018). Finally, the pots were filled with 250 ml of sterile potting mix (thereafter referred to as “soil”) on the top.

**Figure 2:**
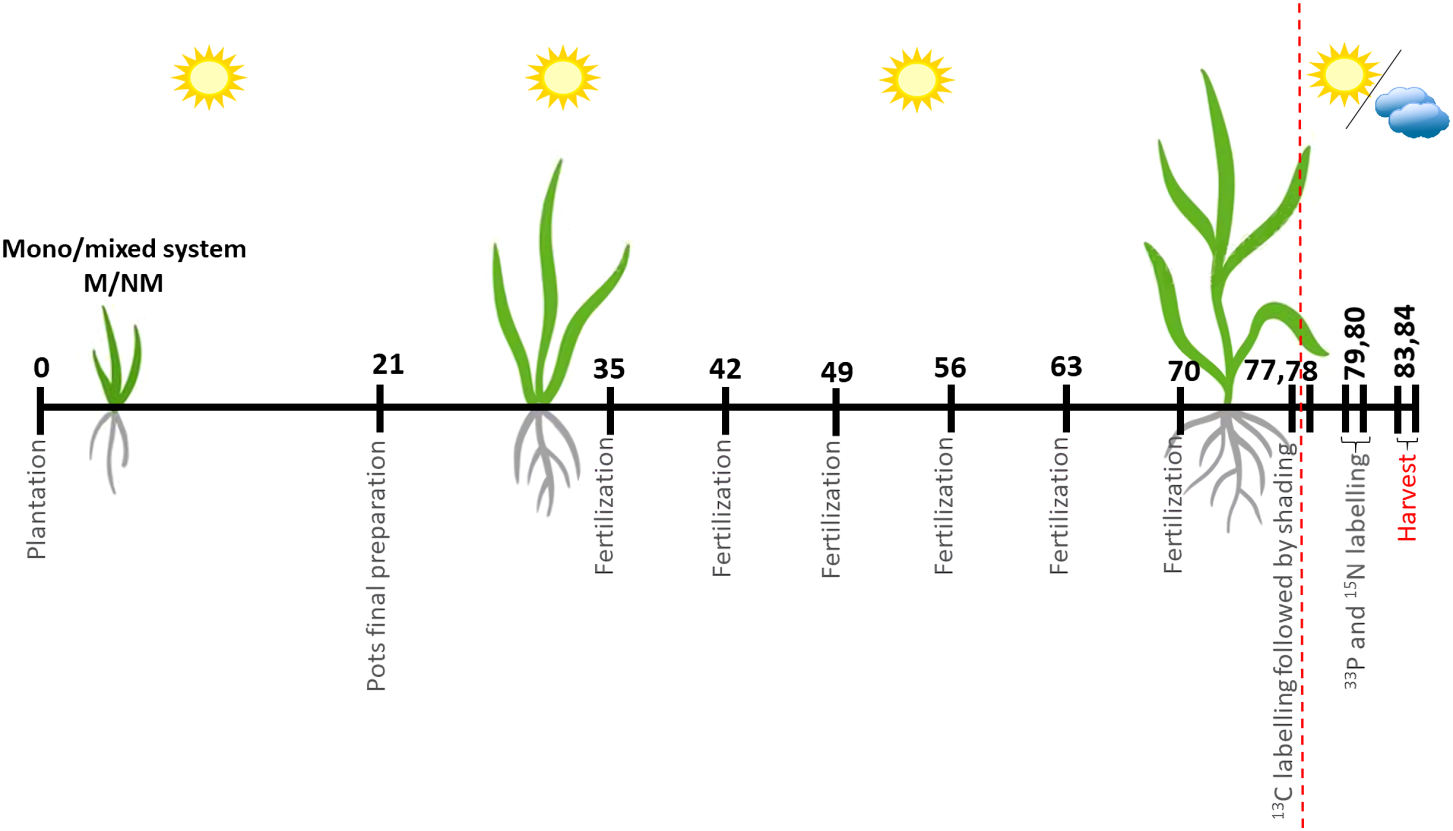
Timeline of the experiment. Final preparation of the pots was performed by plants thinned out to two individuals per pot (two individuals of the same species or one individual per pot of each plant species). Fertilization was carried out weekly by adding 65 ml of the Long-Ashton nutrient solution (containing only 20% of the original phosphorus concentration) per pot, until the ^13^C labelling. ^13^C and ^33^P+^15^N labelling was conducted on 77/78 and 79/80 days since plantation, respectively. Shading treatments were applied from day 77/78 onwards (the shading was initiated just after labeling the plants with ^13^CO_2_). Intervals not exactly to scale.

Seeds were pregerminated on moist filter paper for a total of 8 days (*P. maximum* was incubated at 37°C in the dark for two days, followed by 6 days at 25°C, *P. bisulcatum* for 8 days at 25°C). Seedlings were then transferred to pots, with one *P. bisulcatum* seedling placed in one half, and three *P. maximum* seedlings planted in the other half of the pot surface in the mixed systems. In the mono systems, two *P. bisulcatum* seedling or six *P. maximum* seedlings were planted into each pot, distributed equally between the different halves of the pot surface. During the following week from planting the seedlings into the pots (7 days), the seedlings were checked regularly, the substrate was moistened daily with the water nebulizer, and seedlings that did not survive were replaced with fresh seedlings if needed. On day 21 after planting, seedlings were thinned to always two seedlings per pot located each in one half of the pot surface (**Figure 2**), belonging either to the same or to different plant species, according to the experimental design (**Figure 1**). The positions of the pot were fully randomized in the glasshouse. From this point on, natural light was supplemented with high-pressure metal halide lamps (500W) that provided a minimum photosynthetic flux density of 200 μmol m^−2^ s^−1^ at the plant level and extended the photoperiod to 14 h. All plants were exposed to the same level of light before the isotopic labeling (see below). Deionized water was provided daily so as to maintain the substrate moisture at approximately 65% of its water holding capacity. During the subsequent weeks, the pots were re-randomized once a week and each pot received 65 ml of Long-Ashton nutrient solution with reduced P concentration (Jansa et al., 2020) per week (**Figure 2**), starting with the fertilization at 35 days after planting and the last fertilization dose added at 70 days after planting. The soil in each pot contained 195 mg of total N and 5.9 mg of plant-available (water-extractable) P per pot (Püschel et al., 2017), and the inputs with the nutrient solution were (per pot, until the isotopic labeling) 65.5 mg N, predominantly in the form of nitrate, and 3.13 mg P in the form of orthophosphate. This nutrient management resulted in P and N co-limitation of plant growth (Püschel et al., 2016).

### 2.3 Isotope labeling (^13^C, ^33^P and ^15^N) and shading

^13^CO_2_ pulse labeling was carried out six days before plant harvest (**Figure S1**) to follow the allocation of recently fixed C by individual plants into their shoots, roots, associated AM fungi, and the soil. To this end, one replicate pot from each treatment combination (i.e., plant community diversity, mycorrhizal status, and shading pattern) was left unlabeled to estimate natural isotopic abundance of ^13^C in our experimental system. Other four replicates of mono systems exposed to homogeneous light conditions (either full light or full shade) were processed as follows: one of the plant individuals per pot was tightly wrapped in aluminum foil before moving the pots under the labeling canopy (to prevent any fixation of ^13^CO_2_ by the wrapped plant during the labeling), whereas the other plant was left to photosynthesize under the labeling canopy (**Figure S1**). In the treatments with plant mixtures or with heterogeneous light conditions in mono systems (i.e., half-shaded), which encompassed 9 replicate pots each, there always were four replicates with the “left” and four with the “right” plant individual enwrapped in aluminum foil to prevent their photosynthesis during ^13^CO_2_ labeling (**Figures S2, S3**). Plants were enwrapped shortly before moving the pots under the labeling canopy, and the foil removed just after the ^13^CO_2_ labeling, thus this manipulation did not last longer than 3 hours for any single plant.

Given the number of pots and manipulations with the plants (and size limitations of the labeling canopy), the ^13^C labeling was carried out during 2 subsequent days (77 and 78 days after planting), within 5 labeling series, each involving 25 or 26 pots. After enwrapping the relevant plants in aluminum foil (see above), the pots were placed under plexiglass canopy (footprint 1 m^2^, volume 0.75 m^3^, for more details see Slavíková et al. (2017)), equipped with internal fan, and with light, CO_2_ and temperature sensors, and provided with a beaker containing 0.1 g sodium bicarbonate (99% ^13^C enrichment, Cambridge Isotope Laboratories, Tewksbury MA, USA) per each included pot. The canopy was lit from outside with two high pressure metal halide lamps (500 W each) providing a minimum photosynthetic light flux density of 300 µmol m^-2^ s^-1^ at the plant level. After closing the labeling canopy, plants were left to utilize atmospheric (unlabeled) CO_2_ for 30 min. Thereafter, ^13^CO_2_ pulse was released from the bicarbonate by adding to it excess 20% phosphoric acid. The plants were then exposed to the ^13^CO_2_ atmosphere for 90 min. After this time elapsed, the canopy was opened, the aluminum foil covers removed from the plants, and shading treatments initiated immediately as per the experimental design (**Figure 1**). To shade the plants, shading tents were constructed from green shading cloth that absorbed/reflected 65% of incoming light from above and from the sides, and transmitting 35% of the incoming light (Konvalinková et al., 2015). Depending on the treatment, whole pots were placed into the tents (i.e., fully shaded pots), or individual plants were shaded whereas the other plant in the same pot was exposed to full light (i.e., half-shaded pots). Alternatively, the pots were placed outsides of the shading tents for the non-shaded treatments (see **Figure S3** for photos).

Exactly at 47 h from starting the ^13^C labeling for each of the 5 labeling series (i.e., 79-80 days after planting the pots), 5 ml aqueous solution containing KH_2_PO_4_ (2.34 mg, i.e., 0.53 mg P, labeled with carrier-free ^33^P-orthophosphoric acid, 207 kBq, Lacomed, Kralupy nad Vltavou, Czech Republic) and ^15^NH_4_Cl (0.78 mmol N, 99+ atom% ^15^N, Cambridge Isotope Laboratories) were added to each of the pots by injection into 4 corners of each pot, 4-6 cm below surface. The amounts of P and N added with this labeling dose corresponded to the weekly dose of those nutrients added with regular nutrient solution. No other fertilizers were added at this timepoint. All pots, including those not previously labeled with ^13^C, were subject to injection of the ^33^P and ^15^N isotopes. Immediately after the labeling, 65 ml deionized water was added to each pot to facilitate diffusion of the nutrients throughout the pot volume. After the labeling with ^33^P and ^15^N, watering of the pots was adjusted/reduced according to light exposure to prevent any liquid leaching from the pots.

### 2.4 Sample collection and plant, fungal, and substrate analysis

Exactly 6 days after initiating the ^13^C labeling (i.e., days 83 and 84 after planting, **Figure 2**), each of the labeling series (and the corresponding ^13^C-unlabeled pots) were harvested in the same staggered pattern as the one employed during the isotopic labeling. Specifically, shoots of the two plants per pot were harvested (i.e., cut at the soil level) separately, retaining information of their ^13^C labeling and shading history. Thereafter, both the root and soil samples from each pot were processed as single units because it was not possible to separate the two different root systems that intermingled in each pot. Roots were shaken off the soil, which was then collected as the soil sample, and subsequently washed free of any remaining soil particles under tap water. All samples (shoots, roots, and representative soil samples devoid of roots) were placed in paper bags and moved into forced-ventilation oven (65°C) and dried for 3 days. Additionally, a subsample of each of the soils was frozen at -20°C at the time of sampling. After drying, dry weights of all shoot and root samples were recorded. Representative subsamples of the shoot and root samples (fragmented with scissors to pieces < 1 cm, not milled due to ^33^P radioactivity, and weighing between 0.12 and 0.49 g, i.e., representing 10-20% of the individual sample mass) were incinerated in muffle furnace at 550°C and extracted with boiling concentrated HNO_3_ as described elsewhere (Püschel et al., 2017; Slavíková et al., 2017). Radioactivity in the extracts was measured by ß-scintillation counting (within decay energy window 2-300 keV), using Perkin Elmer AB scintillation cocktail, combined with the samples in a ratio 5:1 (v:v, cocktail:extract). Background radioactivity in the acid extracts was estimated using analytical blanks (i.e., samples with no plant biomass input). Phosphorus concentration in the acid extracts was analyzed by Malachite green colorimetry (Ohno and Zibilske, 1991).

One year after the ^33^P labeling (when the radioactivity of all samples dropped under any detectable level), remaining samples (i.e., shoots, roots, and soils) were pulverized in a ball mill MM200 (Retsch, Haan, Germany). Thereafter, the concentrations of C and N, as well as isotopic composition of these two elements, were measured using elemental analyzer Flash 2000 coupled with isotope ratio mass spectrometer (Delta V Advantage, all instruments from ThermoFisher Scientific, Bremen, Germany). For the CN analyses, 2 mg of plant biomass and 20 mg of the soils were used, wrapped in tin capsules.

DNA was extracted from ~10 mg powdered root samples using the glassmilk method (Gryndler et al., 2014), upon addition of 2 × 10^10^ gene copies of DNA internal standard per each sample (Thonar et al., 2012). DNA from the soil samples (~600 mg each) was extracted using the PowerSoil DNeasy kit (Qiagen, Hilden, Germany), upon addition of the same DNA internal standard as above. Subsequently, the abundance of the AM fungus and the recovery of DNA standard in all the root and soil DNA samples was measured using quantitative real-time PCR (qPCR), employing Lighcycler 480-II (Roche, Rotkreuz, Switzerland) and specific primers and TaqMan probes targeting either the nuclear large ribosomal subunit RNA gene or the mitochondrial large ribosomal subunit RNA gene of *Rhizophagus irregularis* (intra nLSU or mt5, respectively (Bukovská et al., 2021)), or the internal DNA standard (Thonar et al., 2012). The assays were calibrated using amplicons generated with the relevant PCR primers and pure AM fungal DNA (intra nLSU and mt5) or with linearized plasmid carrying the internal standard (see Thonar et al., 2012 for more details). Recoveries of internal DNA standards measured in each sample post-extraction were used to correct for unspecific DNA losses upon extraction as detailed previously (Thonar et al., 2012).

Whole-cell fatty acids (WCFA) were extracted from the previously frozen and subsequently lyophilized and pulverized soil samples (10 g each), and converted to methylesters using the previously described trimethylchlorosilane procedure (Konvalinková et al., 2017). Fatty acid profiles and isotopic composition of C in the individual compounds were analyzed using the Trace 1310 gas chromatograph (ThermoFisher Scientific) coupled with the mass spectrometer (see above) *via* GC Isolink. The concentrations of the AM fungal signature fatty acid C16:1ω5 in the WCFA in the different samples were determined by comparing its concentration in the lipid extracts with the concentration of the internal standard compound (free fatty acid C19:0), spiked in known amount (100 µg) into each sample before lipid extraction.

### 2.5 Calculations

The measured P and N concentrations in roots and shoots were used to calculate the P and N contents of shoots and roots, respectively, by using the previously determined dry biomass values (P content = P concentration × dry mass). The P and N contents of the plants per pot were then the sums of the P (or N) contents in all shoots per pot and the P (or N) content of the roots from the corresponding pot.

The measured radioactivity values in the acid extracts of shoot and roots were background-subtracted and decay-corrected for the same activity date/time as the ^33^P inputs. Using these values, we calculated ^33^P transfer from the substrate to each shoot and to the roots in each pot (and expressed them as % of ^33^P applied), and the ^33^P remaining in the substrate (the latter was determined by subtraction of ^33^P transfer to shoots and roots from the total ^33^P input per pot, i.e., 207 kBq).

The ^15^N transfers from the substrate to shoots and roots were calculated as ^15^N excess following the concept presented recently in Dudáš et al. (2022), using the N contents of the different plant parts, the isotopic enrichment of the different samples (as atom%, while taking the value AT%=0.36 as the background for ^15^N natural abundance), and knowing the amount of labeled ^15^N input (0.78 mmol). The ^15^N amount remaining in the soil was calculated from the measured N concentration and ^15^N enrichment in the soil samples, assuming that the ^15^N isotope was only distributed within 600 g layer of the soil. The unaccounted ^15^N excess per pot was assumed to represent unspecific ^15^N losses.

The ^13^C partitioning within each pot was calculated using the measured C concentrations (and calculated C contents) of the different plant and soil samples, isotopic composition of C in those samples, and using ^13^C-unlabeled pots to measure natural abundance of ^13^C in the different samples. Since all ^13^C beyond the natural abundance level (i.e. ^13^C excess) must have originated from the single ^13^C-labeled plant per pot, here we could unequivocally assign the proportions of overall ^13^C budget (on a per pot basis) that remained in the shoots of the labeled plant, in the roots of the same plant, and that in the soil/AM fungal biomass to the source. This did not require equal levels of ^13^C isotopic labeling of each plant (which would be difficult to achieve). On the other hand, we could not assess respiration losses of ^13^C from the pots post-labeling. In a similar way as for the total ^13^C budget, and following the approach described elsewhere (Konvalinková et al., 2017), we calculated ^13^C allocation into the AM fungal signature fatty acid. This was expressed as a percentage of the total ^13^C fixed and recovered in each of the pots, assuming the total soil (dry) weight was 2 kg per pot and assuming homogeneous distribution of AM fungal hyphae and recently allocated ^13^C within the entire volume of the pots.

**Figure 3:**
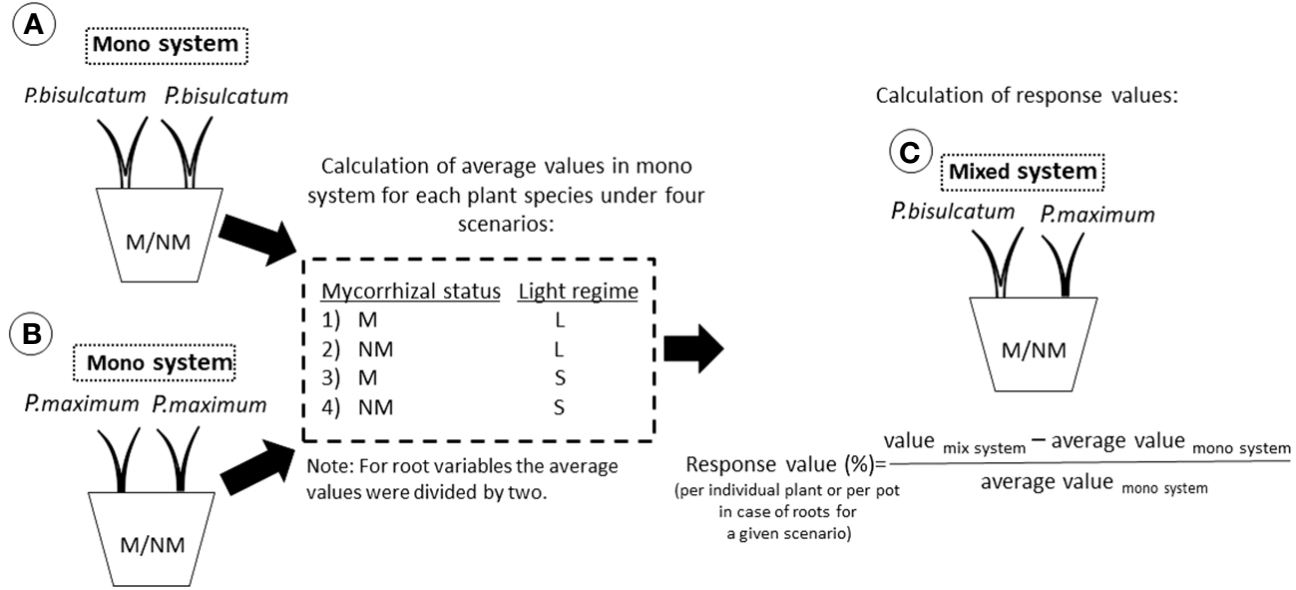
Schematic representation of the plant response model used to quantify the responses of two plant species in mixed culture compared to monoculture. **(A)** refers to a situation where two *P.bisulcatum* plants are grown together in a mono system. **(B)** refers to a situation in which two *P.maximum* plants are grown together in a mono system. **(C)** refers to a situation in which *P.bisulcatum* and *P.maximum* are grown together in mixed system. The average values for the different plants, measured in the relevant mono system, were calculated for four different scenarios: “ML”, “NML”, “MS”, and “NMS”, taking values for individual shoots and half of values for roots as data for a single plant in mono system pots. “M” and “NM” refer to mycorrhizal and non-mycorrhizal status, respectively. “L” and “S” refer to a condition where the co-cultivated plants are in full light and full shade, respectively. Response values per individual plant for a given scenario were then calculated to express percent change in the mixed system compared to the relevant mono system.

To quantify the responses of the plant species in mixed systems compared to the respective mono systems, we created a plant response model based on four different scenarios: “ML” where the M plants were under full light, “NML” where the NM plants were under full light, “MS” where the M plants were under full shade, and “NMS” where the NM plants were under full shade. We calculated the response values of each plant species for a given scenario by quantifying the percent change in the mixed system compared to mono system (**Figure 3**). By using this framework, and taking into account the homogeneously lit mono systems planted either with *P. bisulcatum* or *P. maximum*, for both mycorrhizal and NM scenarios, we could predict individual shoot and root biomass, nutrient and carbon contents, and isotopic recoveries for the different mixed systems, assuming absence of any interaction. Comparing the values measured in individual pots with those predicted from the mono systems (i.e., variables referring to individual plant shoot, and the sums of root variables), we calculated responses values as detailed in **Figure 3**.

### 2.6 Statistical analysis

Three-way and two-way ANOVAs were performed to determine the effects of different light regimes, mycorrhizal inoculation, and plant species on plant biomass, C, ^13^C, N, ^15^N, P, and ^33^P in shoot, root and soil in mono systems. In the mixed system, with exception of ^13^C isotopic data and all shoot data, which could unequivocally be tied to the individual source (labeled) plant, the belowground data (referring to roots and soils) could only be analyzed as mixes on a per-pot basis. The data describing the levels of mycorrhizal colonization of roots and soil (WCFA or qPCR analyses) were only analyzed for the M treatment, leaving out the NM from analysis (since the latter usually returned values below detection limit of the respective methods). Before calculating the ANOVA, normality and homogeneity of variance were tested to verify that the required assumptions were met. The normality assumption was tested using the residuals of the ANOVA model and the QQ plot, as well as calculating the Shapiro-Wilk test for each group level. Homogeneity of variance was tested by plotting the residuals against the fitted values and Levene’s test following Crawley (2012). In case of a significant difference between the variances of the different groups, such data heteroscedasticity was corrected by a White adjustment in ANOVA function, which provides heteroscedasticity correction using a coefficient covariance matrix (White and Macdonald, 1980). *Post hoc* multiple pairwise comparisons between groups were performed using the estimated marginal means and p-values were adjusted using Bonferroni correction.

All data analyzes were performed using R, version 4.1.0 (R Core Team, 2021). Analysis of variance and pairwise comparisons were performed in “rstatix” and “emmeans” packages, respectively. Heteroscedasticity correction was performed in case of significant differences between groups using white.adjust= TRUE. The graphs were created using “ggplot2” and “ggpubr” packages.

## 3 Results

### 3.1 Mono system

#### 3.1.1 Plant biomass

The three-way ANOVAs revealed significant effects of plant species, mycorrhizal inoculation, and shading on shoot, root and total biomass (shoot plus root) in mono system (**Table 1**). These effects were independent of one another, as no significant two- or three-way interactions (disregarding a single exception for total plant biomass close to p = 0.05) were found between the explanatory variables. In all shading and mycorrhizal treatments, shoot, root, and total dry weights were significantly higher in *P.maximum* than in *P.bisulcatum* (**Figure S4**). Moreover, M plants generally had higher root biomass than NM plants, while higher shoot and total biomass were observed in the NM plants (**Figure S4**). Plant biomass production (shoot, root and total biomass) was also consistently suppressed by shading (**Figure S4**).

**Table 1:** Results of three-way ANOVA of the effects of light regime, mycorrhizal status, and plant species in mono system.

	Plant species	Inoculum	Light regime	Plant species × Inoculum	Plant species × Light regime	Inoculum × Light regime	Plant species × Inoculum × Light regime
Shoot dry biomass	422.77 **(0.000)**	48.65 **(0.000)**	73.11 **(0.000)**	3.04 (0.086)	0.50 (0.609)	0.32 (0.730)	0.35 (0.708)
Root dry biomass	307.72 **(0.000)**	11.54 **(0.001)**	13.65 **(0.000)**	1.214 (0.275)	0.02 (0.982)	0.01 (0.543)	0.45 (0.638)
Total biomass	690.51 **(0.000)**	15.63 **(0.000)**	80.49 **(0.000)**	4.10 **(0.047)**	0.35 (0.709)	0.11 (0.893)	0.22 (0.804)
Biomass partitioning	4.89 **(0.031)**	45.22 **(0.000)**	2.59 (0.082)	1.52 (0.222)	0.42 (0.659)	0.27 (0.762)	0.34 (0.713)
Total P	49.23 **(0.000)**	16.97 **(0.000)**	5.64 **(0.006)**	0.78 (0.380)	10.81 **(0.000)**	0.47 (0.626)	2.55 (0.086)
P partitioning	23.75 **(0.000)**	23.55 **(0.000)**	22.44 **(0.000)**	0.74 (0.394)	6.68 **(0.002)**	2.35 (0.104)	0.17 (0.846)
Total ^33^P	44.6 **(0.000)**	1.88 (0.175)	1.12 (0.332)	1.59 (0.212)	6.26 **(0.003)**	6.13 **(0.004)**	2.38 (0.101)
^33^P partitioning	18.70 **(0.000)**	35.47 **(0.000)**	12.40 **(0.000)**	13.53 **(0.000)**	2.24 (0.115)	7.49 **(0.001)**	2.18 (0.122)
Total N	69.82 **(0.000)**	0.52 (0.471)	4.90 **(0.010)**	3.23 (0.077)	0.42 (0.657)	0.74 (0.482)	0.25 (0.782)
N partitioning	41.50 (0.197)	24.42 **(0.000)**	27.45 **(0.001)**	1.46 (0.295)	14.66 (0.661)	1.47 (0.848)	1.98 (0.956)
Total ^15^N	19.18 **(0.000)**	0.77 (0.383)	8.84 **(0.000)**	0.39 (0.533)	2.44 (0.095)	1.02 (0.366)	0.49 (0.612)
^15^N partitioning	44.64 **(0.000)**	26.88 **(0.000)**	59.57 **(0.000)**	1.29 (0.259)	18.01 **(0.000)**	2.02 (0.141)	1.56 (0.219)

Light regime: three levels including full, half-pot and none shading, mycorrhizal status: two levels (mycorrhizal and non-mycorrhizal), Plant species: P.maximum and P.bisulcatum. “Total” refers to the amount in shoot plus root and “partitioning” refers to the ratio of amounts in shoot to the amounts roots. F and p-values (the latter in brackets) are indicated. Significant p-values (≤ 0.05) are indicated in bold.

Significant effects of plant species and mycorrhizal inoculation (but not shading) on biomass partitioning (i.e., shoots:root biomass ratio, calculated on a per pot basis) were found, with no significant interactions between factors (**Table 1**). *P.maximum* showed higher values of biomass partitioning between shoot to root than *P.bisulcatum* (**Figure S4**). Mycorrhizal inoculation reduced the values of biomass partitioning (**Figure S4**).

#### 3.1.2 P and ^33^P uptake

Total P content of the plants and P partitioning (i.e., ratio of P content in shoots to P content in roots) were significantly influenced by plant species, mycorrhizal status and light regime in the mono system. The significant interaction between plant species and light regime was also observed for both variables (**Table 1**).

Regardless of mycorrhizal status, *P.maximum* showed significantly higher total P content and shoot to root P content partitioning than *P.bisulcatum* in the “half” and “none” shading treatments, whereas the values were not different between the plant species under full shade (**Figure S5**). Total P content and P partitioning in *P.bisulcatum* significantly increased along the shading gradient (**Figure S5**). In addition, the NM pots generally showed significantly higher P partitioning values than the M pots. The plants in M pots had significantly higher total P content than those in the NM pots (**Figure S5**).

The total ^33^P uptake by plants was significantly influenced by plant species, plant species-light regime and light regime-inoculum interactions (**Table 1**). Higher amounts of total ^33^P were detected in *P.bisulcatum* than in *P. maximum* (**Figure S5**). Further, total ^33^P significantly increased in *P.bisulcatum* and significantly decreased in *P.maximum* along the shading gradient (**Figure S5**). Mycorrhizal plants (regardless of the species) showed a significant decrease in ^33^P uptake along the shading gradient, whereas the NM plants did not show a particular trend with shading, yielding significant interaction between inoculation and light regime (**Table 1** and **Figure S5**).

We also found significant effects of plant species (*P.maximum* > *P.bisulcatum*), inoculum (NM > M), and shading (increasing with shading intensity) on *^33^* P partitioning (i.e., *^33^* P shoot to root ratio). These effects were not fully independent of each other, as the interactions between plant species and mycorrhizal inoculation and species and light regime were significant (**Table 1**). Specifically, *^33^* P partitioning increased more with shading in NM plants than in M plants. Significantly higher values of ^33^P partitioning were observed in mycorrhizal *P.maximum* than in mycorrhizal *P.bisulcatum*, whereas such a contrast was not detectable in the NM plants (**Figure S5**).

#### 3.1.3 N and ^15^N uptake

The results of ANOVA showed significant effects of plant species (*P.maximum* > *P.bisulcatum)* and shading (increasing from full light to full shade) on total N content of the plants with no significant interaction between any of the factors (**Table 1** and **Figure S6**). N partitioning (i.e., the ratio of N content in shoots to N content in roots) was significantly affected by inoculum and light regime (**Table 1**). Specifically, the NM plants showed significantly higher N partitioning values than the M plants and the values decreased gradually with shading intensity (**Figure S6**).

Total ^15^N transfer from the labeling pulse to the plants (considering both shoots and roots) was significantly affected by plant species (*P.bisulcatum* > *P.maximum*) and decreased significantly with shading (**Table 1** and **Figure S6**). Significant effects of plant species (*P.maximum* > *P.bisulcatum*), inoculum (NM > M), and light regime (increase with shading) on shoot to root partitioning of ^15^N were detected, with significant interaction between plant species and light regime (**Table 1** and **Figure S6**). Further, the increase in ^15^N partitioning between shoots and roots was more pronounced towards full shade in *P.maximum* than it was in *P.bisulcatum* (**Figure S6**).

#### 3.1.4 ^13^C fixation and allocation

No significant effects of light regime, mycorrhizal status, and plant species were observed on the total amount of ^13^C fixed by experimental plants, ^13^C allocation between shoots and roots, and ^13^C allocation ratio above/belowground (excess ^13^C in shoot/excess ^13^C in roots plus excess ^13^C in soil). However, we found a significant effect of plant species on ^13^C excess in C16:1ω5 WCFA and the fraction of excess ^13^C allocated to C16:1ω5 (**Table S1**), when considering only the M pots. Mycorrhizal *P.bisulcatum* allocated larger fraction of its carbon to the AM fungus than did the mycorrhizal *P.maximum* (**Figure S7**).

#### 3.1.5 Mycorrhizal abundance

Absolute abundances of *R.irregularis* per unit weight of roots (quantified by qPCR) and soil (quantified either by qPCR or WCFA analyses) were significantly affected by plant species in the mycorrhizal mono system (**Table S2**), with mycorrhizal abundance values being much larger in *P.bisulcatum* than in *P.maximum* (**Figure S6**). Further, there also was effect of shading (decrease with intensity of shading) on the AM fungal abundance in both roots and soil when assessed by qPCR, but not when the signature C16:1ω5 fatty acid was used as a proxy (**Table S2** and **Figure S8**).

### 3.2 Mixed system compared to mono system

#### 3.2.1 Plant biomass responses

In our plant response model, comparing the mixed to mono systems, the two experimental plant species differed significantly (p < 0.001) in terms of shoot dry biomass, with *P.bisulcatum* showing a significantly greater biomass response values than *P.maximum*, and particularly when *P.maximum* or both plants were shaded (**Table S4** and **Figure S10**). Light conditions alone and mycorrhizal status had no significant effects on the shoot biomass responses (**Table S4**).

No significant effects of light regime and mycorrhizal inoculation were found on root biomass in the mixed system compared with the mono system – with the effect of plant species not testable as the roots of the individual plants could not be separated in any of the pots (**Table S5** and **Figure S11**).

#### 3.2.2 P and ^33^P uptake responses

The shoot P response in the mixed system compared to the mono system was significantly affected by light regime (p < 0.001), mycorrhizal status (p < 0.001), and plant species (p < 0.001), as well as all their interactions except for the interactions between plant species and mycorrhizal status (**Table S4**). Mycorrhizal *P.bisulcatum* showed significantly higher shoot P content responses than mycorrhizal *P.maximum* under all shading treatments except for the situation in which *P.maximum* was shaded (**Figure S4**).

The root P response in the mixed system compared to the mono system was significantly affected only by the interaction between light regime and mycorrhizal status (p = 0.046) (**Table S5**). The M and NM plants differed significantly when *P.maximum* was shaded (**Figure S11**), with higher root P content response in M pots as compared to NM pots. A reverse effect (NM > M) was observed when both plants were shaded in the mixed system.

**Figure 4:**
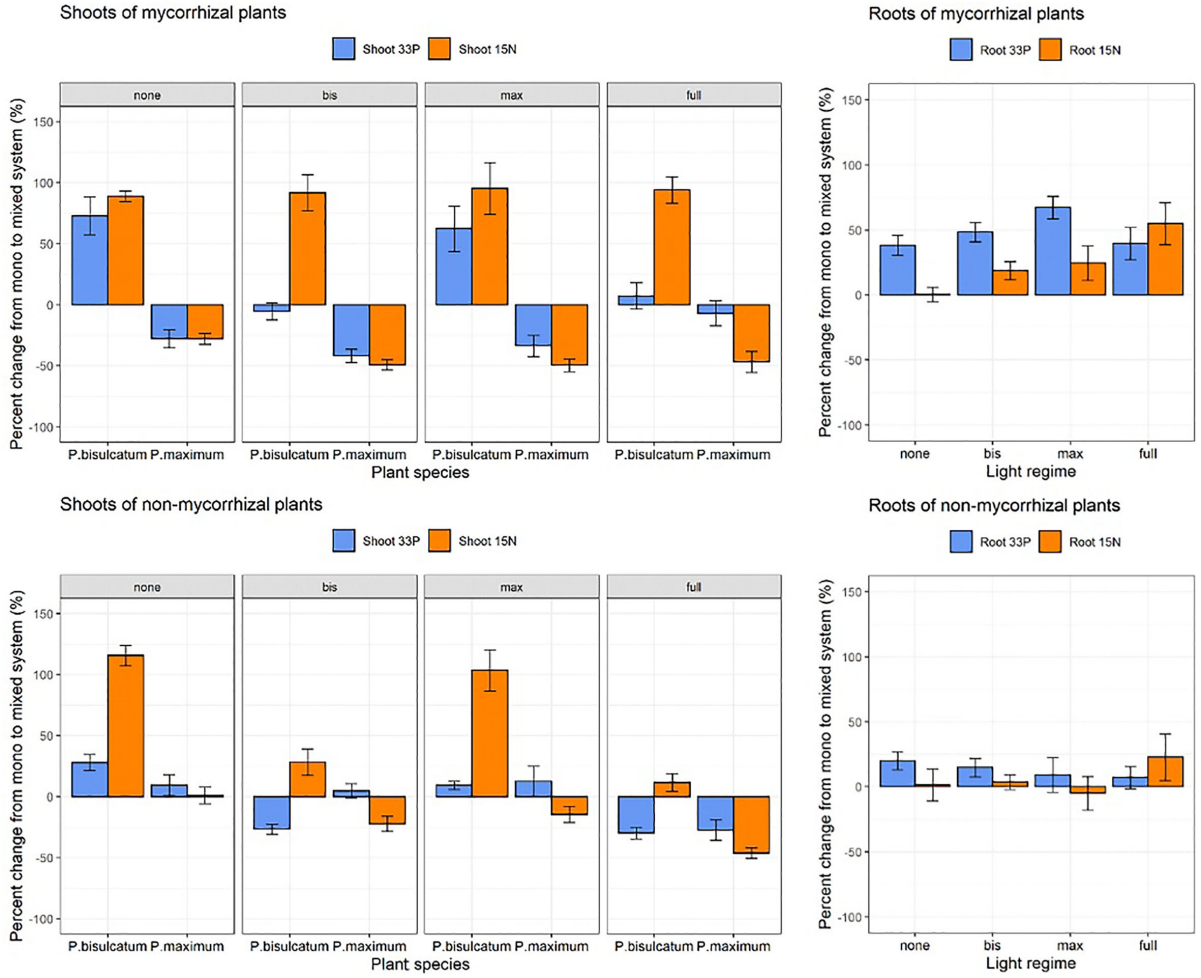
The effects of light regime, mycorrhizal status, and plant species on shoot ^33^P-, shoot ^15^N, root ^33^P and root ^15^N responses in mixed system compared to mono system. Light regime: none (no shading), full (both plants are shaded), bis (*P.bisulcatum* is shaded) and max (*P.maximum* is shaded). Mycorrhizal status: mycorrhizal (R.irregularis) and non-mycorrhizal (NM).

The shoot ^33^P response was significantly affected by light regime (p = 0.002), plant species (p = 0.039), while no significant effect of mycorrhizal status alone was found. However, all interactions between the main factors were significant (p < 0.05) in the mixed system compared with the mono system (**Figure 4** and **Table S4**). Mycorrhizal *P.bisulcatum* showed significantly higher shoot ^33^P content response than the NM *P.bisulcatum*, whereas M *P.maximum* ^33^P shoot content response was always lower than that of the nonmycorrhizal *P.maximum* plants under all shading treatments except for the situation in which both plants were shaded (**Figure S4**).

Significant effects of mycorrhizal status on root ^33^P response (p < 0.001) were found in the mixed system compared to the mono system, while light regime and interaction of light regime and mycorrhizal status were not significant (**Table S5**). A Higher ^33^P response in the roots was always observed in M pots than in NM pots compared to the mono systems (**Figure S5** and **Figure 3**).

#### 3.2.3 N and ^15^N uptake responses

The N content response (mixed vs. mono systems) of the shoots was significantly affected by all factors and their interactions (**Table S4**). Except for the light regime in which *P.maximum* was shaded alone, *P.bisulcatum* had significantly higher shoot N content response than *P.maximum* (**Figure S10**). The root N content response was not affected by any of the experimental factors and their interactions in the mixed system compared with the mono system (**Table S5**).

Shoot ^15^N response was significantly affected by all factors and their interactions in mixed system compared with mono system (**Table S4** and **Figure S10**). *P.bisulcatum* had significantly higher shoot ^15^N content response in M as compared to NM pots when it was shaded (either alone or when both plants in the pot were shaded) as compared to situation when it was not shaded – and when the contrast between M and NM pots effectively reversed (**Figure S10**). The root ^15^N response was significantly affected by light condition (p=0.018) and mycorrhizal inoculation (p=0.033), with the values in M pots being generally higher than in the NM pots and higher values detected in fully shaded pots as compared to fully lit pots (**Table S5** and **Figure 3**).

#### 3.2.4 ^13^C fixation and allocation responses

The total amount of excess ^13^C response was significantly affected by mycorrhizal inoculation and light regime and several interactions in the mixed system compared to the mono system (**Table S6**). The plant species showed no significant difference in total excess ^13^C response, but the interaction between plant species and light regime was significant (**Table S6**). The NM plants generally had a significantly higher total excess ^13^C response than the M plants (**Figure S12**). This response was higher for NM *P. maximum* as compared to NM *P. bisulcatum*, whereas the contrast largely disappeared when the plants were mycorrhizal (**Figure S12**). The response was also higher for *P.maximum* than *P.bisulcatum* upon shading the *P.bisulcatum* plant, whereas the reverse was observed when none of the plants was shaded (**Table S6** and **Figure S12**).

In the mixed system compared to the mono system, the ^13^C excess in the C16:1ω5 WCFA as well as the ^13^C allocation to C16:1ω5 were significantly affected by both plant species and the light regime (**Table S7**). Compared to the mono system, a greater ^13^C excess in WCFA and a higher ^13^C allocation to C16:1ω5 were observed in *P.bisulcatum* as compared to *P. maximum*. Generally, the lowest values were observed when no plant was shaded, and highest values were observed when only one of the plants per pot was shaded (**Figure 4** and **Figure S12**).

#### 3.2.5 Mycorrhizal abundance responses

**Figure 5:**
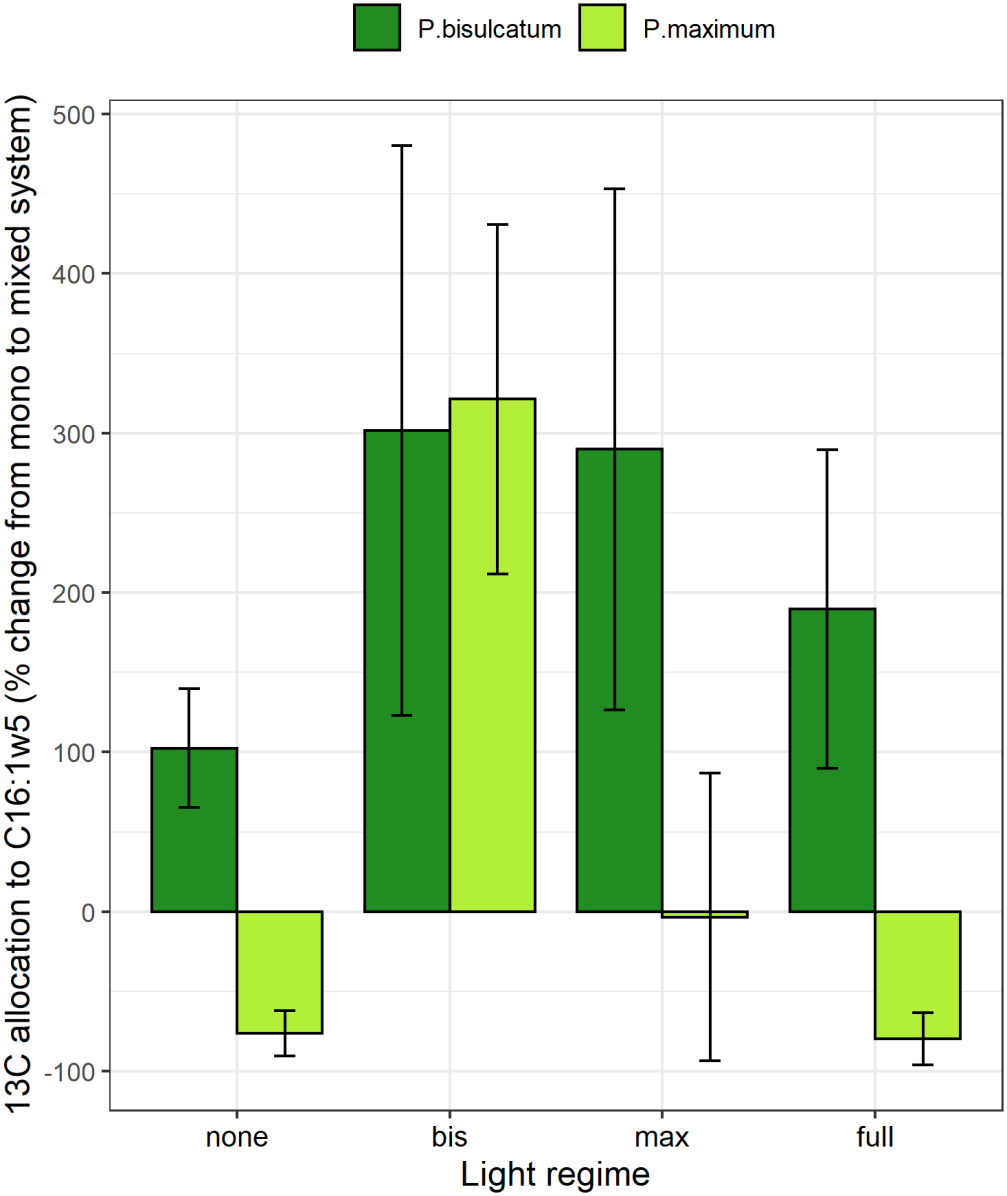
The effects of light regime and plant species (*P.bisulcatum* and *P.maximum*) on ^13^C allocation to the mycorrhiza-specific fatty acid C16:1ω5 in mycorrhizal pots in mixed system compared with mono system. Light regime: none (no shading), full (both plants are shaded), bis (*P.bisulcatum* is shaded) and max (*P.maximum* is shaded).

The response (mixed vs. mono systems) in abundance of AM fungi in roots of the M pots was significantly affected by the light regime (**Table S8**). Compared with mono system, mycorrhizal root colonization decreased under full light, whereas it increased under the condition when both plants were shaded in the mixed system (**Figures S13** and **Table S8**). No significant effect of the light regime was found on mycorrhizal abundance in soil measured by either qPCR or WCFA techniques, although the trends were similar as for the AM fungal abundance in roots (**Table S8**; **Figures 5**, **S13**).

## 4 Discussion

The observations made in our glasshouse experiment offer unique insights into P, N, and C fluxes between an AM fungus and two different but closely related plant species growing in a mixture under contrasting light conditions applied locally on one or both plants per pot. This experimental model is particularly suitable for testing re-arrangements of nutrient-for-C exchanges as it prevents confounding effects of plant size under the different light regimes. We constructed a model mono system consisting of a pair of individuals of either *P.bisulcatum* or *P.maximum* grasses growing in microcosms with or without mycorrhiza to set the baseline. We further studied the association between a heterospecific plant community and the AM fungus in a mixed system, accommodating those two different plant species within the same microcosm. We found that the two plant species in our study benefited differently from their associated mycorrhiza in the mono system, and that such inequalities were generally amplified and, in consequence, significantly affected resource use by the different plant species in the mixed system.

### 4.1 Mono system

Our study showed that *P.maximum*, when alone, performed somewhat better than *P.bisulcatum* in terms of shoot, root and total biomass production [similar to previously published results, e.g., Řezáčová et al. (2018b) and Řezáčová et al. (2017)] in both M and NM inoculation treatments and under all shading regimes. Accordingly, *P.maximum* had significantly higher total P and total N contents than *P.bisulcatum* in the mono system. In contrast, *P.bisulcatum* showed significantly higher biomass partitioning between shoot and roots compared to *P.maximum*. Interestingly, our data also showed that *P.bisulcatum* generally had higher uptake of recent nutrients (total ^33^P, total ^15^N) than *P.maximum*. These results, combined with the observations of higher ^13^C allocation to AM fungi (indicating a higher quality host) and higher mycorrhizal abundance in the roots and rhizosphere soil of *P.bisulcatum* compared to *P.maximum* in the mono system, led us to conclude that the two plant species likely differed in their dependence on the fungal symbiont, with *P.bisulcatum* exhibiting higher level of mycotrophy (or mycorrhizal dependence, particularly with respect to mineral nutrient acquisition) than *P.maximum.* The differential levels of dependence of the plant species on mycorrhizal symbiosis for nutrition suggests that the benefits and costs derived from the symbiotic association may differ among the two plant species, consistent with previous research (van der Heijden and Horton, 2009; Bever et al., 2010; Hempel et al., 2013; Jakobsen and Hammer, 2015), even in the case of phylogenetically such closely related (albeit physiologically quite different) plant species as studied here.

**Figure 6:**
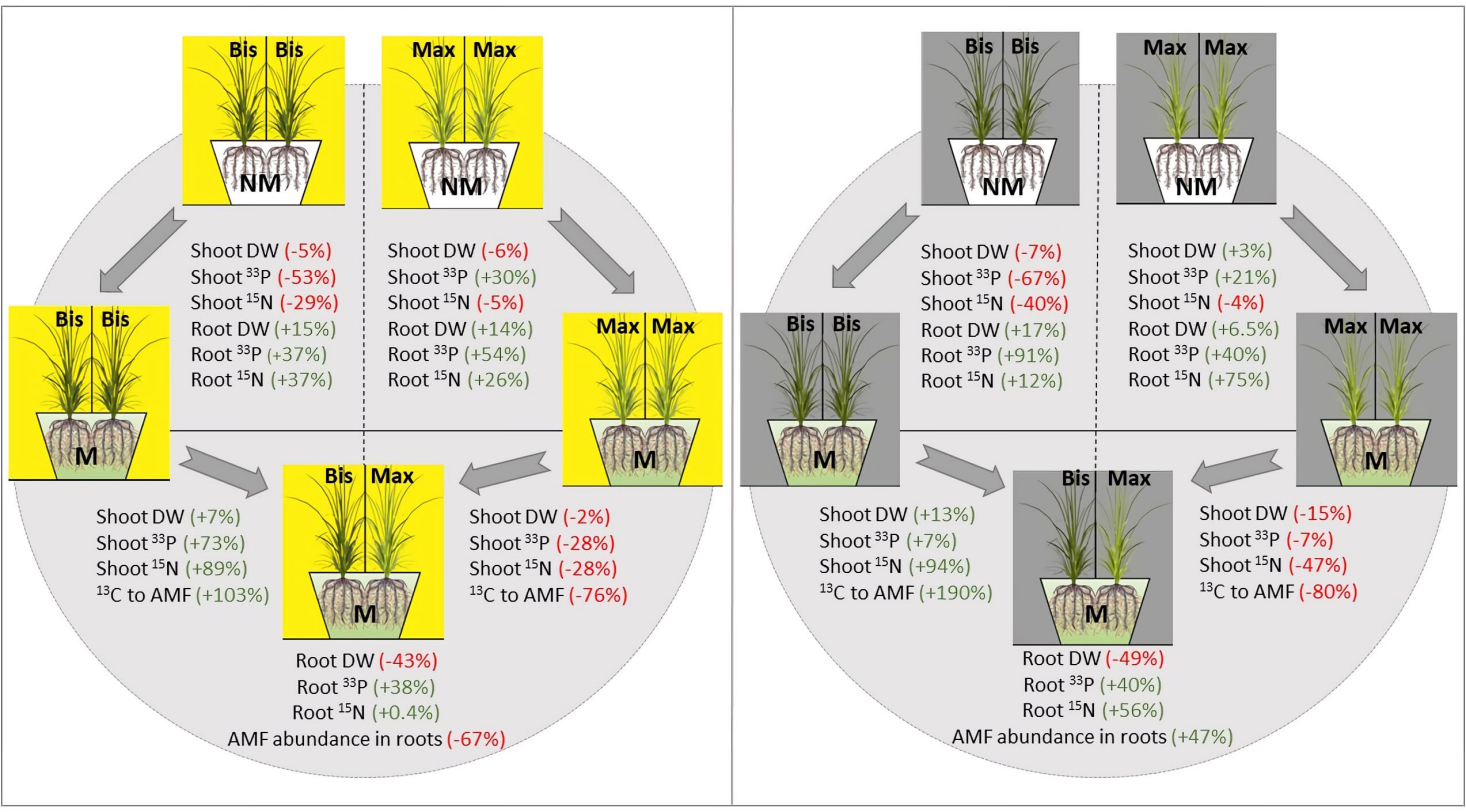
Summarization of changes in shoot and root dry biomass, ^33^P and, ^15^N resource uptake into above- and belowground plant tissues in mycorrhizal and non-mycorrhizal *P.bisulcatum* (Bis) and *P.maximum* (Max) as well as ^13^C allocation to AM fungi and abundance of mycorrhizal fungi in roots under full light and full shade regimes, from mono- to mixed-systems. “M” and “NM” refer to mycorrhizal and non-mycorrhizal status, respectively. Arrows indicate changes from one condition to another conditions. The values calculated for the transition from mono- to mixed systems are based on a theoretical model prediction, using the mono system as a baseline, and calculated response values from mono- to mixed-systems. AM fungal abundance in the roots is based on qPCR quantification of the AM fungal DNA in roots, whereas the ^13^C allocation to AM fungi is based on the fatty acid analyses in soil.

The general perception is that mycorrhizal fungi discriminate between host plants that are interconnected *via* CMN and preferentially allocate more mineral nutrients to high-quality (i.e., more C rewarding) hosts (Hammer et al., 2011; Kiers et al., 2011; Fellbaum et al., 2014). This may also be true from a phytocentric perspective: The more the plant depends on mycorrhiza for nutrient uptake, the more likely it is to provide ample C resources to the mycorrhizal network (Jakobsen and Hammer, 2015). However, in our mono system, the more mycorrhiza-dependent plant, *P.bisulcatum*, in spite of providing more ^13^C to its fungal symbiont actually received lower ^33^P benefits than the plant with the lower mycorrhizal abundance (and dependence), *P. maximum* (**Figure 6**), at least with respect to the ^33^P in the leaves. In this case, the fungus probably derives more benefit from symbiosis with the mycorrhiza-dependent *P. bisulcatum* in plant-fungal association in the mono system. In contrast, in the monoculture of *P.maximum*, the fungus, as an obligate biotroph, only had access to a low C rewarding (low quality) host and had no choice but providing nutrients to such a host, even when the quality of the host further decreased by shading. In the latter case, it is probably the host plant that benefits more from the symbiosis. Thus, in agreement with Fellbaum et al. (2014), we demonstrated that in the absence of choice for the fungus, the cost to benefit ratio of the mycorrhizal symbiosis shifts in favor of the less photosynthate-rewarding hosts (**Figure 6**).

Plant dependence on mycorrhiza can also be altered by changes in environmental conditions such as light intensity (Konvalinková and Jansa, 2016). Experimental shading has been shown to significantly affect C allocation to mycorrhiza and C-P interactions by altering the plant photosynthetic rates (Kaschuk et al., 2009; Konvalinková et al., 2015; Zheng et al., 2015; Lang et al., 2021). Our results showed that in mycorrhizal *P.maximum*, ^33^P partitioning between shoot and roots significantly increased, and total ^33^P significantly decreased by shading in the mono system. Conversely, no significant effect of shading on ^33^P partitioning and total ^33^P uptake was observed in mycorrhizal *P.bisulcatum*. These findings suggest that the more mycorrhiza-dependent *P.bisulcatum* is likely to cope better with stress caused by changes in incoming light intensity than the less mycorrhiza-dependent (and more resources-demanding C4 species) *P.maximum*. In other words, mycorrhizal association could possibly attenuate the response of *P.bisulcatum* to the shading stress. In addition, previous studies have shown that photosynthate investment in mycorrhiza decreases as light intensity decreases, resulting in lower mycorrhizal root colonization below a certain light intensity/duration threshold (Gorzelak et al., 2015; Konvalinková et al., 2015). In fact, to maintain the resource economics, plants are thought to invest more biomass in aboveground structures and less biomass in mycorrhizal network when light intensity is low (Johnson, 2010). Accordingly, Konvalinková and Jansa (2016) have shown that plants do not deliver more C to mycorrhiza under intensive shading conditions extending over several weeks, compared to ample light conditions. However, we did not detect any changes in ^13^C allocation to mycorrhizal networks in the studied plant species along the shading gradient in the mono system. This could in fact be due to the short duration of shading. We applied a short-term shading regime here (slightly less than one week) that may not have been sufficient to observe changes in mycorrhizal colonization and/or mycorrhizal C allocation. Similarly, Konvalinková et al. (2015) found no significant change in mycorrhizal colonization of roots under short-term shading (6 days), but colonization of roots by the AM fungi was significantly reduced after long-term shading (38 days) (Konvalinková et al., 2015). Therefore, not only the intensity but also the duration of light shortage could influence the exchange of resources between plants and their associated mycorrhizal networks.

### 4.2 Mixed system

Based on the results of plant-mycorrhizal interactions in mono systems, we could speculate that the outcome of plant-plant interactions in mixed system is likely to be antagonistic, due to contrasting rates of resources possibly provided by the mycorrhizal networks, as well as due to direct root competition for (limited) soil resources that may take place between different plant species. The two plants grown in a mixture showed different biomass production compared to those grown in monocultures, with the dry weight of the generally more productive but less mycorrhiza-dependent *P.maximum* reduced in the mixed culture compared to the monoculture (**Figure 6**). In contrast, biomass production was promoted in the more mycorrhiza-dependent *P.bisulcatum* in mixed culture, suggesting that the presence of AM fungus could significantly alter plant productivity in favor of the more C rewarding host.

**Figure 7:**
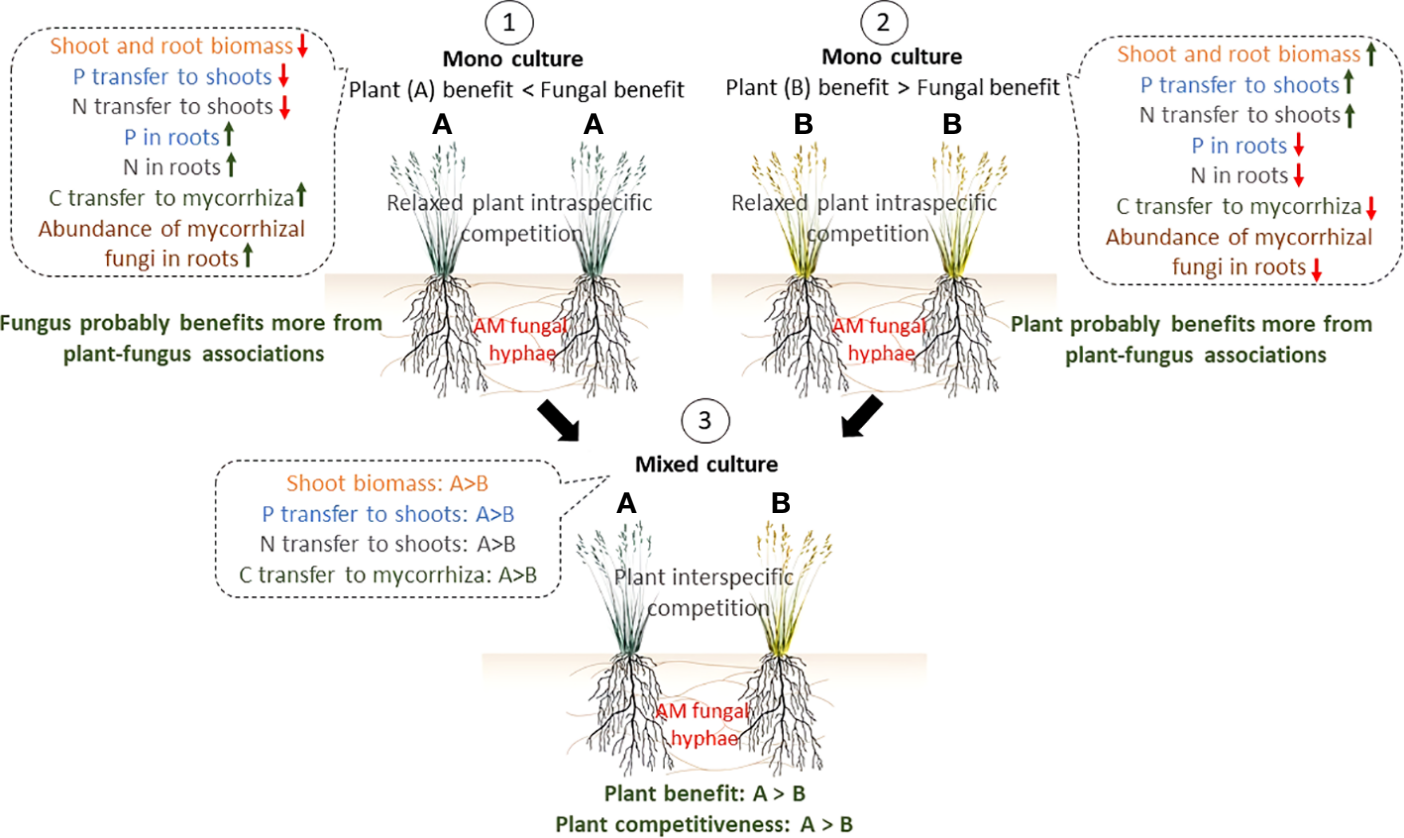
A conceptual model of interactions between a single AM fungal taxon and coexisting plants **(A, B)**, relevant to experimental results presented here. Benefits from a plant perspective were defined in terms of biomass and nutrient accumulation in aboveground plant tissues. In Scenario 1, where two individuals of highly-mycotrophic plant species A are connected *via* mycorrhizal networks, fungus receives relatively more C resources from plants and the abundance of mycorrhiza in the root/soil thus increases as compared to scenario 2. However, in return, the mycorrhiza may not equally benefit the plants by providing more N and P, compensating for the C investments. Thus, we assume that in this case the mycorrhiza benefits more than the plant from the symbiotic association. In scenario 2, where two individuals of a less-mycotrophic plant species B are interconnected *via* a mycorrhizal network (or colonized by two overlapping networks), the fungus receives less C resources from the plants compared to scenario 1, but as an obligate biotroph, has no choice but to provide nutrients to the plants. Thus, we assume that the plant is actually “in control” of the symbiosis in this case. In scenario 3, where coexisting plant species A and B are associated with the shared CMNs, more mycorrhiza-dependent (= more mycotrophic) plant A benefits more from the association with mycorrhiza compared to less mycorrhiza-dependent plant **(B)**. Plant **(A)** also receives more recent nutrients and shows higher competitiveness. Thus, AM fungi amplify inequalities among different individuals of plant species A and B by preferential rewarding of the different host plants.

In addition, in M pots under no shading in the mixed system compared with the respective mono systems, ^33^P and ^15^N increased by 73% and 89%, respectively, in the shoots of *P.bisulcatum*, whereas they both decreased by 28% in shoots of *P.maximum*. Moreover, ^33^P increased by 38% in the roots of the fully lit pots with mycorrhiza (**Figure 6**). Despite the increase in ^13^C allocation to mycorrhiza (+103%) by *P.bisulcatum*, mycorrhizal abundance in roots and soil decreased by 67% and 36%, respectively, in the unshaded pots. This can be explained by the observation that C allocation to mycorrhiza by *P.maximum* decreased by as much as 76% in in the mixed system as compared to the mono system. It appears that more mycotrophic *P.bisulcatum* acts as a better competitor for the uptake of recent nutrients than *P.maximum*, and provides more C resources for mycorrhiza, if inoculated with the AM fungus and grown together, under no shading. *P.bisulcatum* provided even more C to mycorrhiza (+190% increase in ^13^C allocation to AM fungi) when two AM-inoculated plants were shaded in the mixed system compared to the mono system, while *P.maximum* maintained the same strategy of decreasing C allocation to the mycorrhiza (-80%). *P.maximum* allocated more photosynthates to AM fungi than *P.bisulcatum* only when exposed to full light, whereas its competitor, *P.bisulcatum*, was suppressed by shading at the same time. Similar to our results, in a compartmented microcosm and using AM fungi-specific fatty acid C16:1ω5, Řezáčová et al. (2018b) found that *P.bisulcatum* preferentially fed the CMNs (consisting of five mycorrhizal fungal genera), and this contrasted to *P.maximum*, even at a high temperature, when these two plants were grown together in a mixed system, and where C_4_ photosynthesis type (*P.maximum*) would be predicted to be a more beneficial trait for plant growth/C reserve accumulation (Edwards and Still, 2008). Such differences in C inputs into the AM fungi by different plant species has also been reported in non-congeneric plant species growing in mixtures. For instance, Walder et al. (2012) found that in microcosms where two plant species were connected by the shared CMNs, the plant that invested less carbon in CMNs received relatively greater share of the nutritional (P and N) benefits from the CMNs under unshaded conditions. Thus, coexisting plant species may not benefit equally from mycorrhiza in terms of nutrient acquisition and biomass production (Walder et al., 2012; Jakobsen and Hammer, 2015; Wang et al., 2016; Řezáčová et al., 2018a). In fact, in the mixed systems, when plants competed for available resources, mycorrhizal fungi gain a “bargaining power” and are likely to transfer more nutrients to those plants that provide more C to the them (Bücking et al., 2016). This could in consequence amplify inequalities among plant species in a community by providing additional nutrients and promoting the competitiveness of more C rewarding hosts/individuals (van der Heijden and Horton, 2009; Booth and Hoeksema, 2010; Merrild et al., 2013; Weremijewicz and Janos, 2013) (**Figure 7**). On the other hand, this could potentially promote the diversity in plant communities by suppressing dominants and promoting community evenness (see below). It should be noted, however, that the imbalanced outcome of competition between mycorrhizal plant species is not always directly related to C investments into mycorrhiza. Contribution of context-dependency of plant-mycorrhizal interactions such as nutrient availability, plant species, and fungal identity, should still be quantified (van der Heijden and Horton, 2009; Gorzelak et al., 2015; Montesinos-Navarro et al., 2019). Further research is also needed to better understand the underlying the exact mechanisms of resource exchange in plant-mycorrhizal interactions, particularly when more co-occurring plant individuals are connected to the shared mycorrhizal networks.

It has also been suggested that resource sharing through mycorrhizal networks may act as a fitness balancing mechanism that minimizes fitness differences among plant species, leading to improving plant coexistence (Bever et al., 2010; Montesinos-Navarro et al., 2012; Bücking et al., 2016). This could be the case under natural conditions when multiple plant species and mycorrhizal fungi interact simultaneously in a complex network of many interactions and the symbiotic partners are hardly ever dependent on a single partner, particularly given the low host specificity in AM symbiosis (van der Heijden et al., 1998; Bücking et al., 2016). However, in our experiment, a single fungal taxon formed CMNs, which may explain why no positive or even neutral plant-plant interactions were observed in our mixed system. Admittedly, we did not measure fitness here, but biomass production could serve as a crude proxy for plant fitness (Younginger et al., 2017). Different fungal taxa demonstrably differ in providing resources to different host plants and also have different effects on plant responses to biotic and abiotic stresses (Klironomos, 2000; Montesinos-Navarro et al., 2019). In addition, conspecific individuals tend to have greater niche overlap than heterospecific plant individuals, which could lead to greater competition for available resources and suppressing fitness/growth. Overall, although simplified and artificial experimental setups with low complexity may overlook environmental heterogeneity and other potentially contributing factors, such studies as presented here, using microcosms with plants interconnected or not by the same AM fungal network, are of particular interest because they could contribute to a better understanding of the processes occurring in mycorrhizal networks, especially when multi-isotope labeling is employed (van der Heijden and Horton, 2009).

## 5 Conclusion

The two host plants in our study supported their fungal partner in different ways. We found a disproportionate allocation of C resources from different plant species to their associated AM fungi, with more ^13^C transferred from *P.bisulcatum* than from *P.maximum* to the mycorrhiza in both mono and mixed systems, suggesting that *P.bisulcatum* invests more C than *P.maximum* into the mycorrhizal symbiosis. The higher C allocation of *P.bisulcatum* to mycorrhiza suggests a high-quality (or high-intensity) interaction between *P.bisulcatum* and its fungal partner, leading to higher abundance of AM fungi in its roots and surrounding soil compared with *P.maximum.*

Our results demonstrated the advantage of more mycorrhiza-dependent *P.bisulcatum*, when grown together with less mycorrhiza-dependent *P.maximum*, particularly in terms of uptake of recent nutrients (shoot ^33^P and ^15^N) under variable light conditions in the mixed system. In addition, *P.maximum* was negatively affected by the enhanced competitive ability of *P.bisulcatum* in the presence of AM fungus. These findings suggested that the effects of AM fungal presence in mixed system were closely related to the degree of the host plant dependency on mycorrhizal association. The fungus preferentially transferred more nutrients to the more mycorrhiza-dependent plants, which in turn provided more C and enhanced its ability to thrive even under shading, on the expense of the less mycorrhiza-dependent plant. In contrast, the effects of CMNs formed by a single fungal taxon on plant nutrient uptake in a mono system are mainly exploited by the plant partner, since there is obviously no other choice for the fungus.

Overall, our results showed that the mycorrhizal symbiosis strongly affected plant species coexistence by enhancing differences in plant fitness through asymmetric resource allocation in favor of a higher quality host. On this basis, preferential allocation could enhance the success of plant species with greater mycorrhiza-dependency and/or more C provision to the AM fungus, when in plant communities. Future research is required to test the general validity of our observations (more plant and fungal species to be included) and identify the factors that may further condition asymmetries in resource exchange in plant–mycorrhizal interactions. Particularly, separation of root and AM fungal contribution should be achieved [although these two often intermingle in natural settings and not all AM fungi efficiently colonize root-free patches (Smith et al., 2004)] to improve mechanistic understanding of the systems and our capacity to predict outcomes of the competitive interactions. This could eventually lead to better predictions of the plant community spatial and temporal dynamics and mycorrhizal functioning under different and/or gradually changing environmental conditions.

## Data availability statement

The raw data supporting the conclusions of this article will be made available by the authors, without undue reservation.

## Author contributions

MF and JJ conceived and developed the study. MF drafted the first version of the manuscript. MF and JJ jointly contributed to revisions and both also approved the final version. All authors contributed to the article and approved the submitted version.
