# Supplementary material for: Mycorrhiza governs plant-plant interactions through preferential allocation of shared nutritional resources: A triple (13C, 15N and 33P) labeling study
Source: Front Plant Sci. 2022 Dec 15;13:1047270. doi: 10.3389/fpls.2022.1047270 (PMC9799978; doi:10.3389/fpls.2022.1047270)
Supplement: Supplementary file 2 [file DataSheet_1.docx]

**Supplementary information**

# Tables

**Table S1.** Results of two-way ANOVA of the effects of light regime (light vs. shadow) and plant species (*P.bisulcatum* and *P.maximum*) on ^13^C excess in C16:1ω5 fatty acid within the whole-cell fatty acid (WCFA) lipid extracts, and ^13^C allocation to C16:1ω5 in mycorrhizal mono system pots. F and p-values (the latter in brackets) are indicated. Significant p-values (≤0.05) are indicated in bold.

|  | Plant species | Light regime | Plant species × Light regime |
| --- | --- | --- | --- |
| ^13^C excess in C16:1ω5 WCFA (µmol pot^-1^) | 17.16 **(0.000)** | 0.50 (0.610) | 1.66 (0.211) |
| ^13^C allocation to C16:1ω5 (%) | 42.27 **(0.000)** | 0.10 (0.906) | 1.34 (0.282) |

**Table S2.** Results of two-way ANOVA of the effects of light regime and plant species (*P.bisulcatum* and *P.maximum*) on abundance of *Rhizophagus irregularis* in mycorrhizal mono system pots. F and p-values (the latter in brackets) are indicated. Significant p-values (≤0.05) are indicated in bold.

|  | Plant species | Light regime | Plant species × Light regime |
| --- | --- | --- | --- |
| AM fungal abundance in roots (qPCR) (LSU copies mg^-1^ root) | 88.40 **(0.000)** | 5.80 **(0.009)** | 2.36 (0.116) |
| AM fungal abundance in soil (qPCR) (LSU copies g^-1^ soil) | 58.39 **(0.000)** | 6.65 **(0.005)** | 2.41 (0.111) |
| AM fungal abundance in soil (C16:1ω5 µg C g^-1^ soil) | 13.62 **(0.001)** | 0.85 (0.439) | 2.28 (0.123) |

Note: AM fungal abundance in root and soil was quantified either by quantitative real-time PCR (qPCR) targeting nuclear large ribosomal subunit (LSU) gene of the fungus *R.irregularis* or C16:1ω5 fatty acid content in the whole-cell fatty acid fraction of substrate lipids.

**Table S3.** Results of three-way ANOVA of the effects of light regime, mycorrhizal status, and plant species on excess ^13^C total (mmol ^13^C/pot), ^13^C allocation from shoot to root and ^13^C allocation from aboveground to belowground in mono system. F and p-values (the latter in brackets) are shown.

|  | Plant species | Inoculum | Light regime | Plant species × Inoculum | Plant species × Light regime | Inoculum × Light regime | Plant species × Inoculum × Light regime |
| --- | --- | --- | --- | --- | --- | --- | --- |
| Total excess ^13^C (µmol) | 0.56 (0.460) | 0.18 (0.676) | 0.10 (0.754) | 0.89 (0.350) | 0.28 (0.602) | 2.23 (0.141) | 0.36 (0.550) |
| ^13^C allocation from shoot to root (%) | 0.14  (0.712) | 2.07 (0.157) | 2.83 (0.099) | 0.05 (0.829) | 1.44 (0.236) | 0.15 (0.700) | 3.07 (0.086) |
| ^13^C allocation from aboveground to belowground (%) | 2.64 (0.110) | 0.39 (0.534) | 0.93  (0.340) | 0.26 (0.612) | 0.02 (0.892) | 0.12 (0.728) | 2.01 (0.162) |

Excess ^13^C total: sums of ^13^C excess in all compartments of the individual treatments (excess ^13^C in plant 1+ excess^13^C in plant 2+ excess ^13^C in roots + excess ^13^C in soil); ^13^C allocation from shoot to root: excess ^13^C in labelled plant shoots/ excess ^13^C in the roots; ^13^C allocation from aboveground to belowground: ^13^C allocation in shoots/ (^3^C allocation into roots + soil)

**Table S4.** Results of three-way ANOVA of the effects of light regime, mycorrhizal status, and plant species on shoot biomass-, shoot P-, shoot N-, shoot ^33^P- and shoot ^15^N-responses in mixed system as compared to the mono system. Cation of statistical significance, ns – p ≥ 0.05, * – 0.05 > p ≥ 0.01, ** – p < 0.01.

|  | Plant species | Inoculum | Light regime | Plant species × Inoculum | Plant species × Light regime | Inoculum × Light regime | Plant species × Inoculum × Light regime |
| --- | --- | --- | --- | --- | --- | --- | --- |
| Shoot biomass | 34.14  **(0.000)** | 0.003  (0.955) | 0.236  (0.871) | 0.328  (0.568) | 2.86  **(0.04)** | 0.564  (0.640) | 1.573  (0.199) |
| Shoot P | 22.28  **(0.000)** | 176.76  **(0.000)** | 147.96  **(0.000)** | 2.46  (0.119) | 23.25  **(0.000)** | 191.01  **(0.000)** | 24.93  **(0.000)** |
| Shoot N | 262.29  **(0.000)** | 38.32  **(0.000)** | 6.38  **(0.000)** | 19.94  **(0.000)** | 23.15  **(0.000)** | 7.74  **(0.000)** | 8.36  **(0.000)** |
| Shoot ^33^P | 4.37  **(0.039)** | 0.698  (0.405) | 26.69  **(0.000)** | 52.52  **(0.000)** | 8.50  **(0.000)** | 4.04  **(0.009)** | 3.00  **(0.033)** |
| Shoot ^15^N | 636.10  **(0.000)** | 11.84  **(0.000)** | 25.67  **(0.000)** | 22.20  **(0.000)** | 2.69  **(0.049)** | 11.69  **(0.000)** | 6.76  **(0.000)** |

Light regime: Light regime: none (no shading), both (both plants are shaded), bis (P.bisulcatum is shaded) and max (P.maximum is shaded); Mycorrhizal status: mycorrhizal and non-mycorrhizal; Plant species: *P.maximum* and *P.bisulcatum.*

**Table S5.** Results of two-way ANOVA of the effects of light regime and mycorrhizal status on root biomass, root P, root N, root ^33^P and root ^15^N in mixed system compared with mono system.

|  | Inoculum | Light regime | Inoculum × Light regime |
| --- | --- | --- | --- |
| Root biomass | 2.01 (0.161) | 2.71 (0.052) | 1.03 (0.386) |
| Root P | 0.07 (0.788) | 2.70 (0.053) | 2.82 **(0.046)** |
| Root N | 2.31 (0.134) | 1.170 (0.327) | 2.30 (0.086) |
| Root ^33^P | 29.31 **(0.000)** | 0.88 (0.459) | 1.59 (0.2) |
| Root ^15^N | 4.78 **(0.033)** | 3.61 **(0.018)** | 0.78 (0.508) |

Light regime: Light regime: none (no shading), both (both plants are shaded), bis (P.bisulcatum is shaded) and max (P.maximum is shaded); Mycorrhizal status: mycorrhizal and non-mycorrhizal.

**Table S6.** Results of three-way ANOVA of the effects of light regime, mycorrhizal status, and plant species on total excess ^13^C response in mixed system compared with mono system. F and p-values (the latter in brackets) are shown.

|  | Plant species | Inoculum | Light regime | Plant species × Inoculum | Plant species × Light regime | Inoculum × Light regime | Plant species × Inoculum × Light regime |
| --- | --- | --- | --- | --- | --- | --- | --- |
| Total excess ^13^C response (%) | 3.03  (0.089) | 21.41  **(0.000)** | 4.44  **(0.008)** | 4.96  **(0.031)** | 3.67  **(0.019)** | 2.05  (0.119) | 2.18  (0.103) |

Excess ^13^C total: sums of ^13^C excess in all compartments of the individual pots (excess ^13^C in plant 1+ excess^13^C in plant 2+ excess ^13^C in roots + excess ^13^C in soil)

**Table S7.** Results of two-way ANOVA of the effects of light regime and plant species (*P.bisulcatum* and *P.maximum*) on ^13^C excess in C16:1ω5 WCFA and ^13^C allocation to C16:1ω5 fatty acid in mycorrhizal pots in mixed system compared with mono system. F and p-values (the latter in brackets) are indicated. Significant p-values (≤0.05) are indicated in bold.

|  | Plant species | Light regime | Plant species × Light regime |
| --- | --- | --- | --- |
| ^13^C excess in C16:1ω5 WCFA response (%) | 25.37 **(0.000)** | 5.78 **(0.004)** | 0.85 (0.479) |
| ^13^C allocation to C16:1ω5 response (%) | 20.48 **(0.000)** | 3.65 **(0.028)** | 0.5 (0.686) |

WCFA: whole-cell fatty acid fraction of lipid extracts contains all neutral lipid (NLFA), glycolipid (GLFA) and phospholipid (PLFA) fatty acids, C16:1ω5: a signature fatty acid being a proxy of AM fungal abundance in soil.

**Table S8.** Results of one-way ANOVA of the effects of light regime on AM fungal abundance in root and soil (response values) in mycorrhizal mixed system compared to mono systems. F and p-values are indicated. Significant p-values (≤0.05) are indicated in bold.

|  | Light regime |
| --- | --- |
| AM fungal abundance in root (qPCR) (%) | 5.11 **(0.006)** |
| AM fungal abundance in soil (qPCR) (%) | 0.45 (0.74) |
| AM fungal abundance in soil (WCFA) (%) | 1.79 (0.174) |

Note: AM fungal abundance in root and soil was quantified either by quantitative real-time PCR (qPCR) targeting nuclear large ribosomal subunit (LSU) gene of the fungus *R.irregularis* or the concentration of C16:1ω5 in the whole-cell fatty acids (WCFA) of total lipid extracts from soils.

# Figures

### ^13^C labeling experimental set up in mono and mixed system


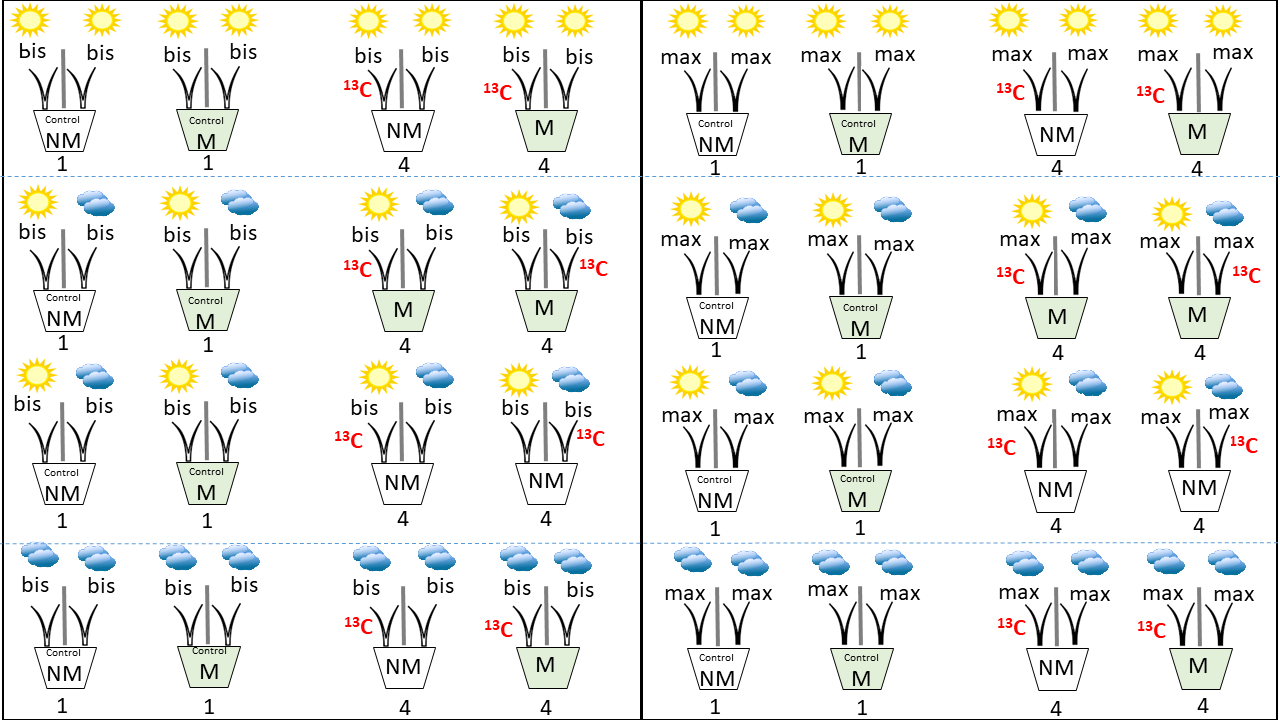


**Figure S1.** The schematic of the ^13^CO_2_ pulse labeling in mono system. Two plant species (*P.bisulcatum* on the left panel and *P.maximum* on the right panel) with two mycorrhizal statuses (mycorrhizal "M" or non-mycorrhizal "NM") were grown side by side as identical plant pairs and exposed to three light regimes *(*"full," "half," and "none")*.* For conducting ^13^CO_2_ pulse labeling, one replicate pot from each treatment combination (i.e., plant community diversity, mycorrhizal status, and shading pattern) was left unlabeled to estimate natural isotopic abundance of ^13^C. Other four replicates of mono system treatments exposed to homogeneous light conditions (either full light or full shade) were processed as follows: one of the plant individuals per pot was tightly wrapped in aluminum foil before moving the pots under the labeling canopy (to prevent any fixation of ^13^CO_2_ during the labeling), whereas the other plant was left to photosynthesize under the labeling canopy.


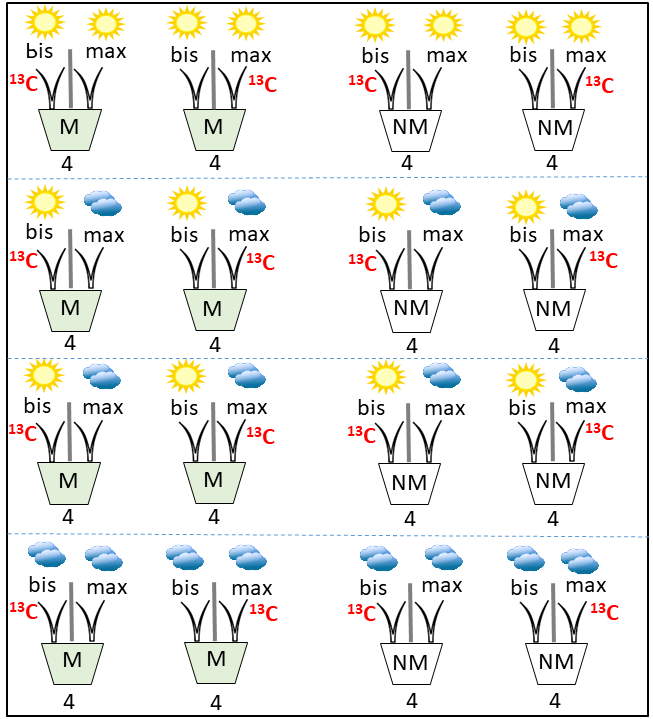


**Figure S2.** The schematic of the ^13^CO_2_ pulse labeling in mixed system. Plant species (*P.bisulcatum* and *P.maximum*) with two mycorrhizal statuses (mycorrhizal “M” or non-mycorrhizal “NM”) were grown side by side as pairs of different plant species and exposed to four light regimes. For conducting ^13^CO_2_ pulse labeling, four replicates with the “left” and four with the “right” plant individual enwrapped in aluminum foil to prevent their photosynthesis during ^13^CO_2_ labeling were included into the experiment.

| 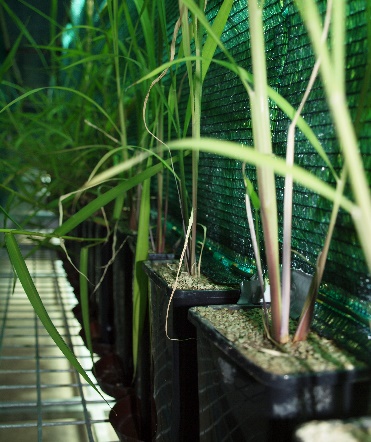  **a)** | 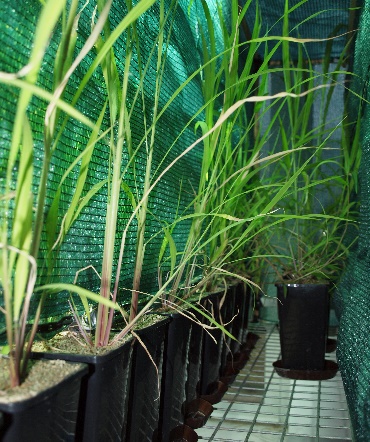  **b)** | 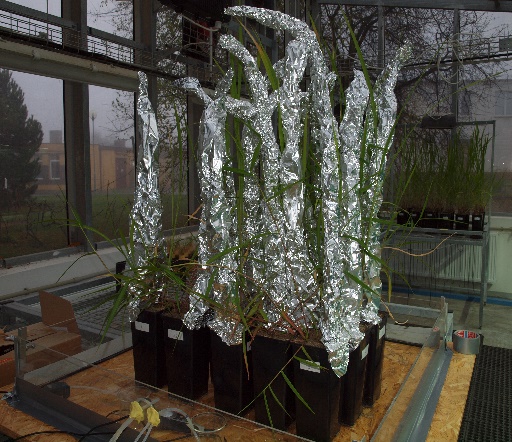  **c)** |
| --- | --- | --- |
| 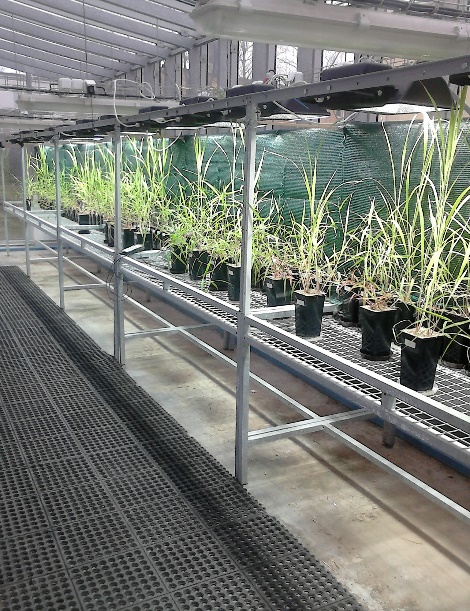 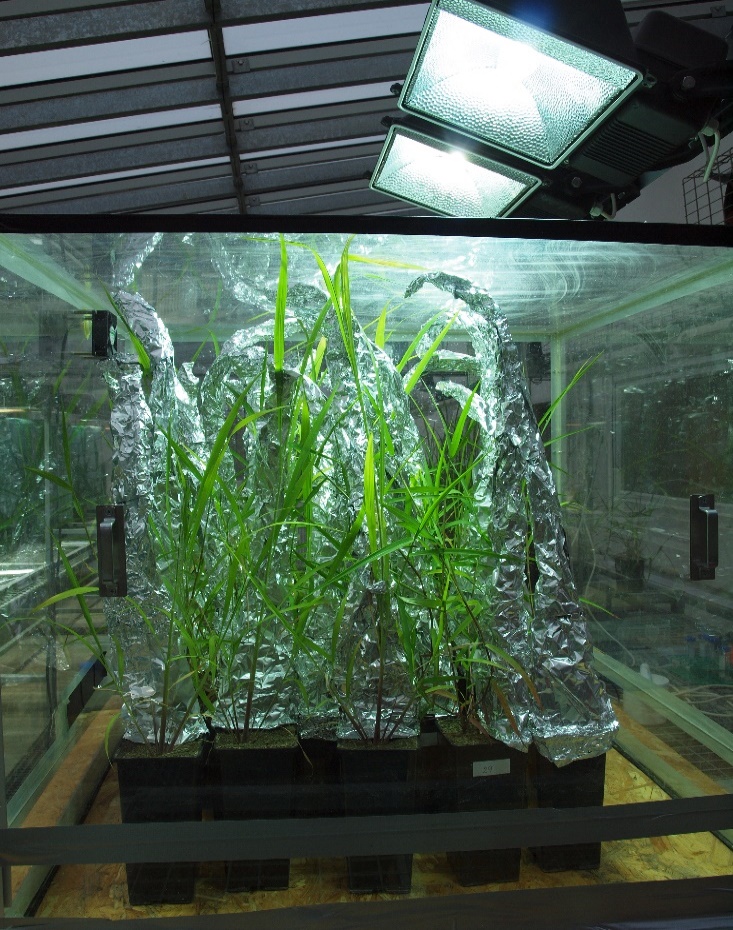  **f)**  **d)** | | |

**Figure S3.** Experimental images showing pots under “half” (a and b) and “none” shading regime (d), enwrapping one plant individual per pot in aluminum foil (c) and plants in labeling chamber (f).

## Mono system

### Plant biomass

| (a) Shoot dry biomass | |
| --- | --- |
| 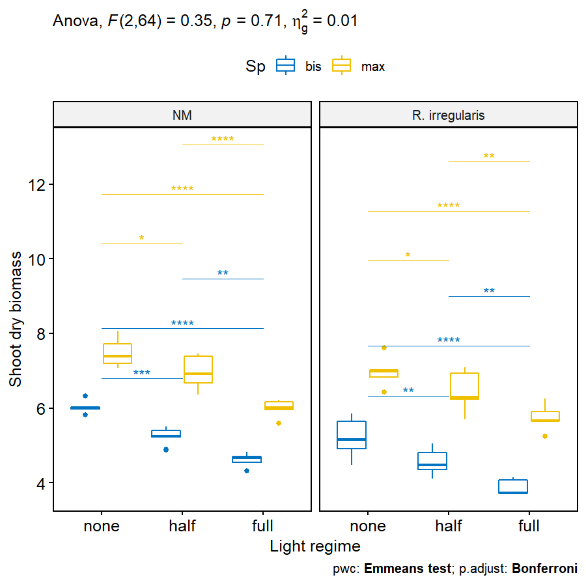 | 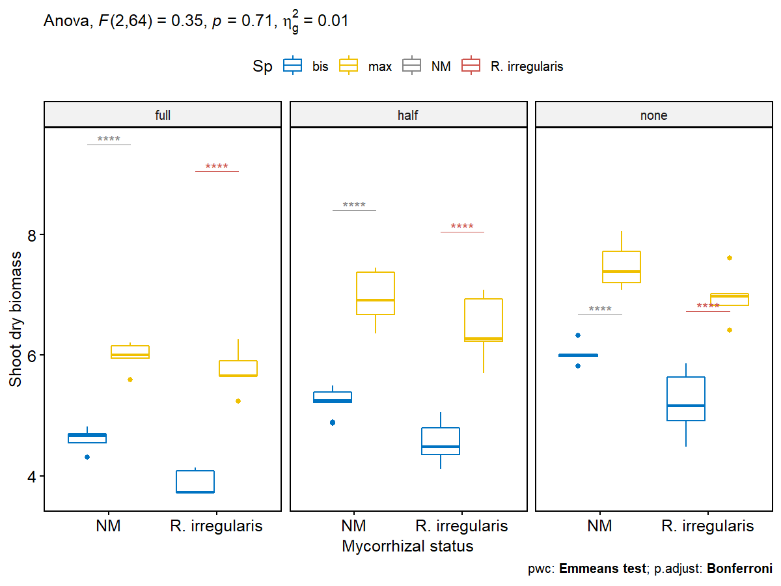 |
| (b) Root dry biomass | |
| 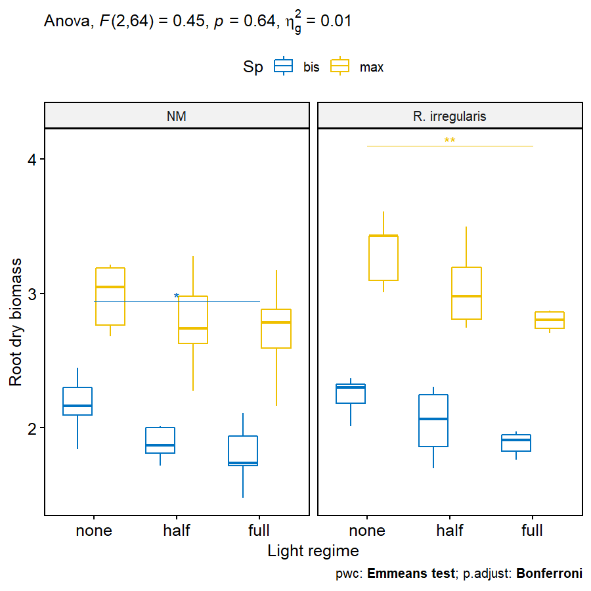 | 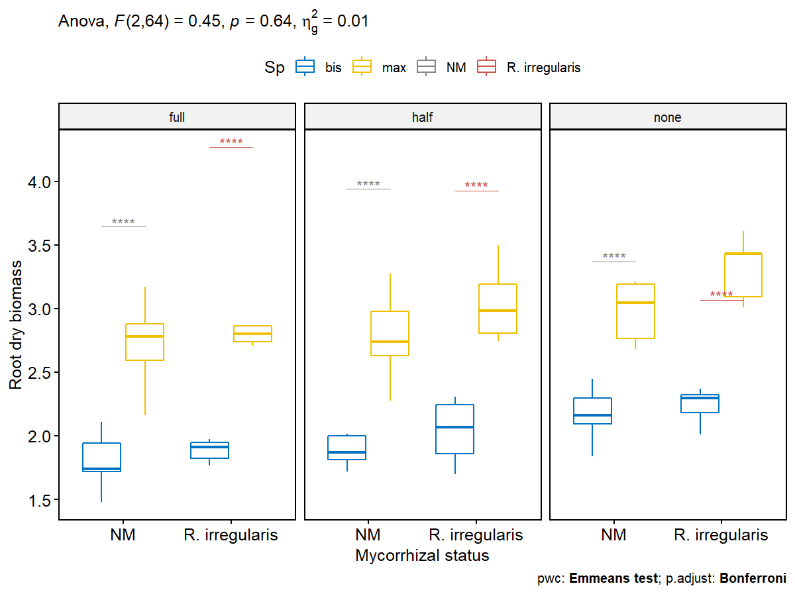 |

| (c) Biomass partitioning (shoot to root ratio) | |
| --- | --- |
| 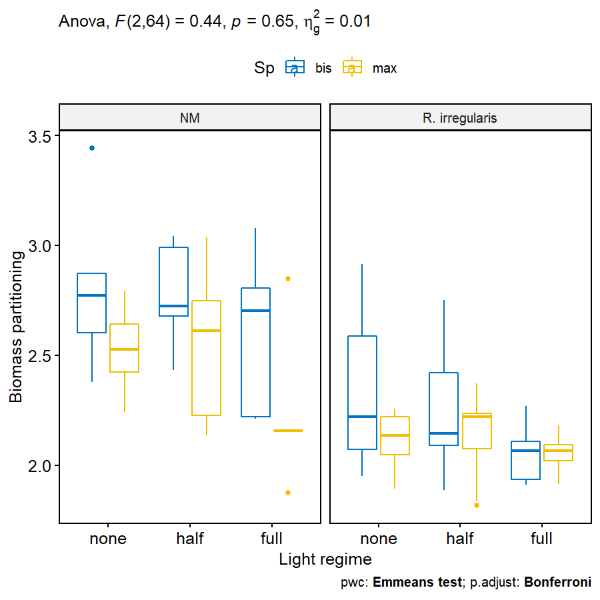 | 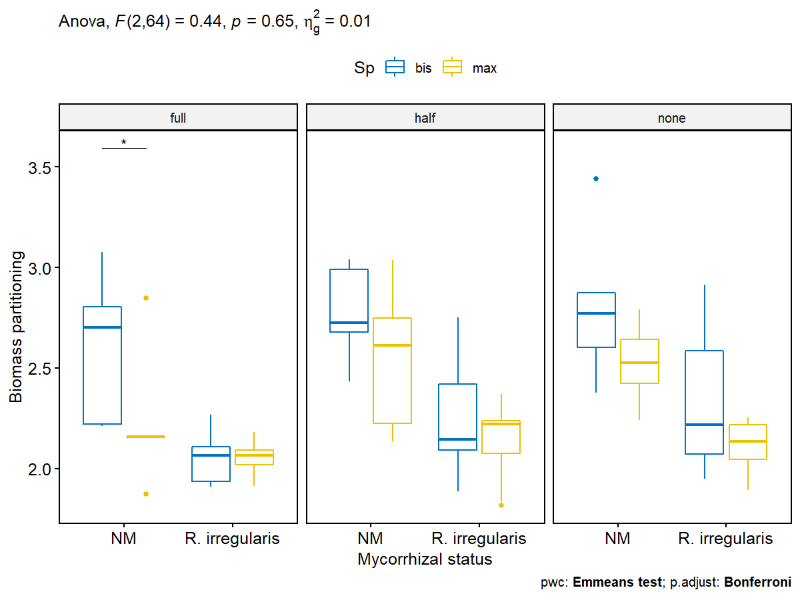 |
| (d) Total dry biomass (shoot plus roots) | |
| 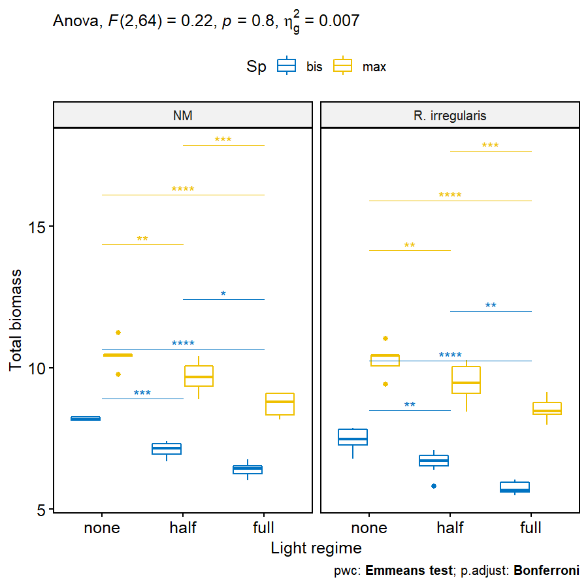 | 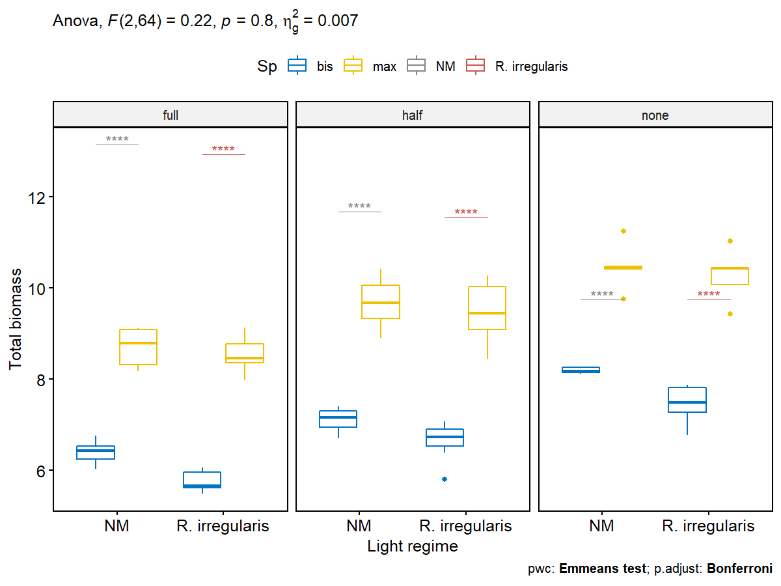 |

**Figure S4.** Three-way ANOVA of the effects of light regime, mycorrhizal status, and plant species in the mono system on (a) shoot dry biomass, (b) root dry biomass, (c) biomass partitioning (shoot to root dry weight) and (d) total dry biomass of the plants. Post-hoc multiple pairwise comparisons between groups were performed using the estimated marginal means and p-values were adjusted using the Bonferroni correction. The ‘’bis’’ and ‘’max’’ refer to *P.maximum* and *P.bisulcatum,* respectively*.* ‘’NM’’ and ‘’R.irregularis’’ refer to the non-mycorrhizal and mycorrhizal (inoculated with *Rhizophagus irregularis*) status of the plants, respectively. ''None'', ''half'' and ''full'' refer to not shaded, half shaded, and fully shaded pots, respectively. Asterisks indicate levels of significance; p ≤ 0.05 (*), p ≤ 0.01 (**), p ≤ 0.001 (***) and p ≤ 0.0001 (****).

### P and ^33^P uptake

| (a) Shoot P | |
| --- | --- |
| 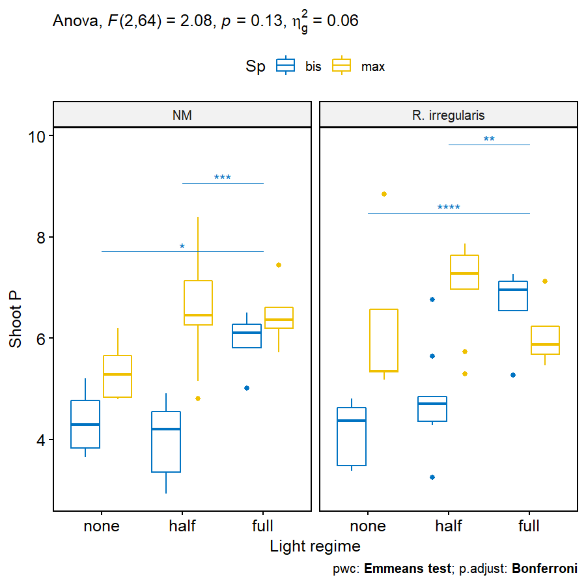 | 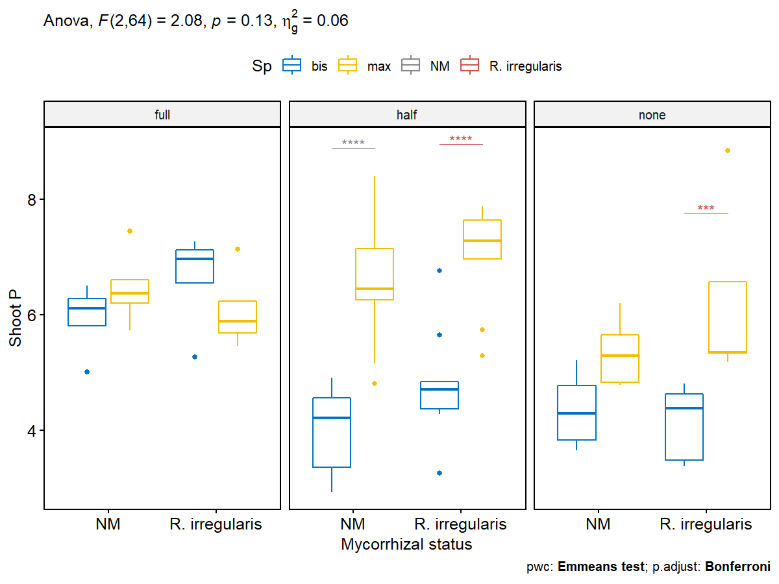 |
| (b) Root P | |
| 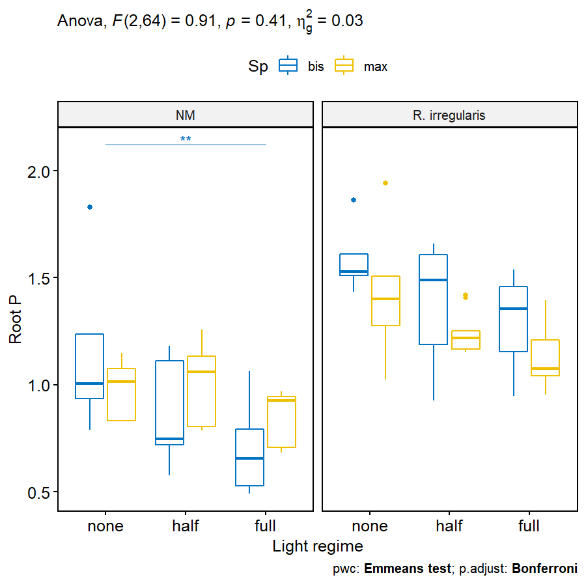 | 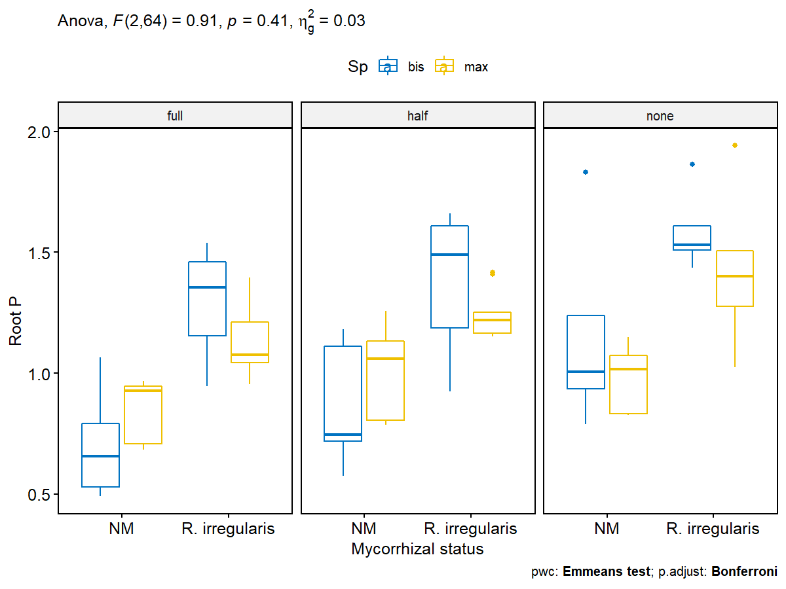 |

| (c) P partitioning (Shoot to root P) | |
| --- | --- |
| 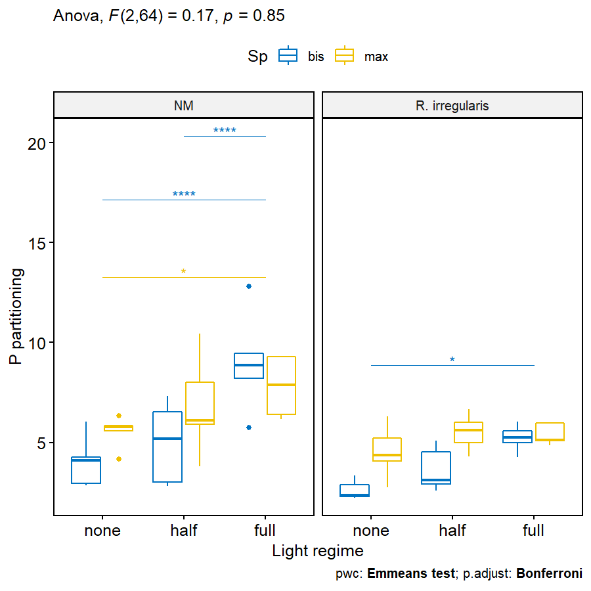 | 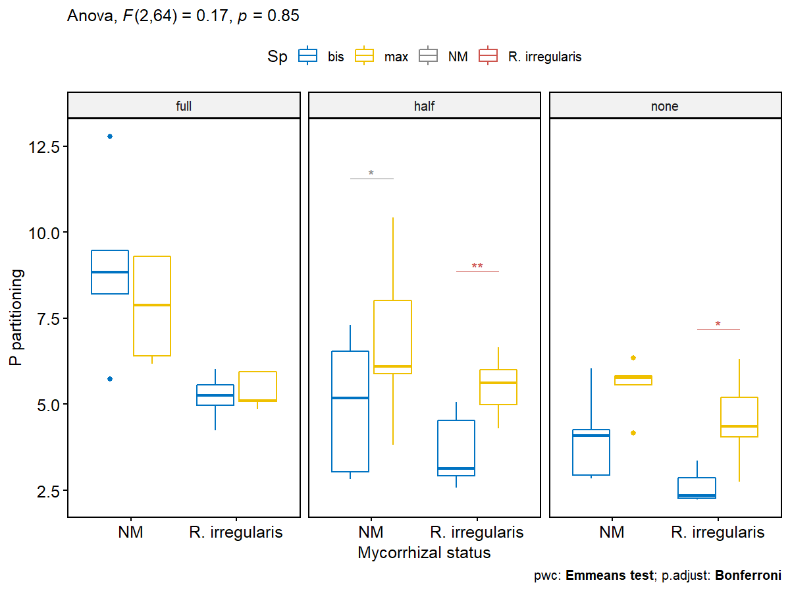 |
| (d) Total P (shoot plus root) | |
| 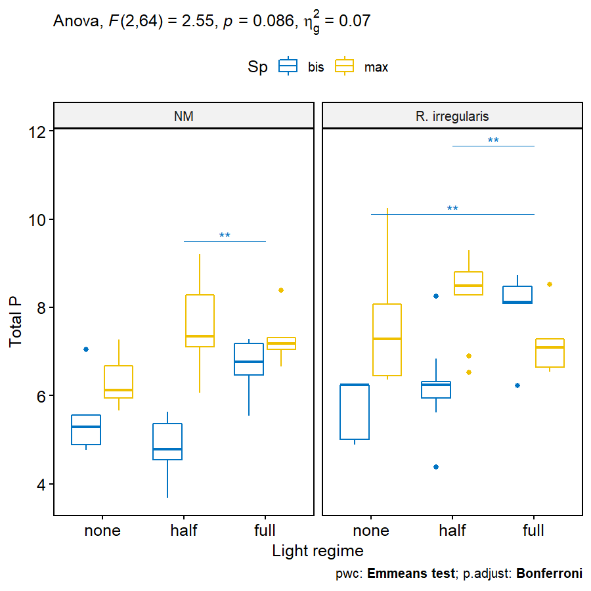 | 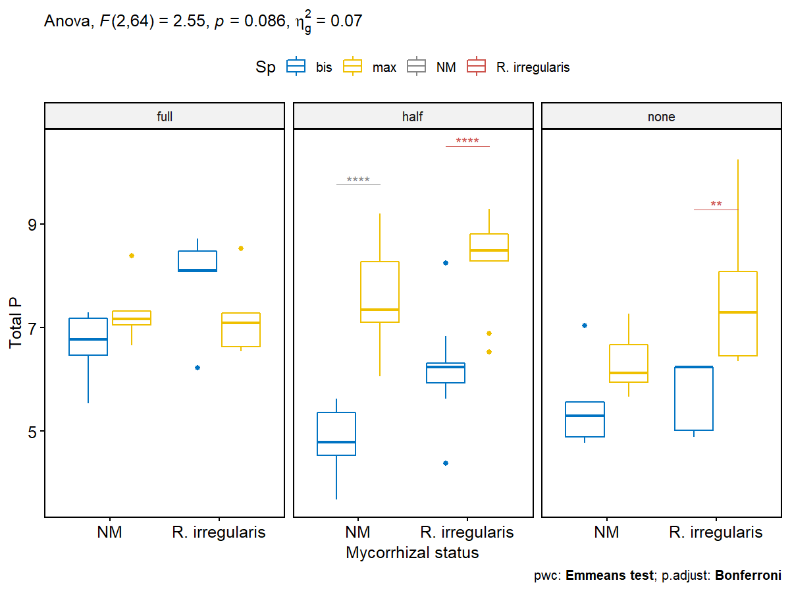 |

| (e) Shoot ^33^P | |
| --- | --- |
| 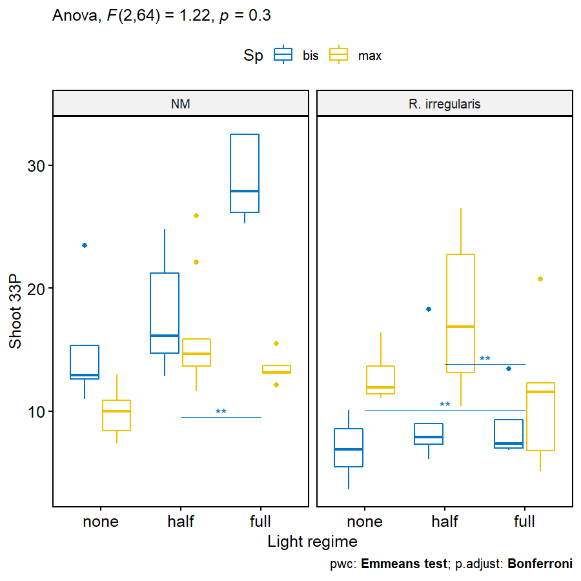 | 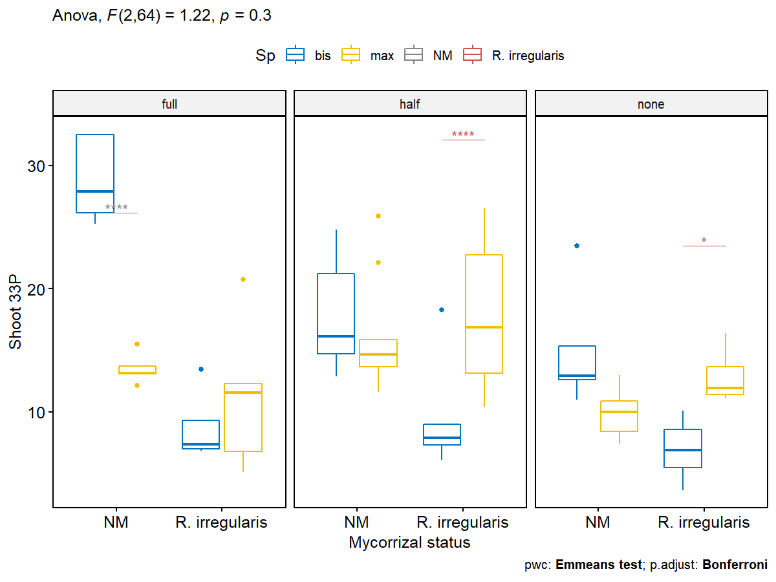 |
| (f) Root ^33^P | |
| 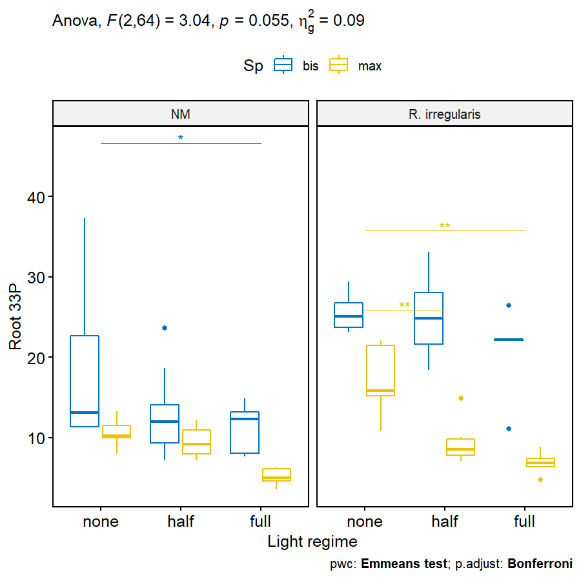 | 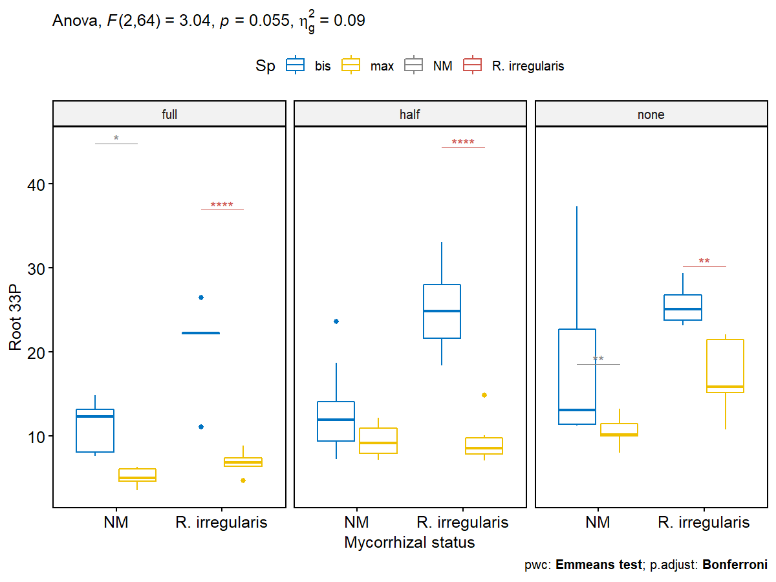 |
| (g) ^33^P partitioning |  |
| 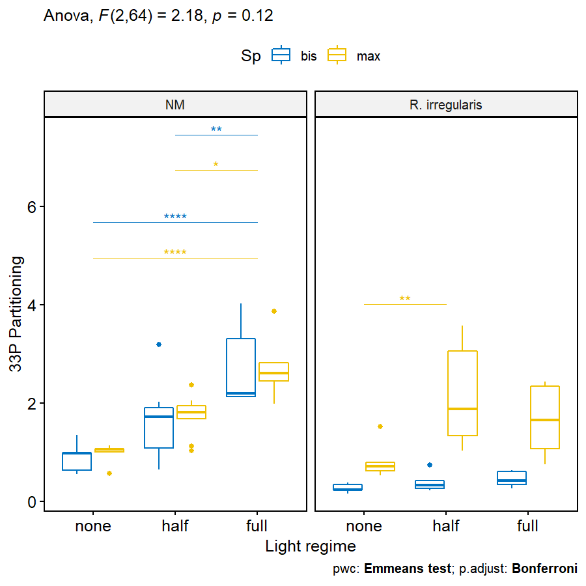 | 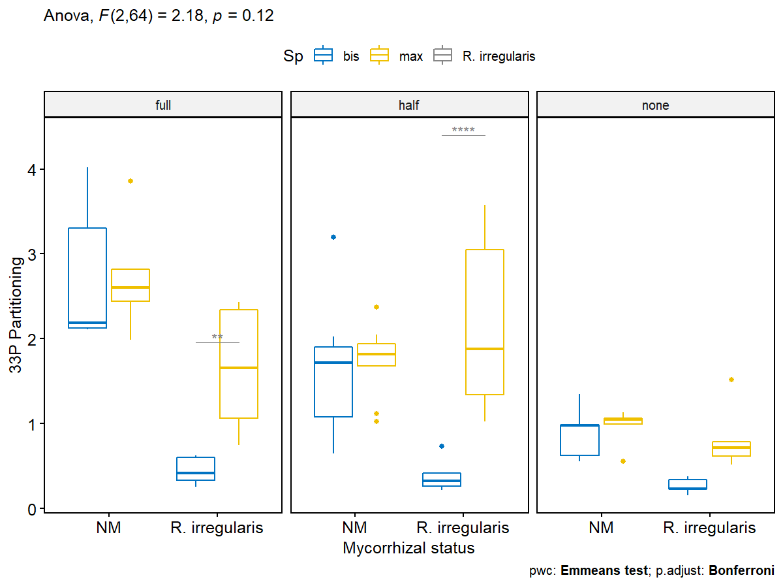 |

| (h) Soil ^33^P | |
| --- | --- |
| 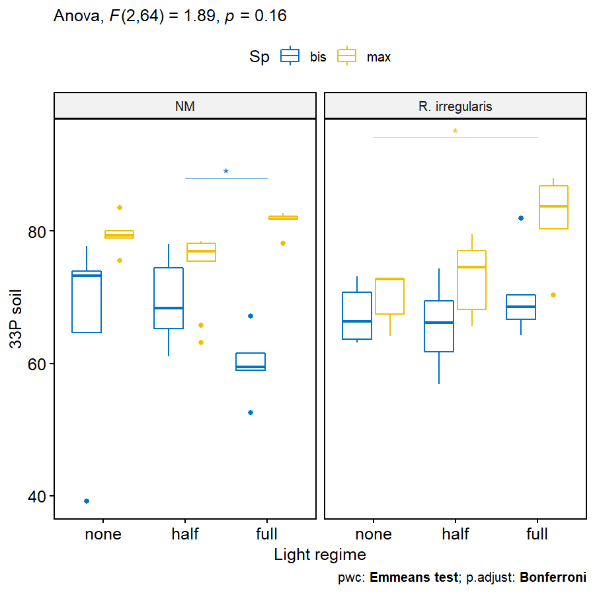 | 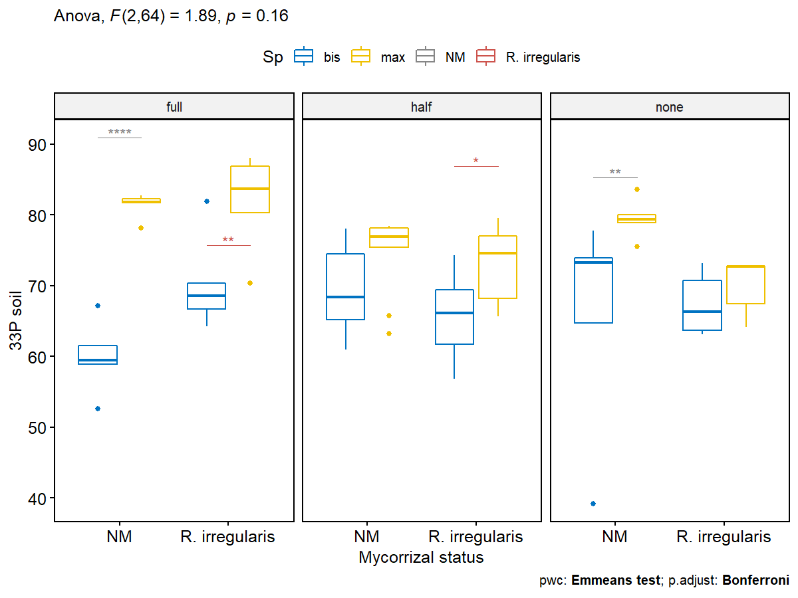 |
| (i) Total ^33^P (shoot plus root) | |
| 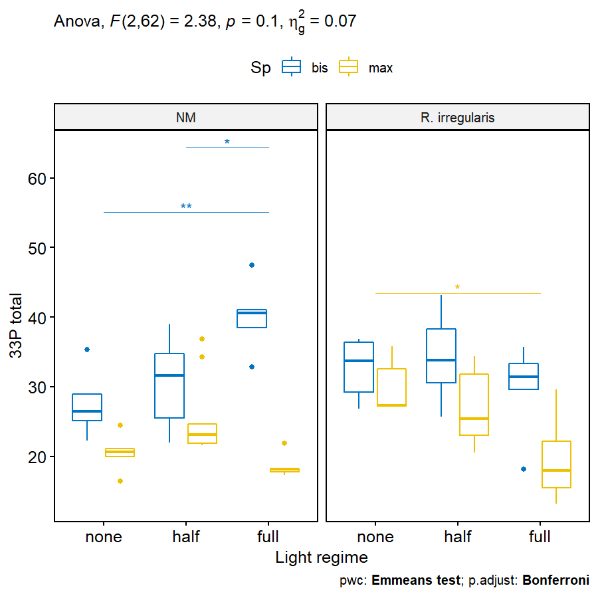 | 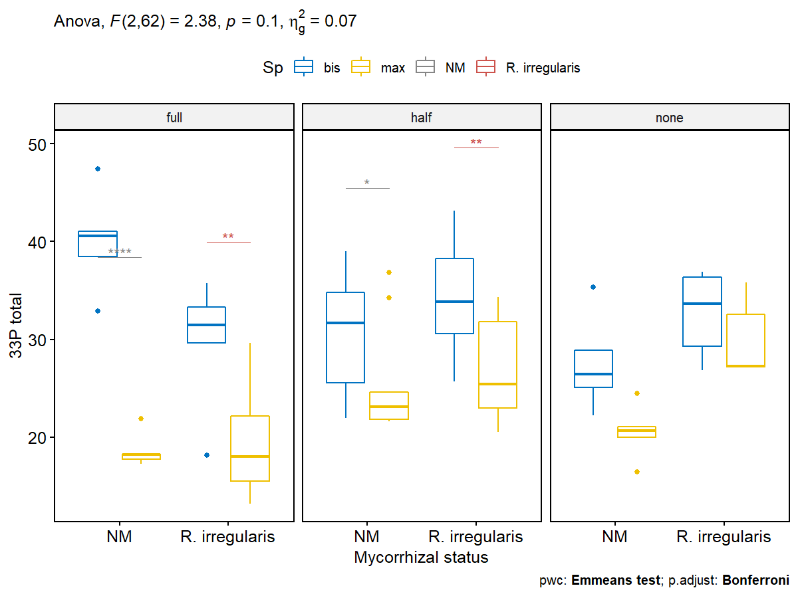 |

**Figure S5.** Three-way ANOVA of the effects of light regime, mycorrhizal status, and plant species in the mono system on (a) shoot P content (mg/pot), (b) root P (mg/pot), (c) P partitioning (Shoot to root P content), (d) total P (shoot plus root P content), (e) shoot ^33^P (% applied radioactivity transported to plant shoots), (f) root ^33^P (% applied radioactivity detected in the roots), (g) shoot to root ^33^P (ratio of ^33^P activity in shoot to roots), (h) soil ^33^P (% radioactivity remaining in the soil) and (i) total ^33^P (shoot plus root, % ^33^P activity injected per pot and detected in any plant tissues). Post-hoc multiple pairwise comparisons between groups were performed using the estimated marginal means and p-values were adjusted using the Bonferroni correction. The ‘’bis’’ and ‘’max’’ refer to *P.maximum* and *P.bisulcatum,* respectively*.* ‘’NM’’ and ‘’R.irregularis’’ refer to the non-mycorrhizal and mycorrhizal (inoculated with *Rhizophagus irregularis*) status of the plants, respectively. ''None'', ''half'' and ''full'' refer to not shaded, half shaded, and fully shaded pots, respectively. Asterisks indicate levels of significance; p ≤ 0.05 (*), p ≤ 0.01 (**), p ≤ 0.001 (***) and p ≤ 0.0001 (****).

### N and ^15^N uptake

| (a) Shoot N | |
| --- | --- |
| 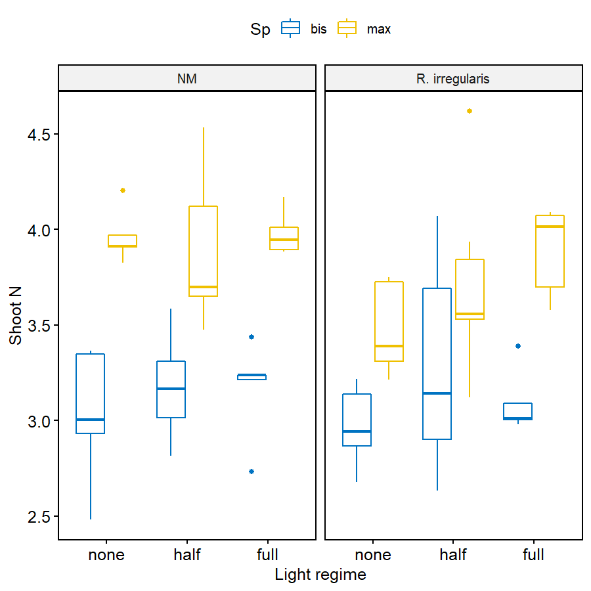 | 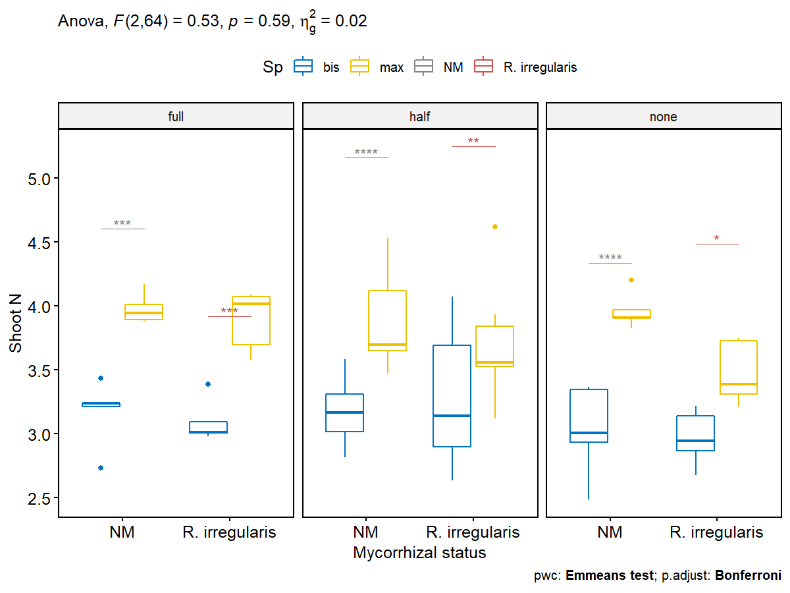 |
| (b) Root N | |
| 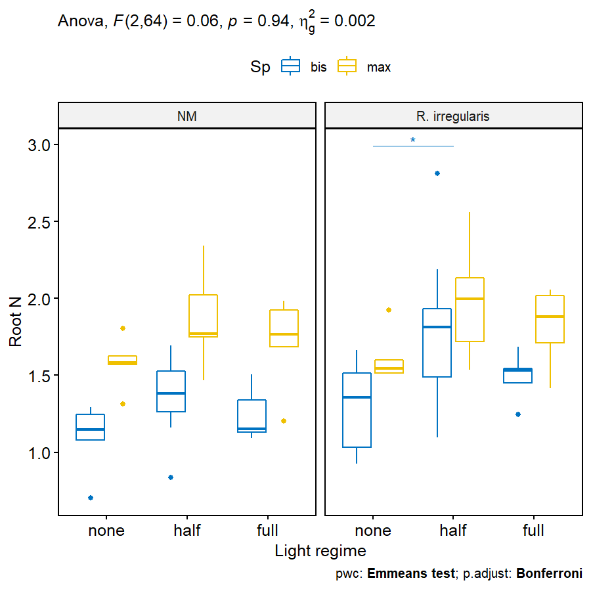 | 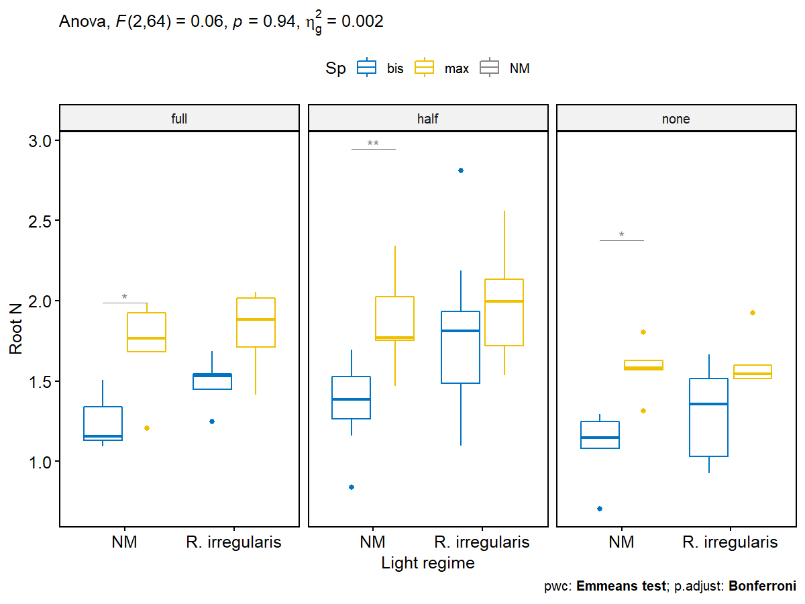 |

| (c) N partitioning (shoot to root N) | |
| --- | --- |
| 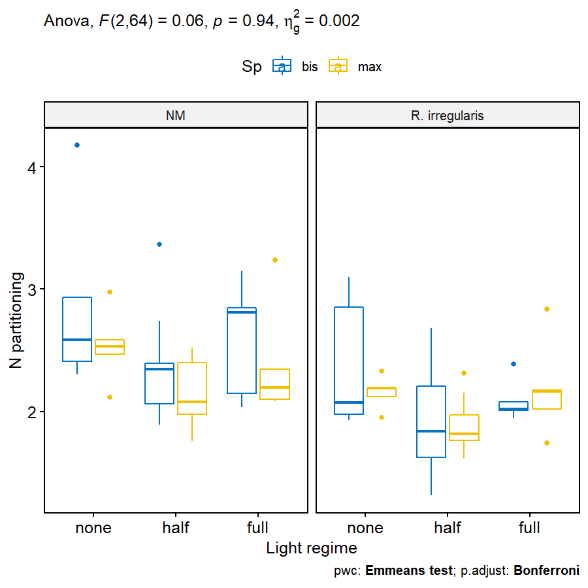 | 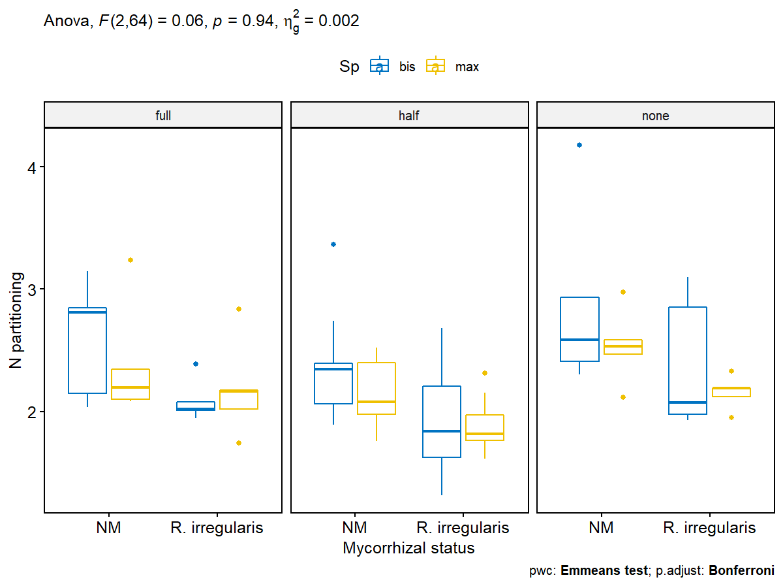 |
| (d) Total N (shoot plus root) | |
| 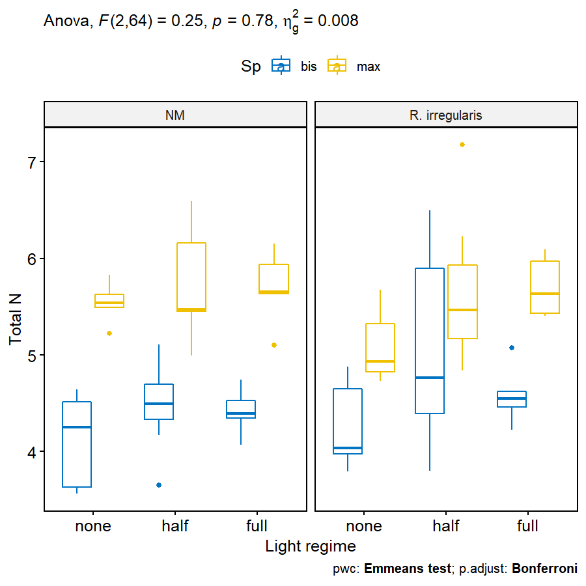 | 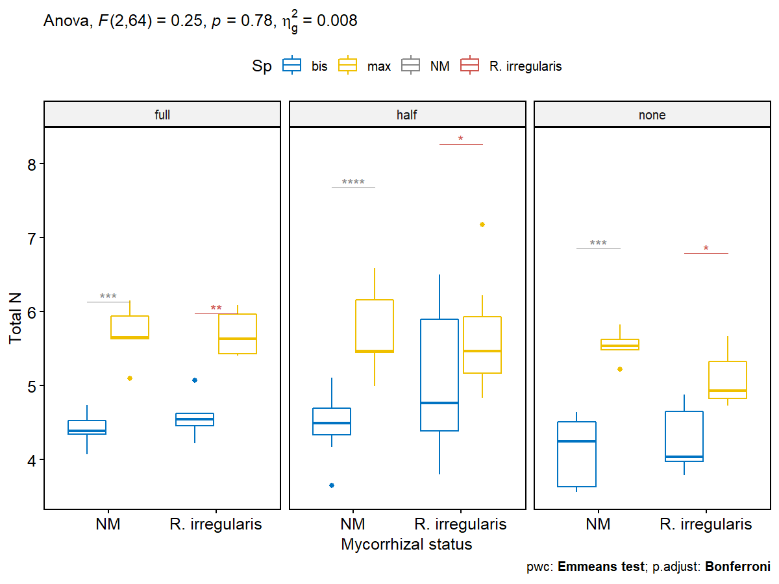 |

| (e) Shoot ^15^N | |
| --- | --- |
| 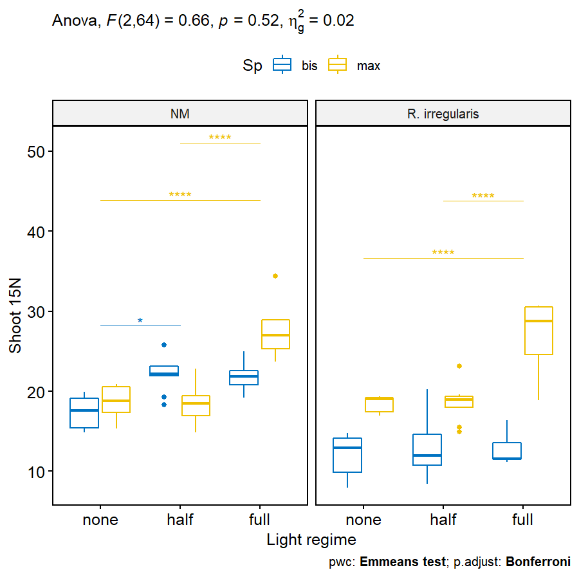 | 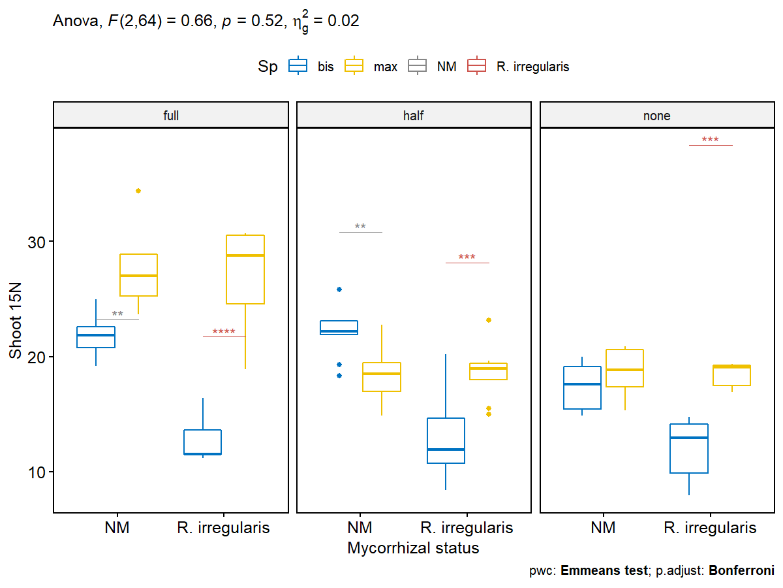 |
| (f) Root ^15^N |  |
| 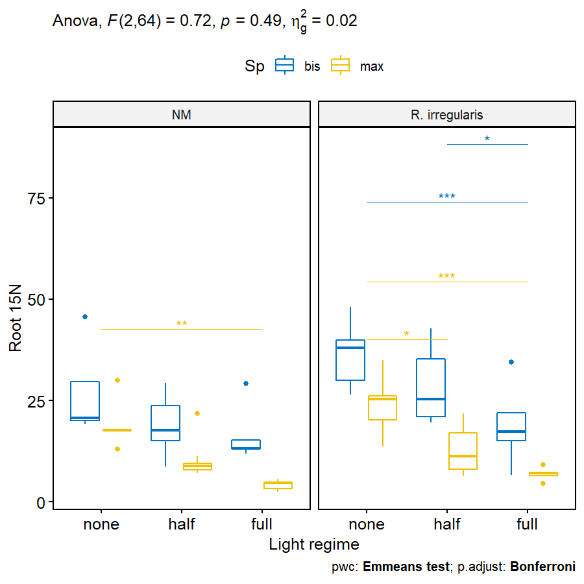 | 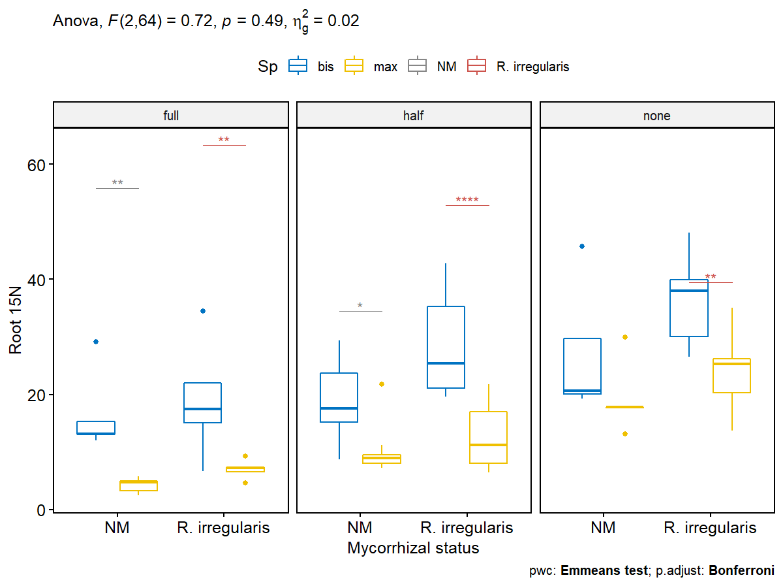 |
| (g) ^15^N partitioning (shoot to root ^15^N) | |
| 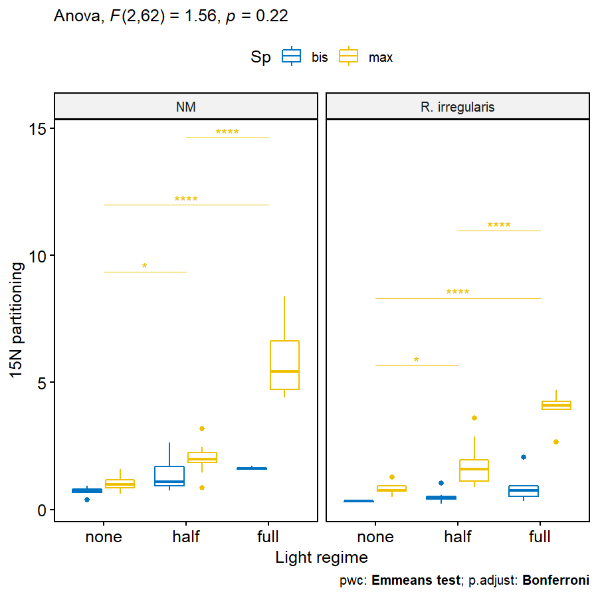 | 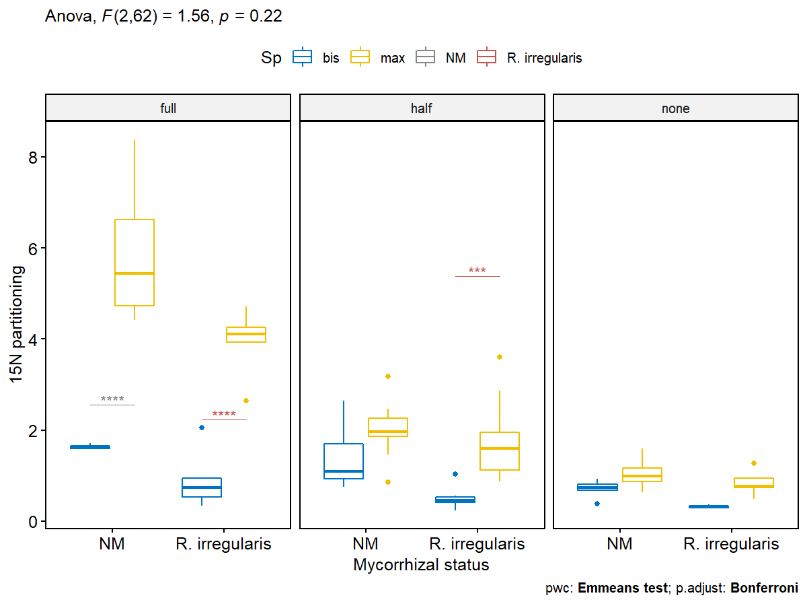 |

| (h) Soil ^15^N | |
| --- | --- |
| 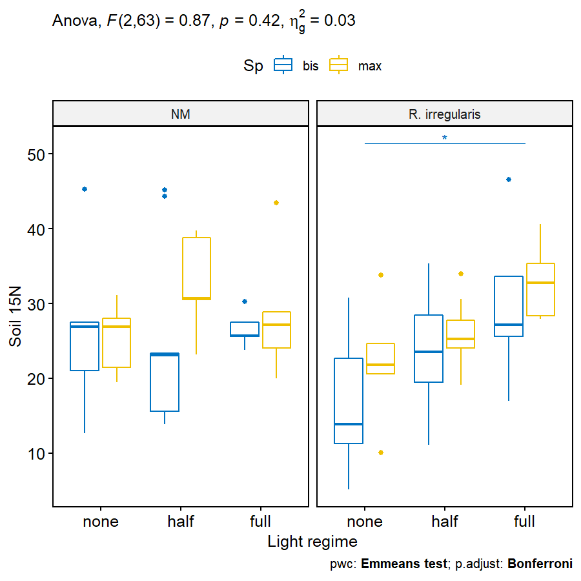 | 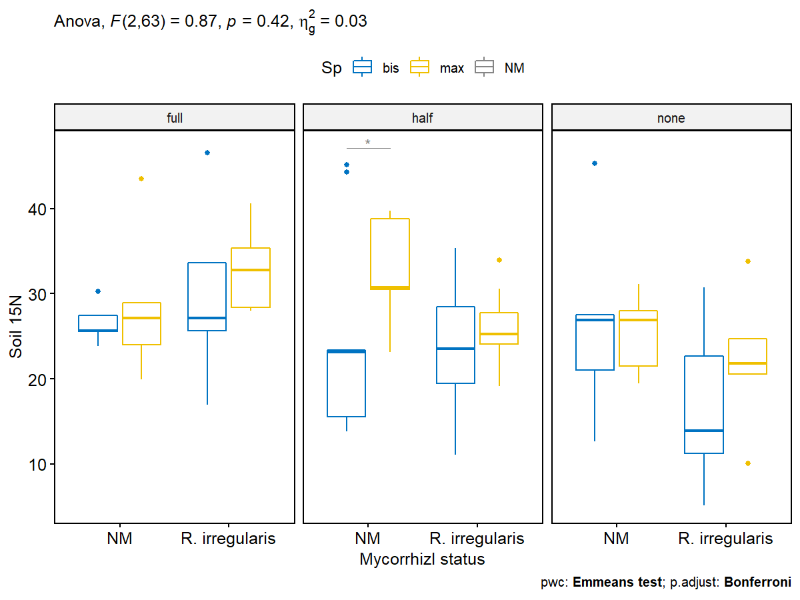 |
| (i) Total ^15^N |  |
| 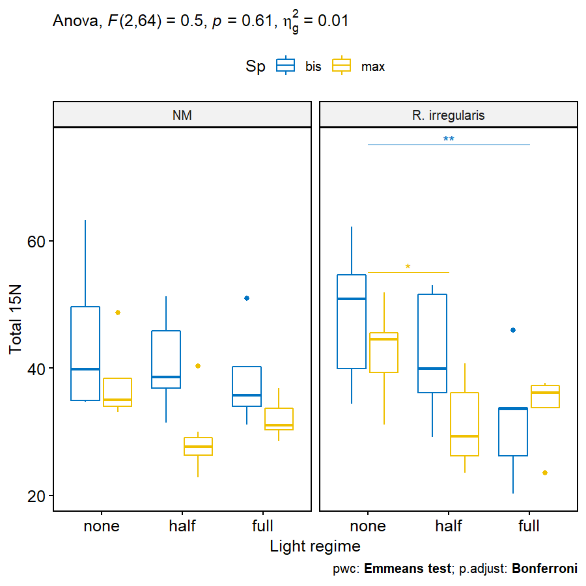 | 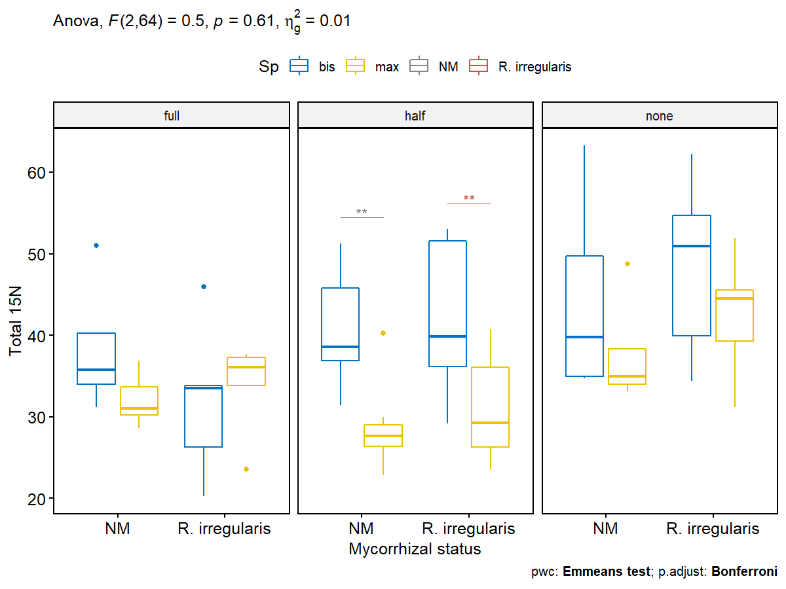 |

**Figure S6.** Three-way ANOVA of the effects of light regime, mycorrhizal status, and plant species in the mono system on (a) shoot N content (mg/pot), (b) root N (mg/pot), (c) N partitioning (shoot to root N), (d) Total N (shoot N content plus root N content), (e) Root ^15^N (% of ^15^N excess detected in the root samples), (f) Shoot ^15^N (% of ^15^N excess detected in the shoots), (g) ^15^N partitioning (shoot to root ^15^N, ratio of ^15^N allocation to shoot to ^15^N allocation to roots), (h) Soil ^15^N (% of ^15^N excess detected in the soil samples) and (i) Total ^15^N (% of ^15^N excess in shoots + roots per pot). Post-hoc multiple pairwise comparisons between groups were performed using the estimated marginal means and p-values were adjusted using the Bonferroni correction. The ‘’bis’’ and ‘’max’’ refer to *P.maximum* and *P.bisulcatum,* respectively*.* ‘’NM’’ and ‘’R.irregularis’’ refer to the non-mycorrhizal and mycorrhizal (inoculated with *Rhizophagus irregularis*) status of the plants, respectively. ''None'', ''half'' and ''full'' refer to not shaded, half shaded, and fully shaded pots, respectively. Asterisks indicate levels of significance; p ≤ 0.05 (*), p ≤ 0.01 (**), p ≤ 0.001 (***) and p ≤ 0.0001 (****).

### ^13^C allocation

| **(a) ^13^C excess in WCFA (µmol pot^-1^)** | **(b) ^13^C allocation to AMF (C16:1ω5) (%)** |
| --- | --- |
| 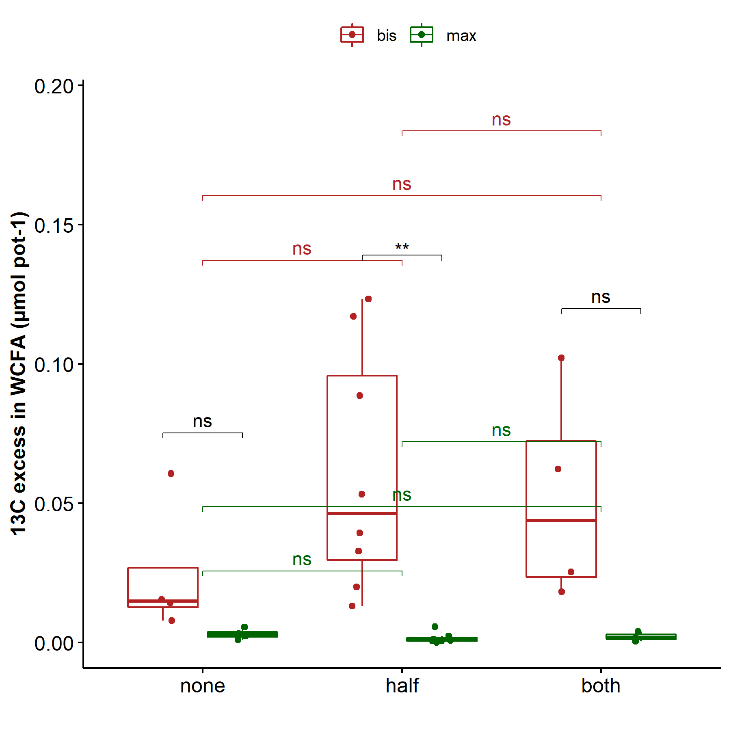 | 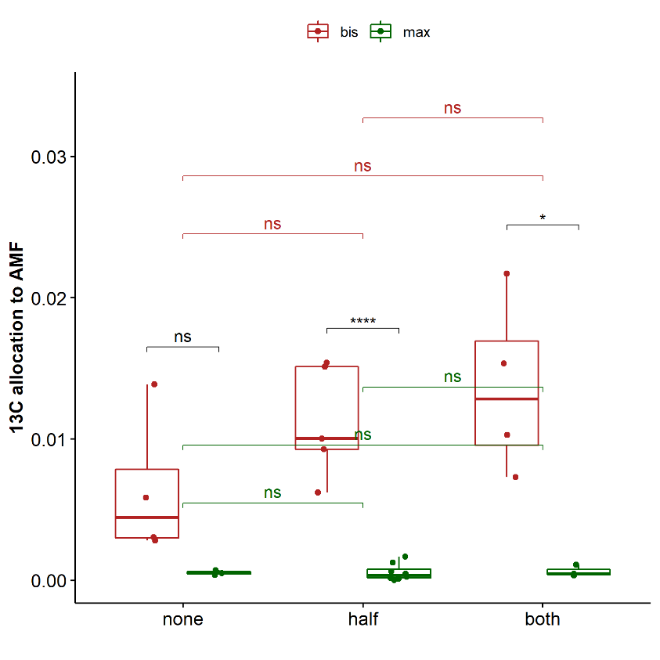 |

**Figure S7.** Two-way ANOVA of the effects of light regime and plant species in the mono system on (a) ^13^C excess in WCFA (whole-cell fatty acid C16:1ω5, i.e. this acid present in different chemical forms in the cell, such as within neutral-, glyco- and phosphor-lipids), (b) ^13^C allocation to C16:1ω5 as compared to the entire ^13^C excess detected in all pot compartments. Post hoc multiple pairwise comparisons between groups were performed using the estimated marginal means and p-values were adjusted using the Bonferroni correction. The ‘’bis’’ and ‘’max’’ refer to *P.bisulcatum* and *P.maximum,* respectively. Only mycorrhizal pots were considered in this analysis. ''None'', ''half'' and ''full'' refer to not shaded, half shaded, and fully shaded pots, respectively. Asterisks indicate levels of significance; p ≤ 0.05 (*), p ≤ 0.01 (**), p ≤ 0.001 (***) and p ≤ 0.0001 (****).

### Mycorrhizal colonization

| (a) AMF abundance in root (qPCR, LSU copies mg^-1^ root) | (b) AMF abundance in soil (qPCR, LSU copies g^-1^ soil) |
| --- | --- |
| 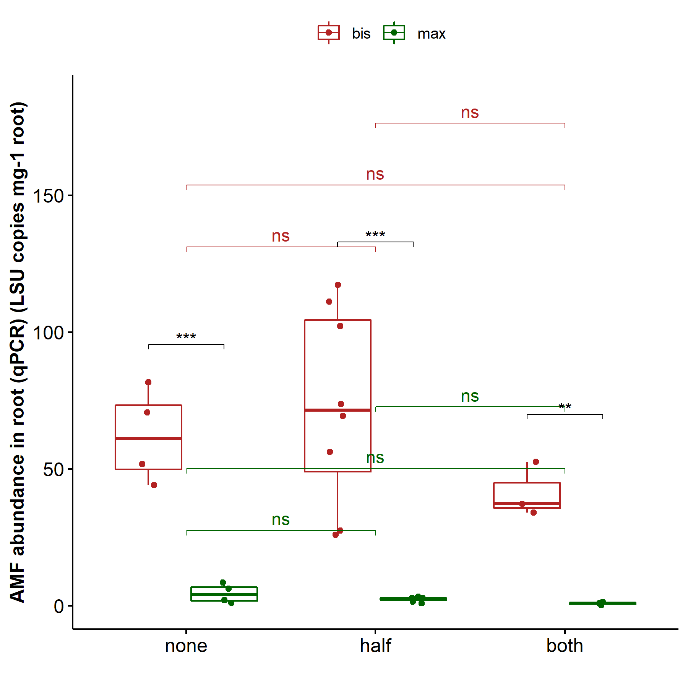 | 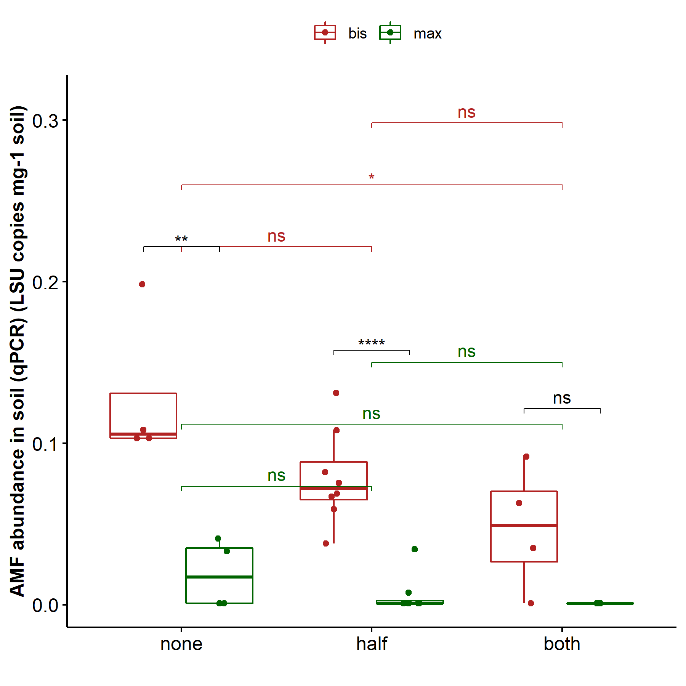 |
| (c) AMF abundance in soil (C16:1ω5 concentration µg C g^-1^ soil) | |
| 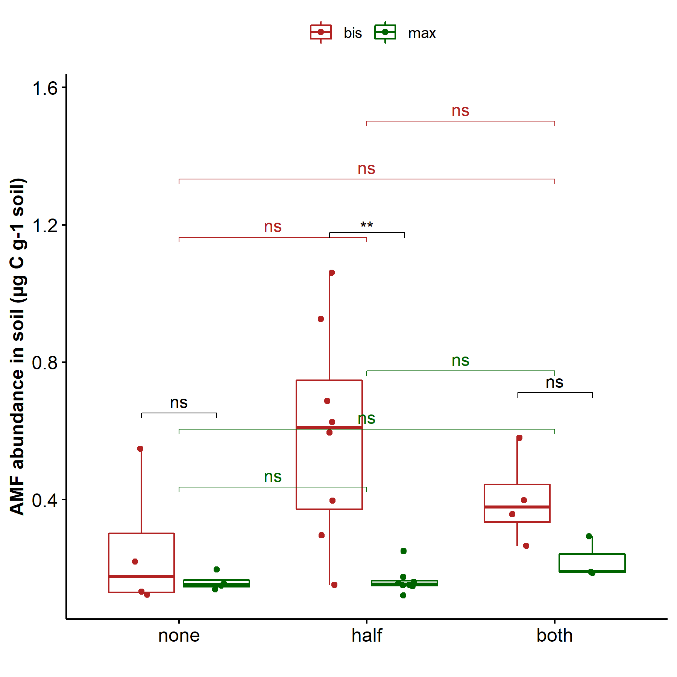 |  |

**Figure S8.** Two-way ANOVA of the effects of light regime and plant species in the mono system on (a) AMF abundance in root (measured by qPCR, million gene copies per mg root dry weight), (b) AMF abundance in soil (measured by qPCR, millions per g soil dry weight), and (c) AMF abundance in soil (measured by whole-cell fatty acid analysis (WCFA) using the C16:1ω5 biomarker). Post hoc multiple pairwise comparisons between groups were performed using the estimated marginal means and p-values were adjusted using the Bonferroni correction. The ‘’bis’’ and ‘’max’’ refer to *P.bisulcatum* and *P.maximum,* respectively*.* Only mycorrhizal pots were considered in this analysis. ‘’None’’, ‘’half’’ and ‘’full’’ refer to not shaded, half shaded, and fully shaded pots, respectively. Asterisks indicate levels of significance; p ≤ 0.05 (*), p ≤ 0.01 (**), p ≤ 0.001 (***) and p ≤ 0.0001 (****).

### ^33^P, ^15^N and ^13^C budgets in mono system

| ***P.bisulcatum*** | ***P.maximum*** |
| --- | --- |
| 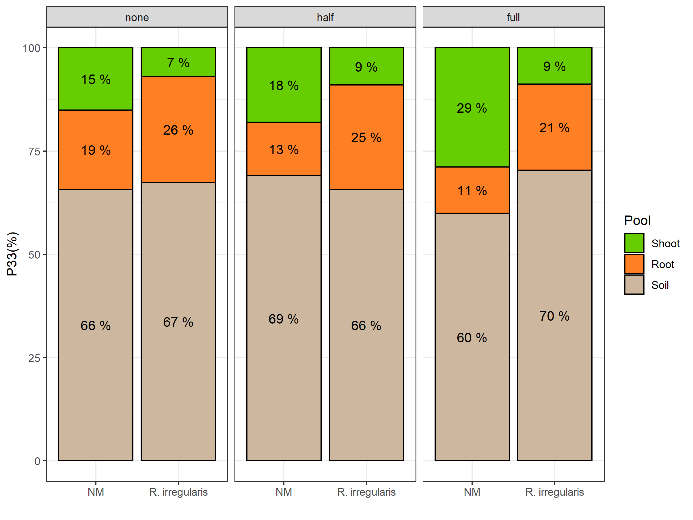 | 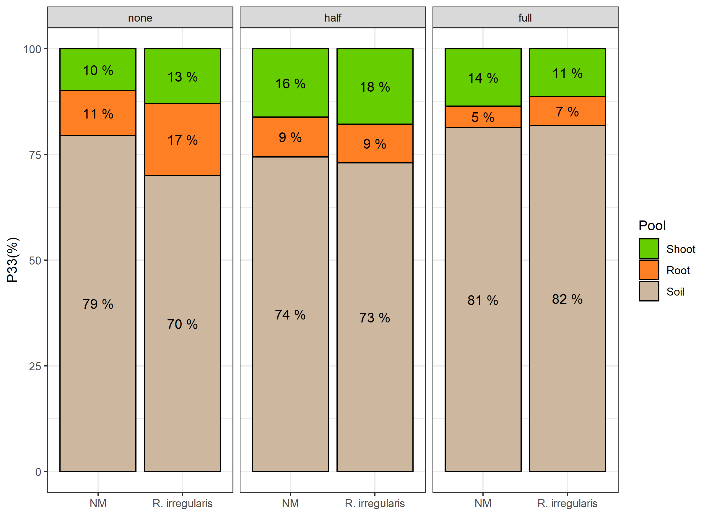 |
| 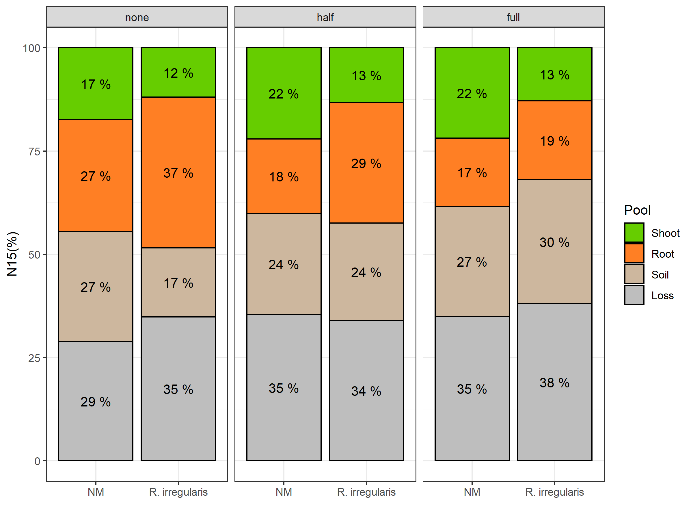 | 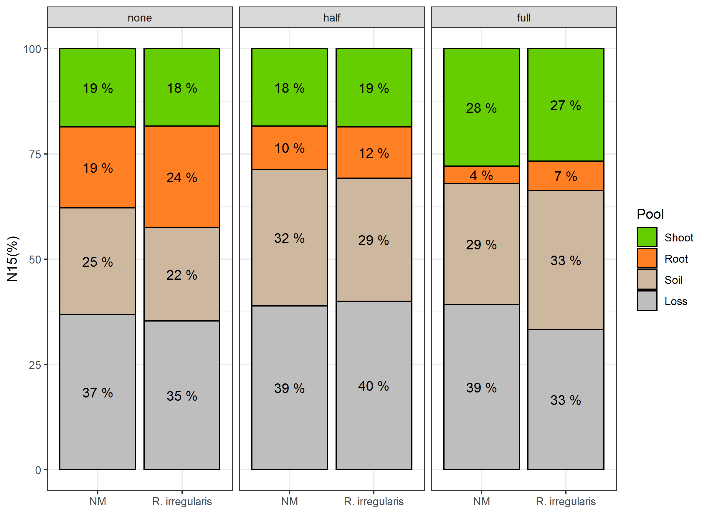 |
| 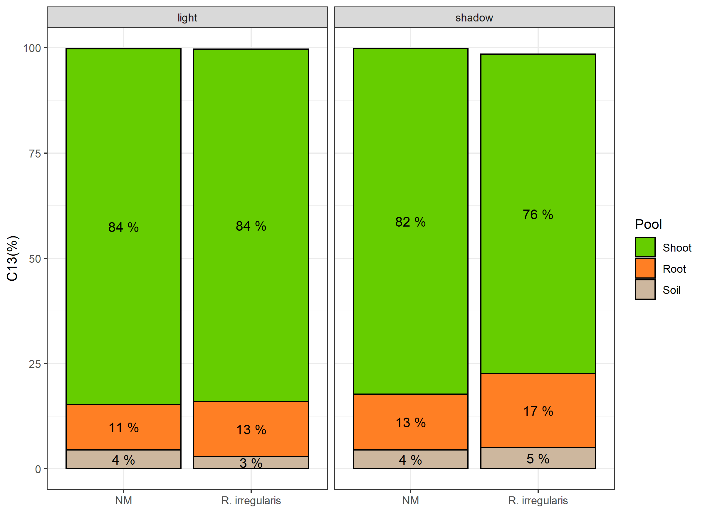 | 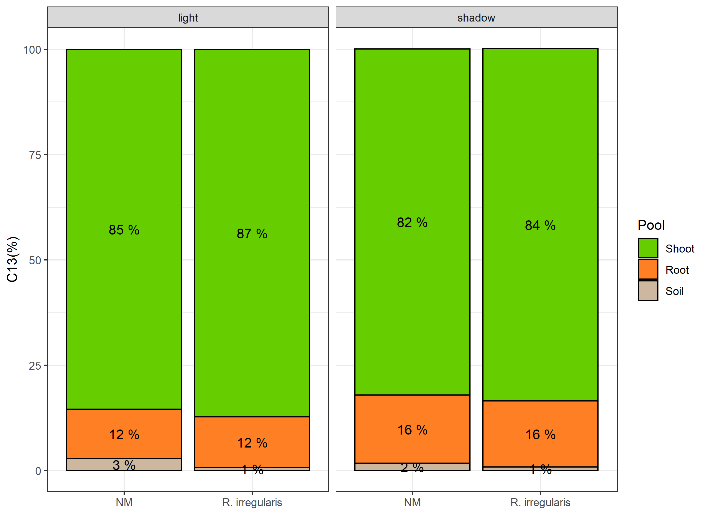 |

**Figure S9.** ^33^P, ^15^N and ^13^C budgets in *P.bisulcatum* and *P.maximum* monoculture pots under different light regimes and mycorrhizal statuses. NM – nonmycorrhizal plants, R.irregularis – inoculated with *Rhizophagus irregularis*. Share of ^33^P in soil is obtained by subtraction of ^33^P allocated to shoots and roots per pot from the total ^33^P radioactivity supplied per pot. Share of ^15^N allocated to losses is calculated by subtraction of excess ^15^N detected in shoots, roots and soil from the total ^15^N amount supplied per pot. Share of ^13^C in different compartments is established from the total amount of excess ^13^C detected in all measured system compartments (i.e., shoots of both plants, roots, and soil, on a per pot basis).

## Mixed system compared to mono system

### Shoot biomass-, P-, N-, ^33^P- and ^15^N-responses

| a) Shoot biomass response | | |
| --- | --- | --- |
| 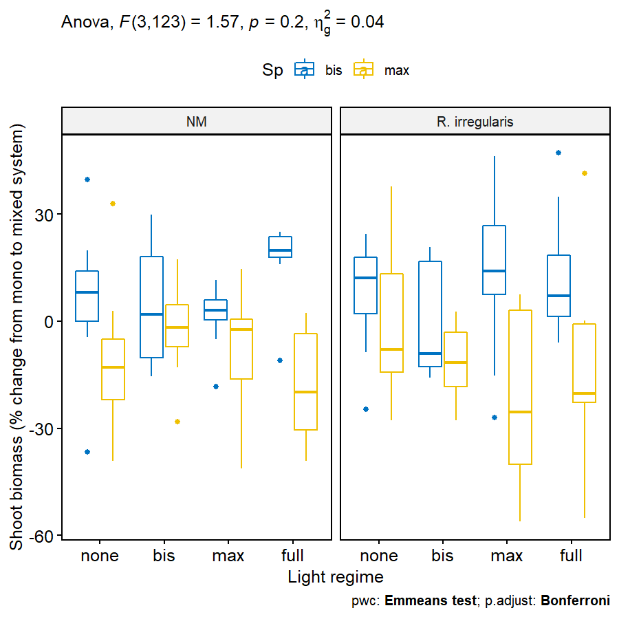 | 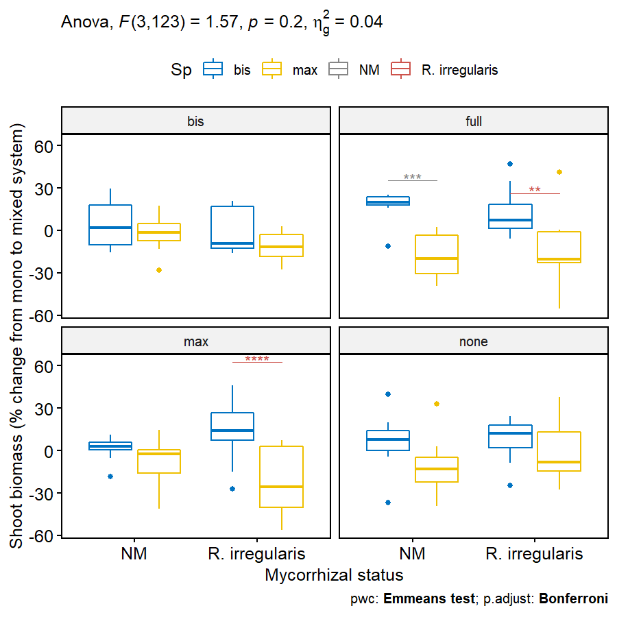 | |
| b) Shoot P response | | |
| 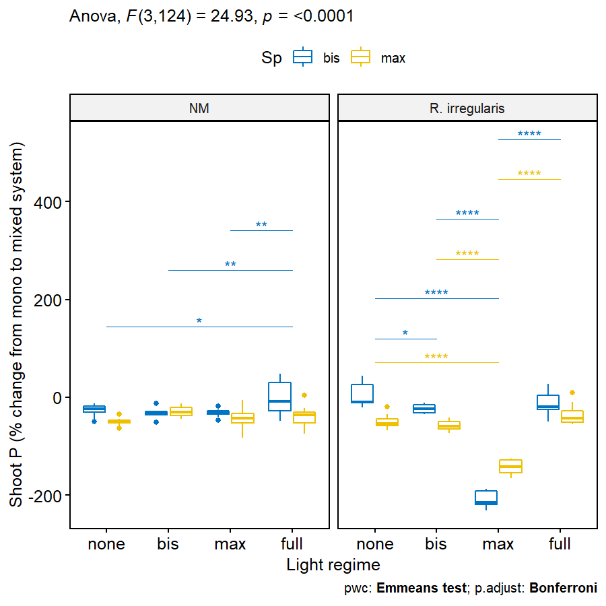 | 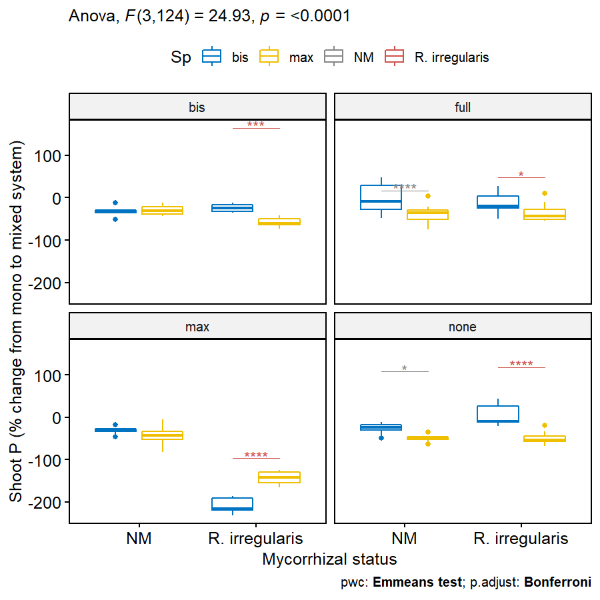 | |
| C) Shoot N response | | |
| 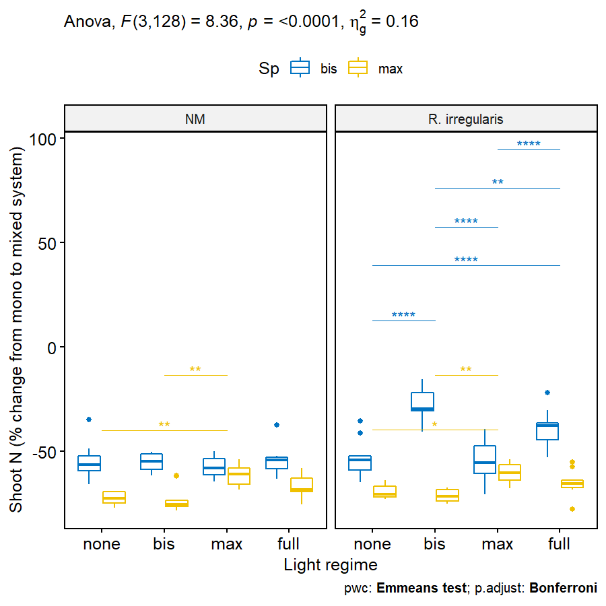 | 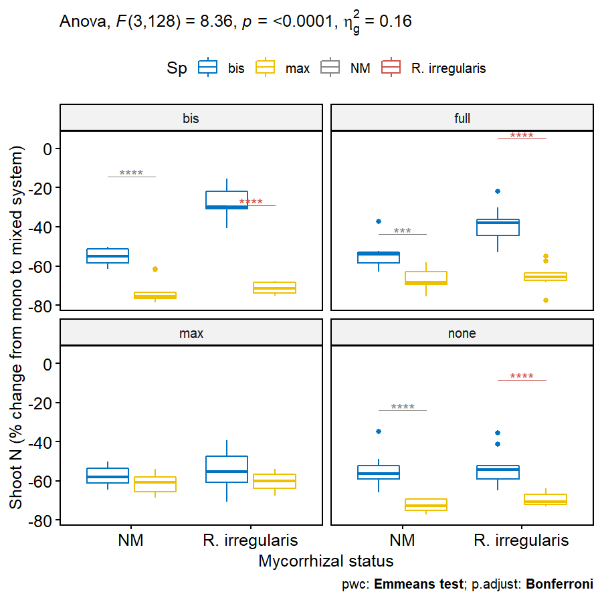 | |
| d) Shoot ^33^P response | | |
| 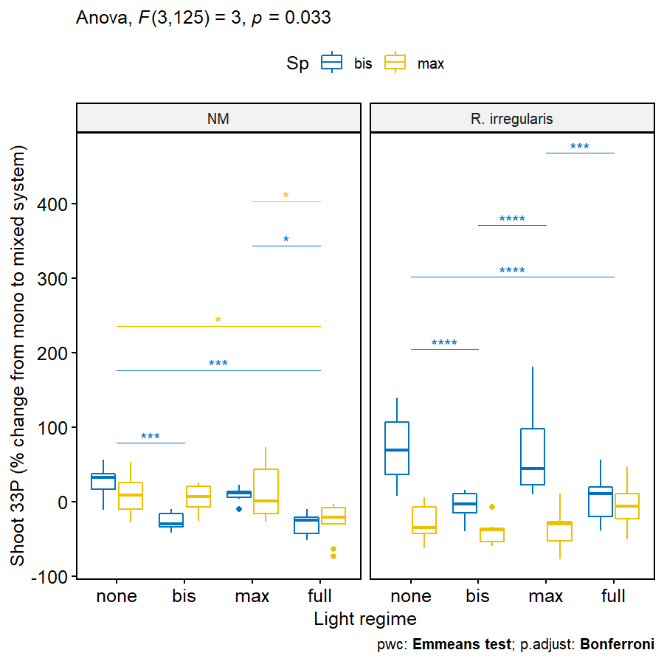 | | 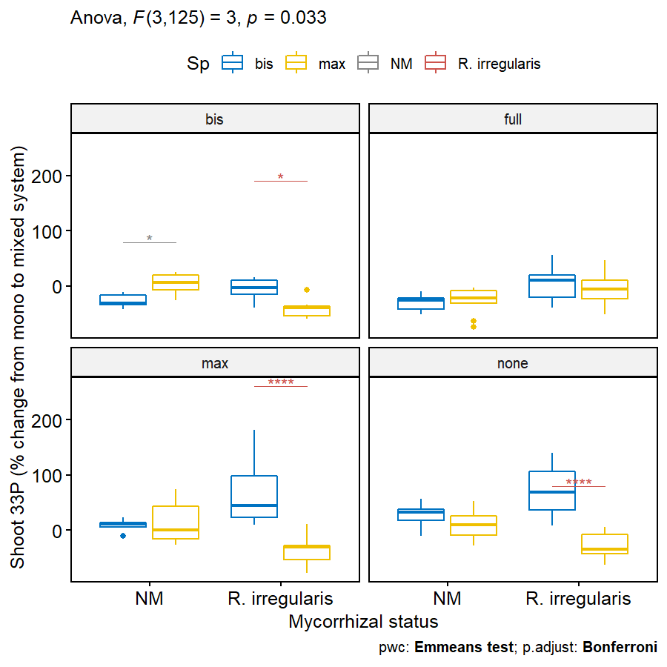 |
| e) Shoot ^15^N response | | |
| 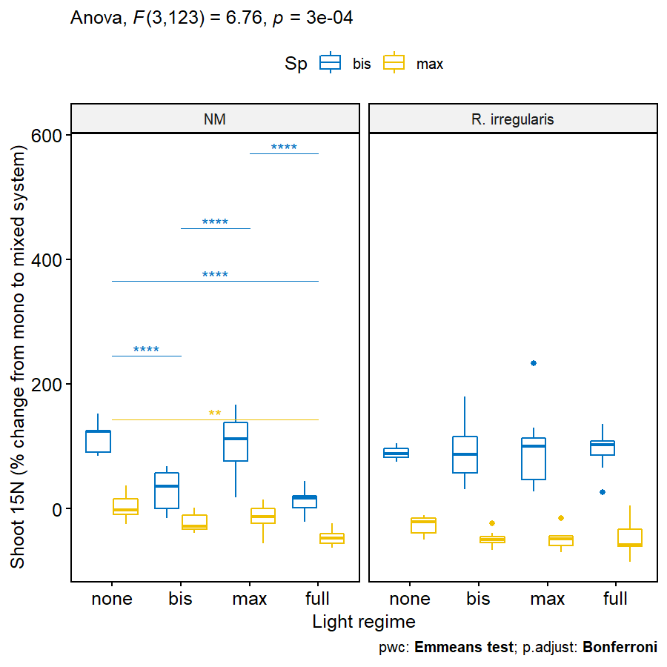 | | 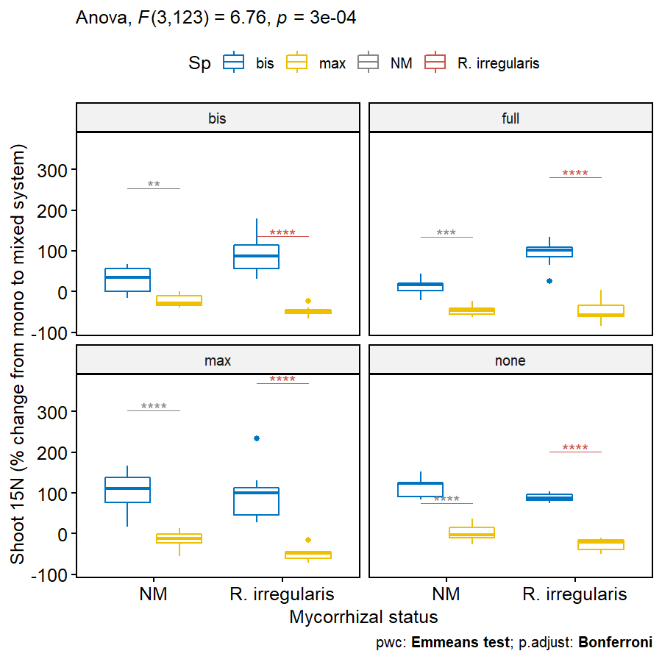 |

**Figure S10.** Results of three-way ANOVA of the effects of light regime, mycorrhizal status, and plant species on shoot biomass- (a), shoot P- (b), shoot N- (c), shoot ^33^P- (d) and shoot ^15^N-responses (e) in the mixed system compared with the mono system. Post-hoc multiple pairwise comparisons between groups were performed using the estimated marginal means and p-values were adjusted using the Bonferroni correction. The ‘’bis’’ and ‘’max’’ refer to *P.maximum* and *P.bisulcatum,* respectively*.* ‘’NM’’ and ‘’R.irregularis’’ refer to the non-mycorrhizal and mycorrhizal (inoculated with *Rhizophagus irregularis*) status of the plants, respectively. Light regime include “none” (no shading), “full” (both plants are shaded), “bis” (P.bisulcatum is shaded) and “max” (P.maximum is shaded). Asterisks indicate levels of significance; p ≤ 0.05 (*), p ≤ 0.01 (**), p ≤ 0.001 (***) and p ≤ 0.0001 (****).

### Root biomass-, P-, N-, ^33^P- and ^15^N-responses

| a) Root biomass response | | |
| --- | --- | --- |
| 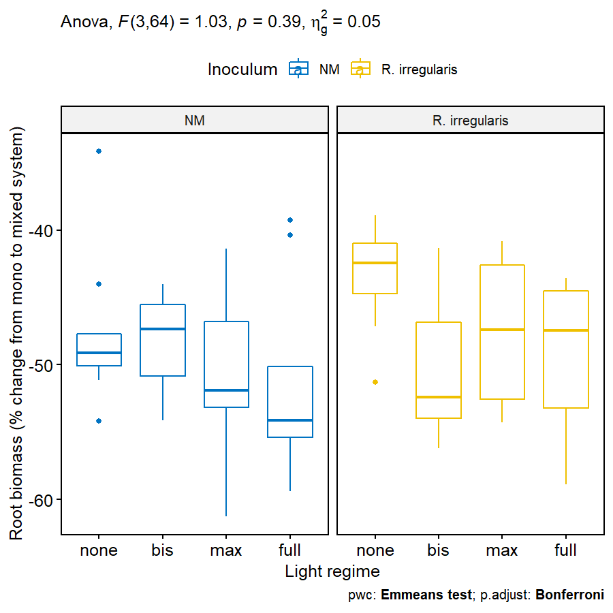 | | 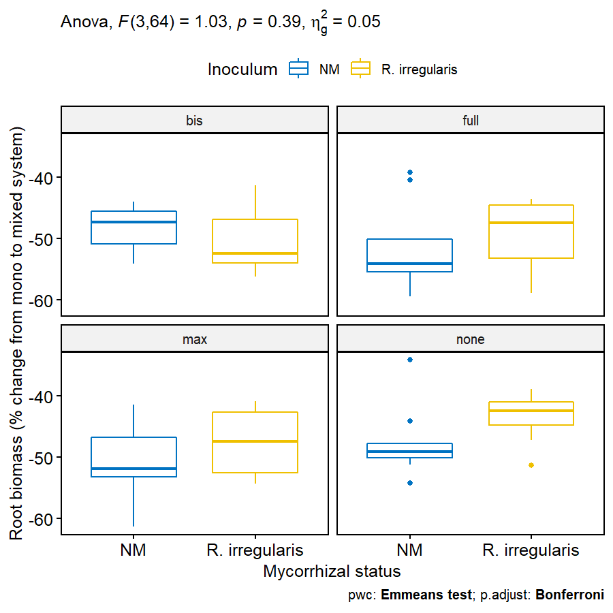 |
| b) Root P response | | |
| 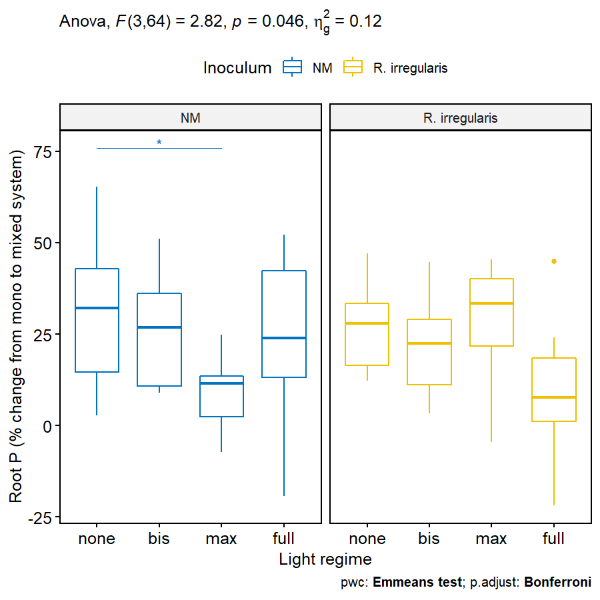 | | 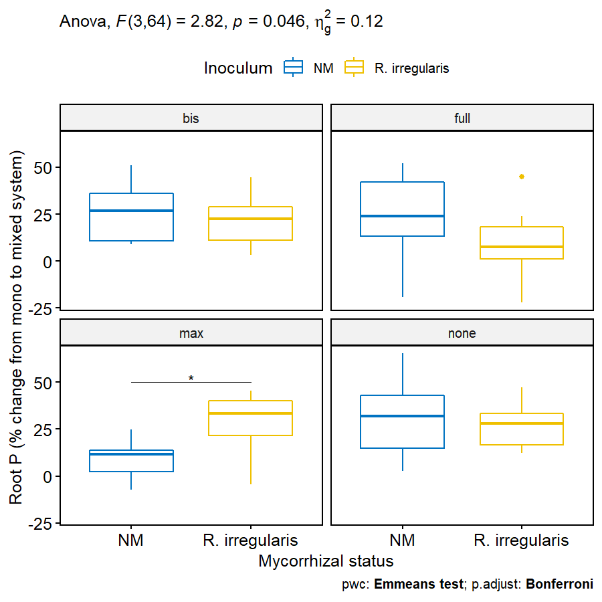 |
| c) Root N response | | |
| 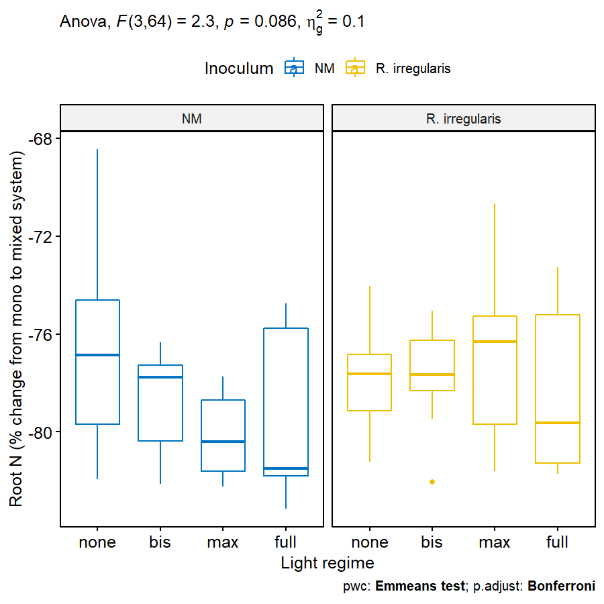 | | 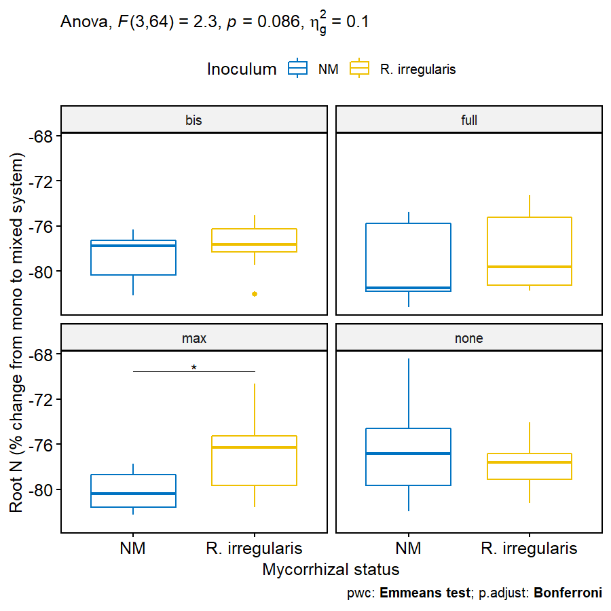 |
| d) Root ^33^P response |  | |
| 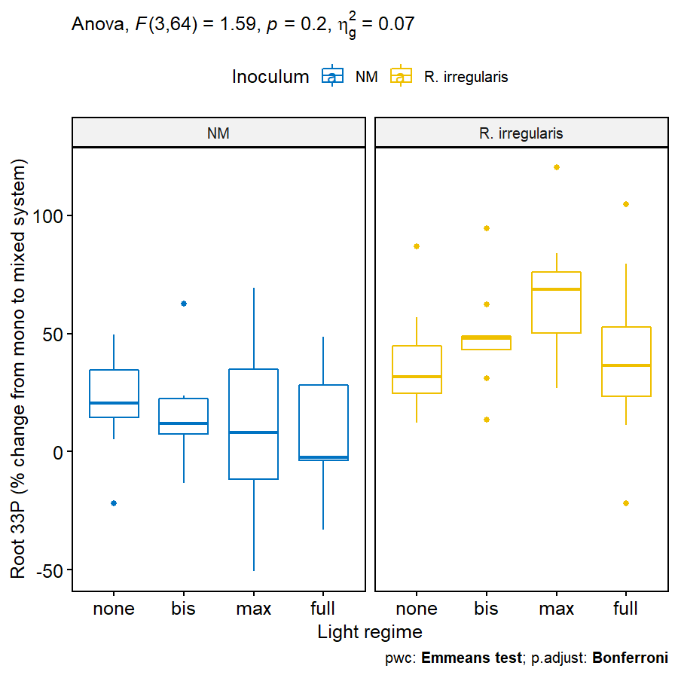 | 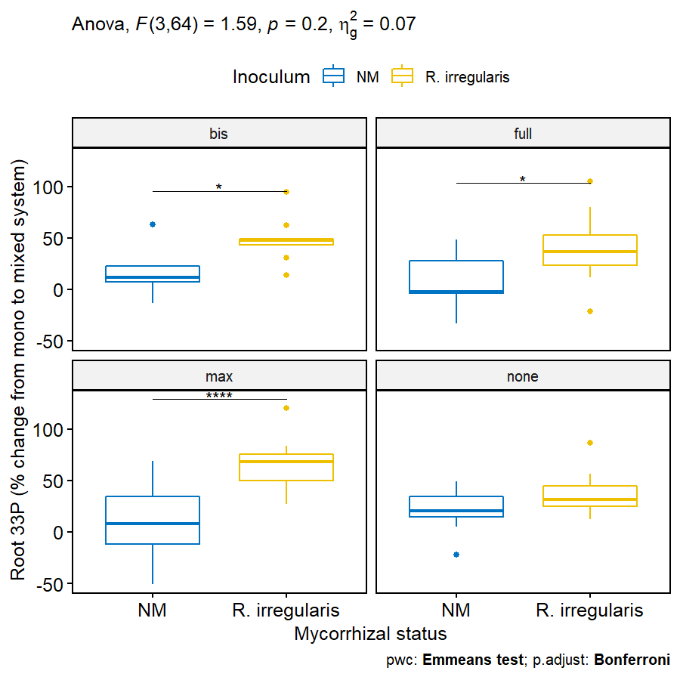 | |
| e) Root ^15^N response |  | |
| 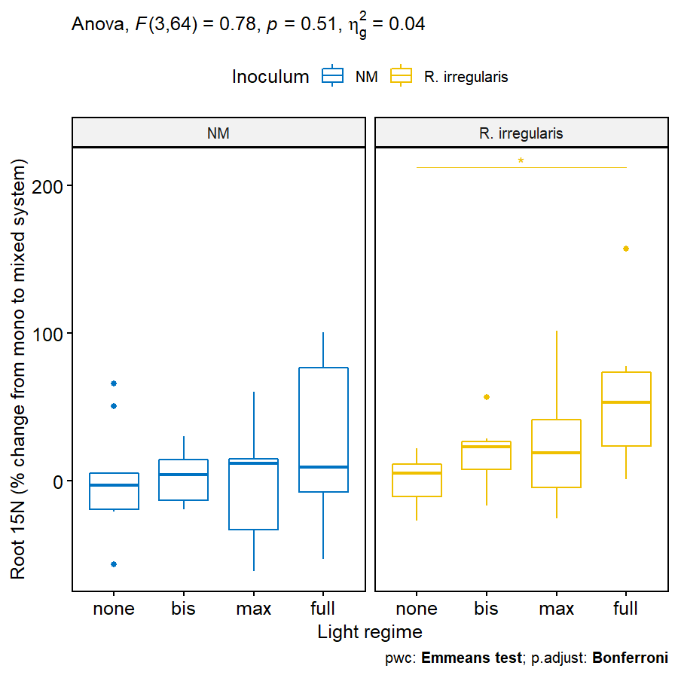 | 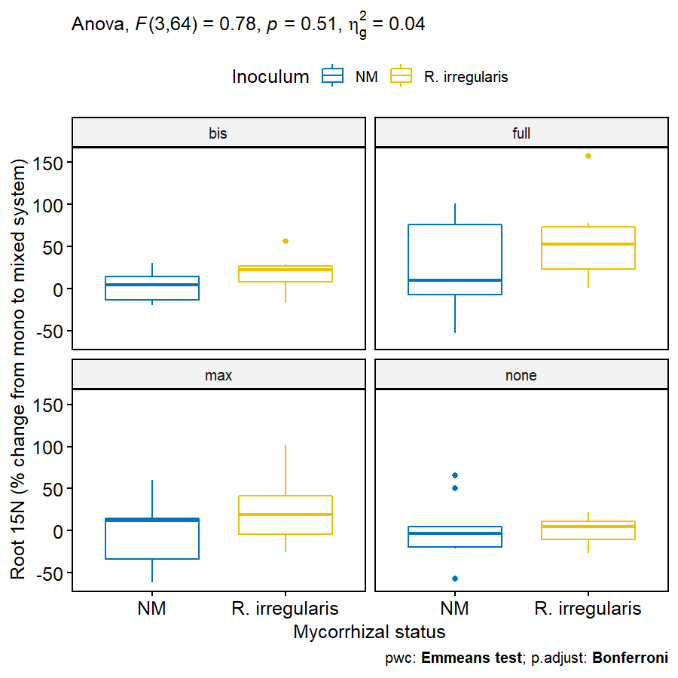 | |

**Figure S11.** Results of two-way ANOVA of the effects of light regime and mycorrhizal status on root biomass- (a), root P- (b), root N- (c), root ^33^P- (d) and root ^15^N-responses (e) in mixed system compared with mono system. Post-hoc multiple pairwise comparisons between groups were performed using the estimated marginal means and p-values were adjusted using the Bonferroni correction. ‘’NM’’ and ‘’R.irregularis’’ refer to the non-mycorrhizal and mycorrhizal (inoculated with *Rhizophagus irregularis*) status of the plants, respectively. Light regime include “none” (no shading), “full” (both plants are shaded), “bis” (P.bisulcatum is shaded) and “max” (P.maximum is shaded). Asterisks indicate levels of significance; p ≤ 0.05 (*), p ≤ 0.01 (**), p ≤ 0.001 (***) and p ≤ 0.0001 (****).

### ^13^C allocation

| a) Total excess ^13^C response | |
| --- | --- |
| 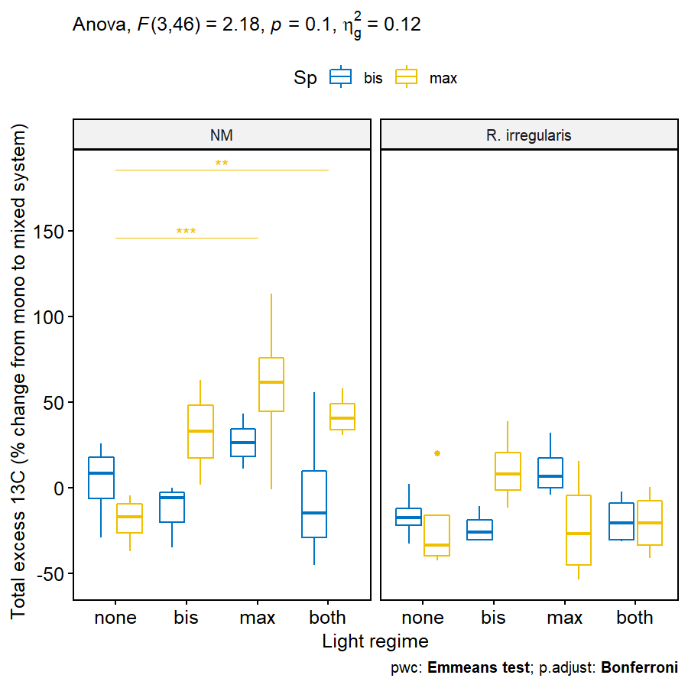 | 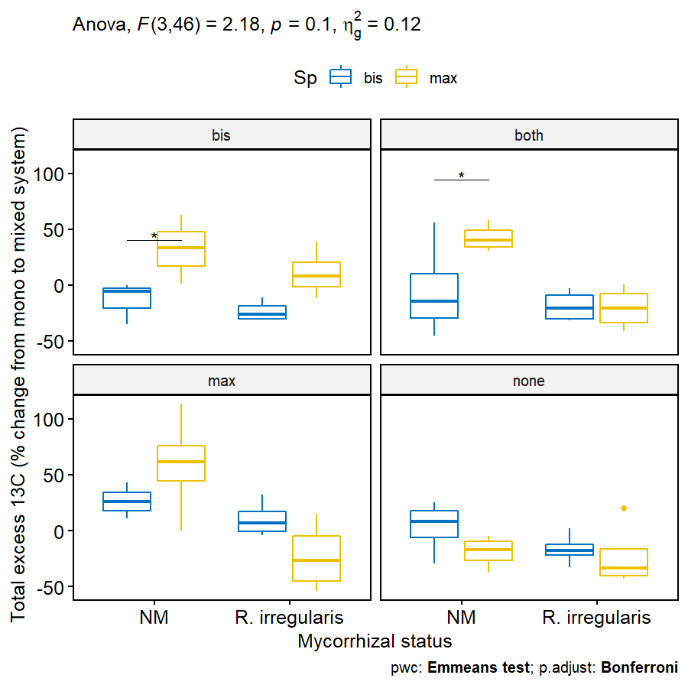 |
| b) ^13^C allocation from shoot to root response | |
| 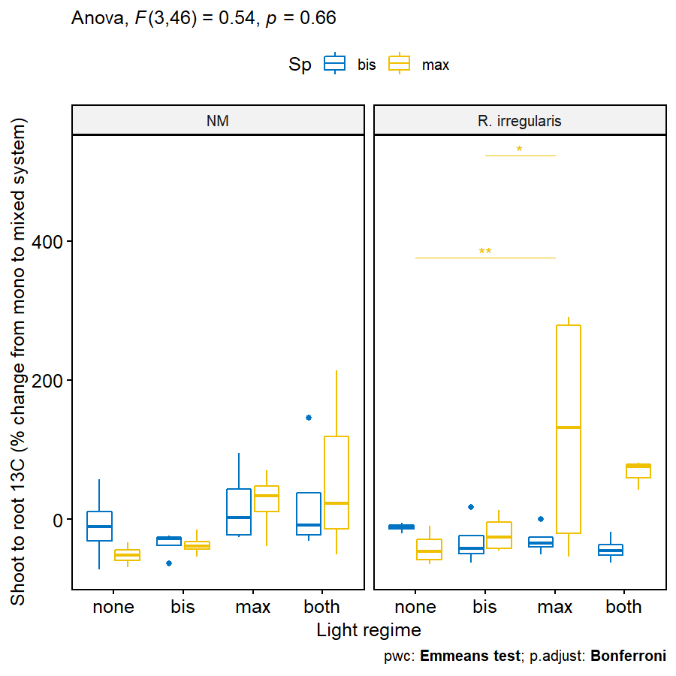 | 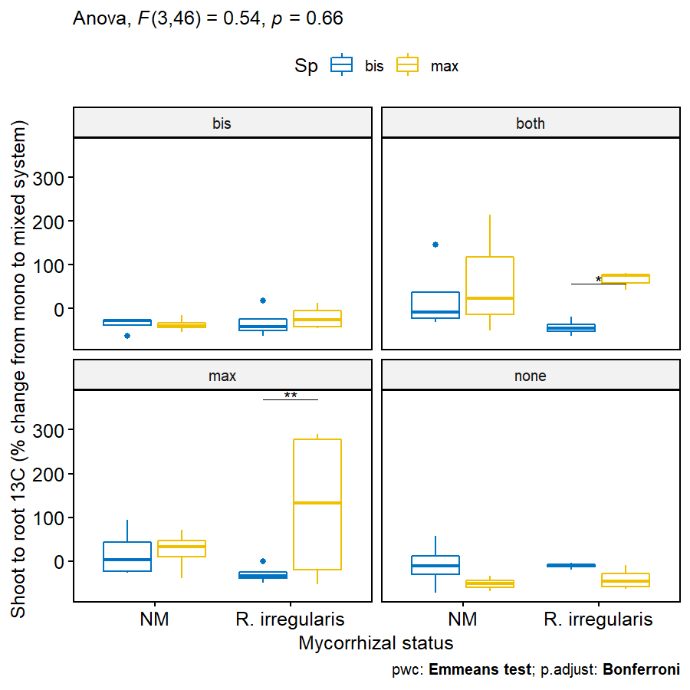 |

| c) ^13^C allocation from aboveground to belowground response | |
| --- | --- |
| 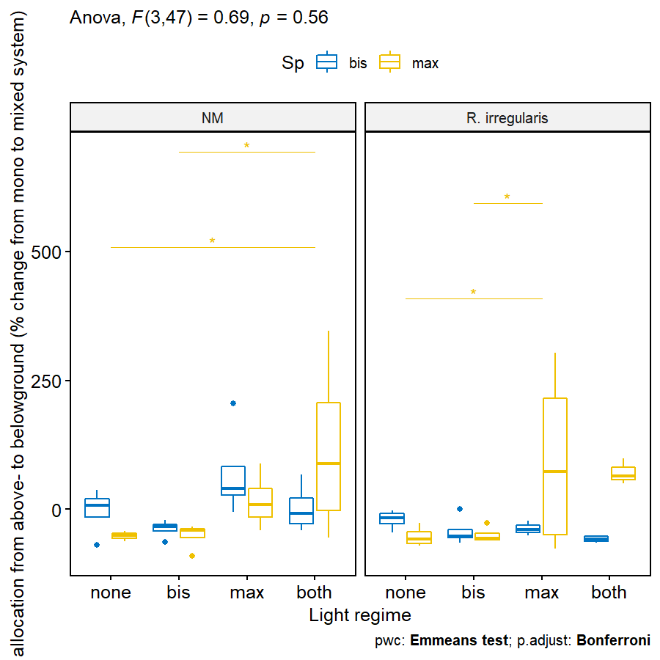 | 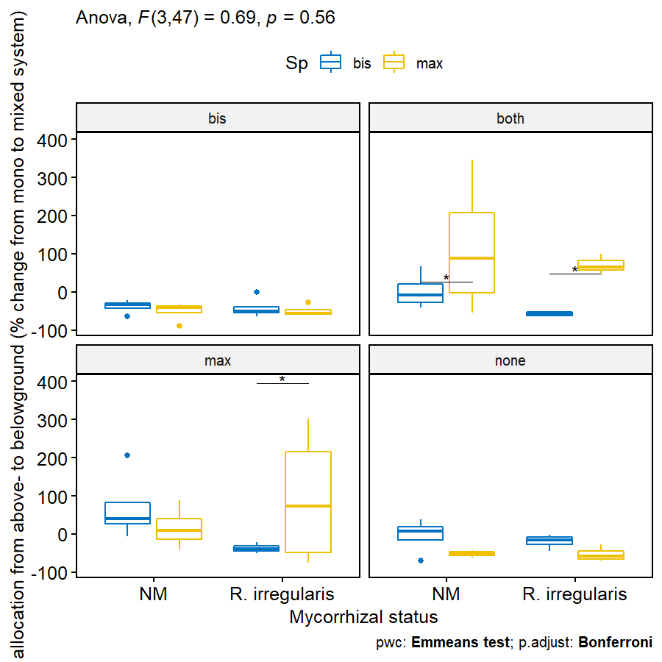 |

**Figure S12.** Results of three-way ANOVA of the effects of light regime, mycorrhizal status, and plant species on excess ^13^C total, ^13^C allocation from shoot to root and ^13^C allocation from aboveground to belowground in mixed system compared to mono system. Post-hoc multiple pairwise comparisons between groups were performed using the estimated marginal means and p-values were adjusted using the Bonferroni correction. The ‘’bis’’ and ‘’max’’ refer to *P.maximum* and *P.bisulcatum,* respectively*.* ‘’NM’’ and ‘’R.irregularis’’ refer to the non-mycorrhizal and mycorrhizal (inoculated with *Rhizophagus irregularis*) status of the plants, respectively. Light regime include “none” (no shading), “full” (both plants are shaded), “bis” (P.bisulcatum is shaded) and “max” (P.maximum is shaded). Asterisks indicate levels of significance; p ≤ 0.05 (*), p ≤ 0.01 (**), p ≤ 0.001 (***) and p ≤ 0.0001 (****).

| a) ^13^C excess in WCFA response |  |
| --- | --- |
| 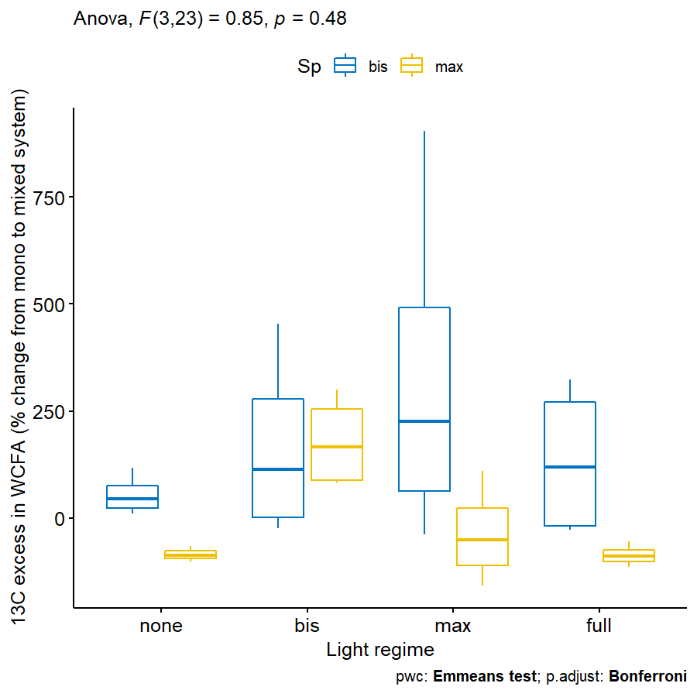 | 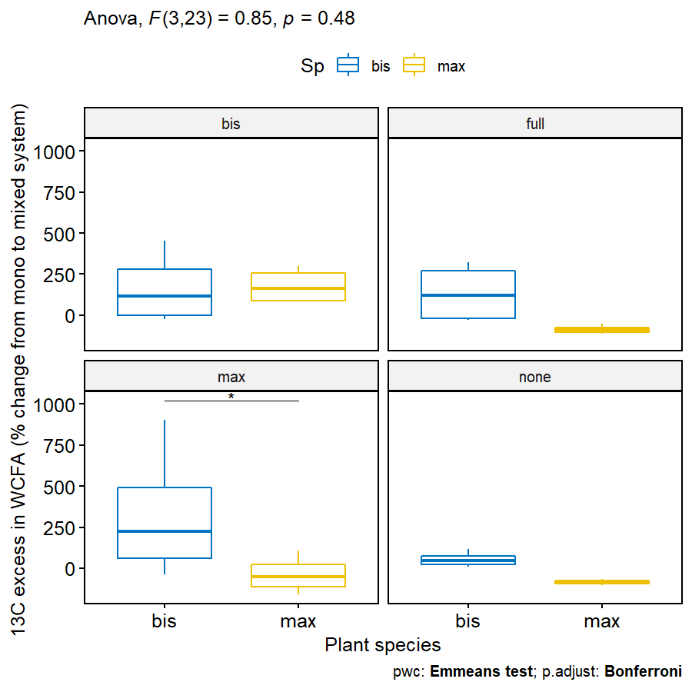 |
| b) ^13^C allocation to C16:1ω5 response |  |
| 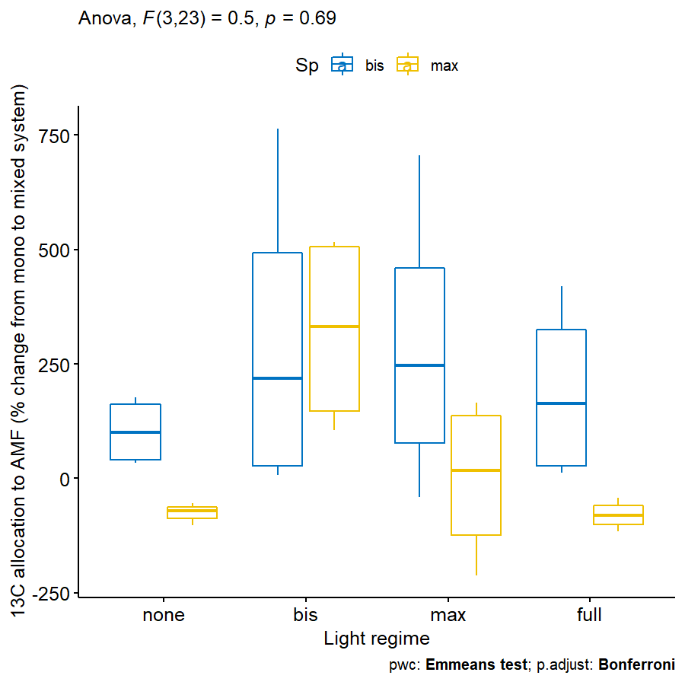 | 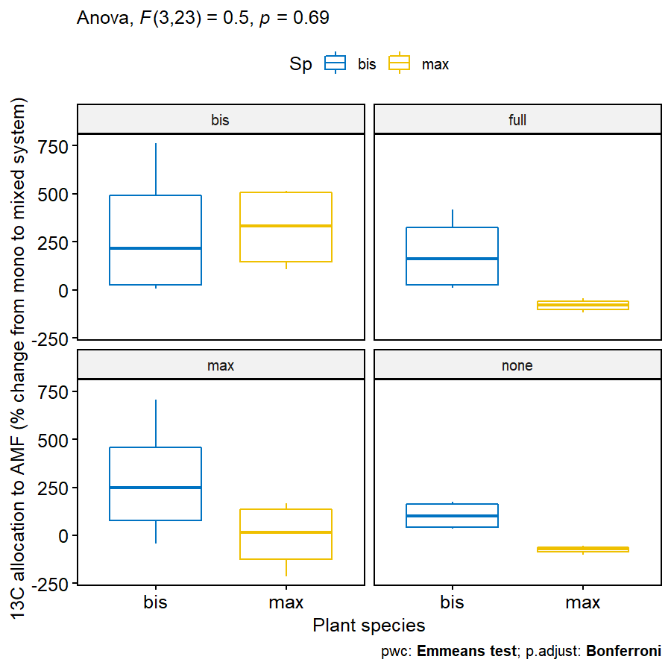 |

**Figure S13.** Results of two-way ANOVA of the effects of light regime and plant species on a) ^13^C excess in C16:1ω5 WCFA (µmol pot^-1^) and b) ^13^C allocation to C16:1ω5 (%) in mixed system compared to mono system. Post-hoc multiple pairwise comparisons between groups were performed using the estimated marginal means and p-values were adjusted using the Bonferroni correction. The ‘’bis’’ and ‘’max’’ refer to *P.maximum* and *P.bisulcatum,* respectively*.* Light regime include “none” (no shading), “full” (both plants are shaded), “bis” (P.bisulcatum is shaded) and “max” (P.maximum is shaded). Only pots inoculated with *Rhizophagus irregularis* were included in this analysis. Asterisks indicate levels of significance; p ≤ 0.05 (*), p ≤ 0.01 (**), p ≤ 0.001 (***) and p ≤ 0.0001 (****).

### Mycorrhizal colonization

| a) AMF abundance in root (qPCR) response | b) AMF abundance in soil (qPCR) response |
| --- | --- |
| 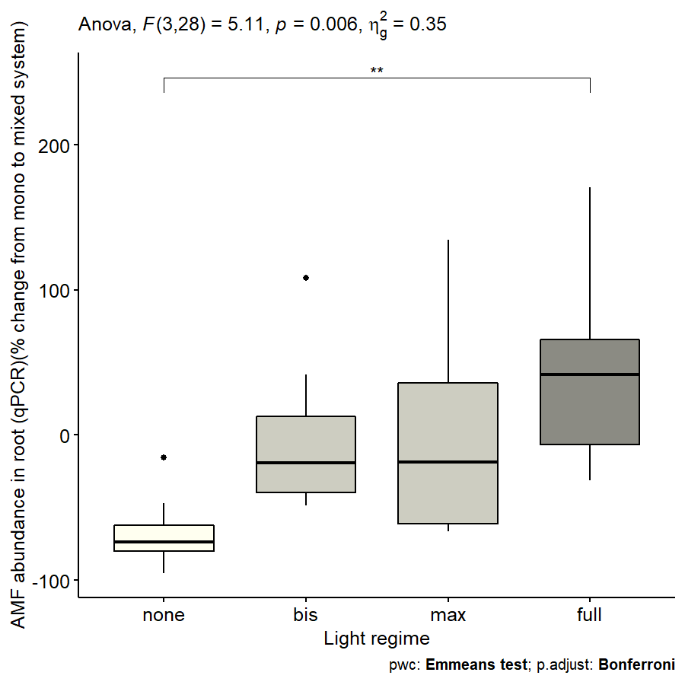 | 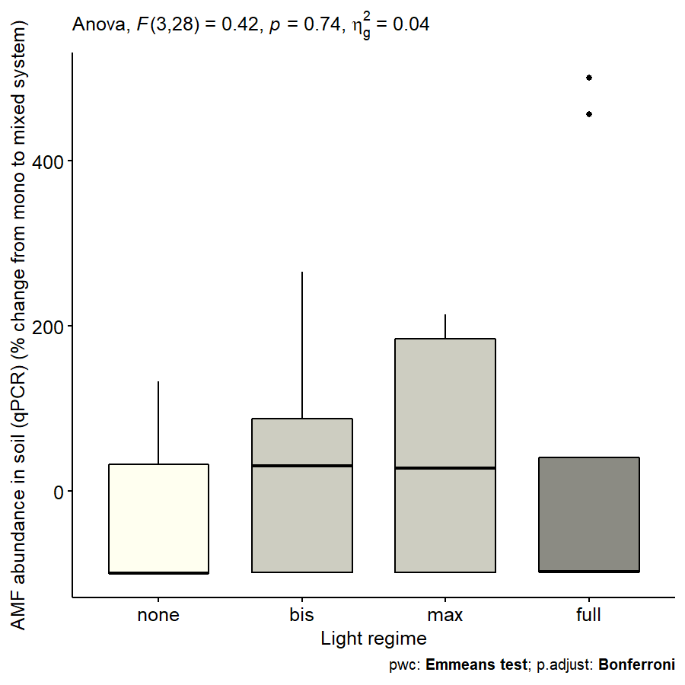 |
| c) AMF abundance in soil response (based on WCFA C16:1ω5 concentration in the soil) | |
| 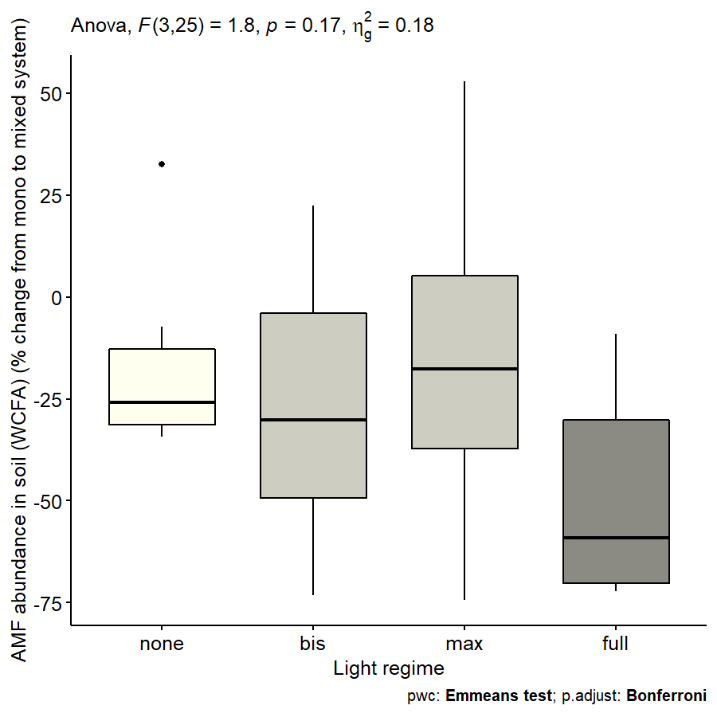 |  |

**Figure S14.** Results of one-way ANOVA of the effects of light regime on AMF abundance in root and soil (per g × 10^6^) of mycorrhizal pots in the mixed system as compared to the mono system. AMF abundance in root and soil was quantified either by quantitative real-time PCR (qPCR) targeting nuclear large ribosomal subunit (LSU) gene of the fungus *R.irregularis or* whole-cell fatty acid analysis (WCFA) using C16:1ω5 biomarker. Light regime include “none” (no shading), “full” (both plants are shaded), “bis” (P.bisulcatum is shaded) and “max” (P.maximum is shaded). Only pots inoculated with *Rhizophagus irregularis* were included in this analysis. Asterisks indicate levels of significance; p ≤ 0.05 (*), p ≤ 0.01 (**), p ≤ 0.001 (***) and p ≤ 0.0001 (****).
